# Cation Disorder and Large Tetragonal Supercell Ordering in the Li-Rich Argyrodite Li_7_Zn_0.5_SiS_6_

**DOI:** 10.1021/acs.chemmater.2c00320

**Published:** 2022-04-18

**Authors:** Bernhard T. Leube, Christopher M. Collins, Luke M. Daniels, Benjamin B. Duff, Yun Dang, Ruiyong Chen, Michael W. Gaultois, Troy D. Manning, Frédéric Blanc, Matthew S. Dyer, John B. Claridge, Matthew J. Rosseinsky

**Affiliations:** † Department of Chemistry, University of Liverpool, Crown Street, L69 7ZD Liverpool, United Kindgom; ‡ Stephenson Institute for Renewable Energy, University of Liverpool, Peach Street, L69 7ZF Liverpool, United Kindgom; § Leverhulme Research Centre for Functional Materials Design, Materials Innovation Factory, Oxford Street, L7 3NY Liverpool, United Kindgom

## Abstract

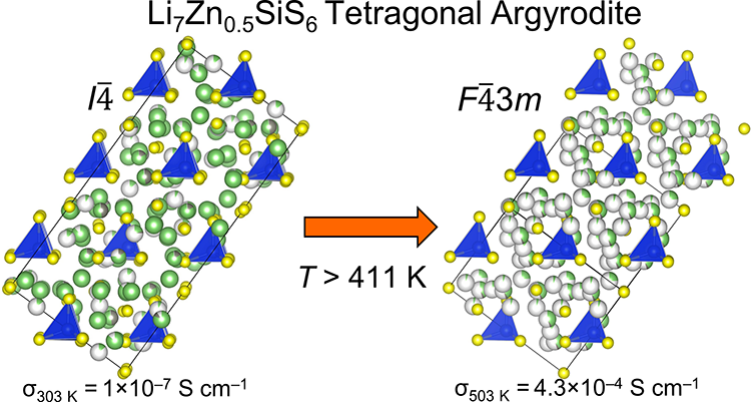

A tetragonal argyrodite with >7 mobile cations, Li_7_Zn_0.5_SiS_6_, is experimentally realized for the first time through solid state synthesis and exploration of the Li–Zn–Si–S phase diagram. The crystal structure of Li_7_Zn_0.5_SiS_6_ was solved *ab initio* from high-resolution X-ray and neutron powder diffraction data and supported by solid-state NMR. Li_7_Zn_0.5_SiS_6_ adopts a tetragonal *I*4 structure at room temperature with ordered Li and Zn positions and undergoes a transition above 411.1 K to a higher symmetry disordered *F*43*m* structure more typical of Li-containing argyrodites. Simultaneous occupation of four types of Li site (T5, T5a, T2, T4) at high temperature and five types of Li site (T5, T2, T4, T1, and a new trigonal planar T2a position) at room temperature is observed. This combination of sites forms interconnected Li pathways driven by the incorporation of Zn^2+^ into the Li sublattice and enables a range of possible jump processes. Zn^2+^ occupies the 48*h* T5 site in the high-temperature *F*43*m* structure, and a unique ordering pattern emerges in which only a subset of these T5 sites are occupied at room temperature in *I*4 Li_7_Zn_0.5_SiS_6_. The ionic conductivity, examined via AC impedance spectroscopy and VT-NMR, is 1.0(2) × 10^–7^ S cm^–1^ at room temperature and 4.3(4) × 10^–4^ S cm^–1^ at 503 K. The transition between the ordered *I*4 and disordered *F*43*m* structures is associated with a dramatic decrease in activation energy to 0.34(1) eV above 411 K. The incorporation of a small amount of Zn^2+^ exercises dramatic control of Li order in Li_7_Zn_0.5_SiS_6_ yielding a previously unseen distribution of Li sites, expanding our understanding of structure–property relationships in argyrodite materials.

## 1 Introduction

**Figure 1:**
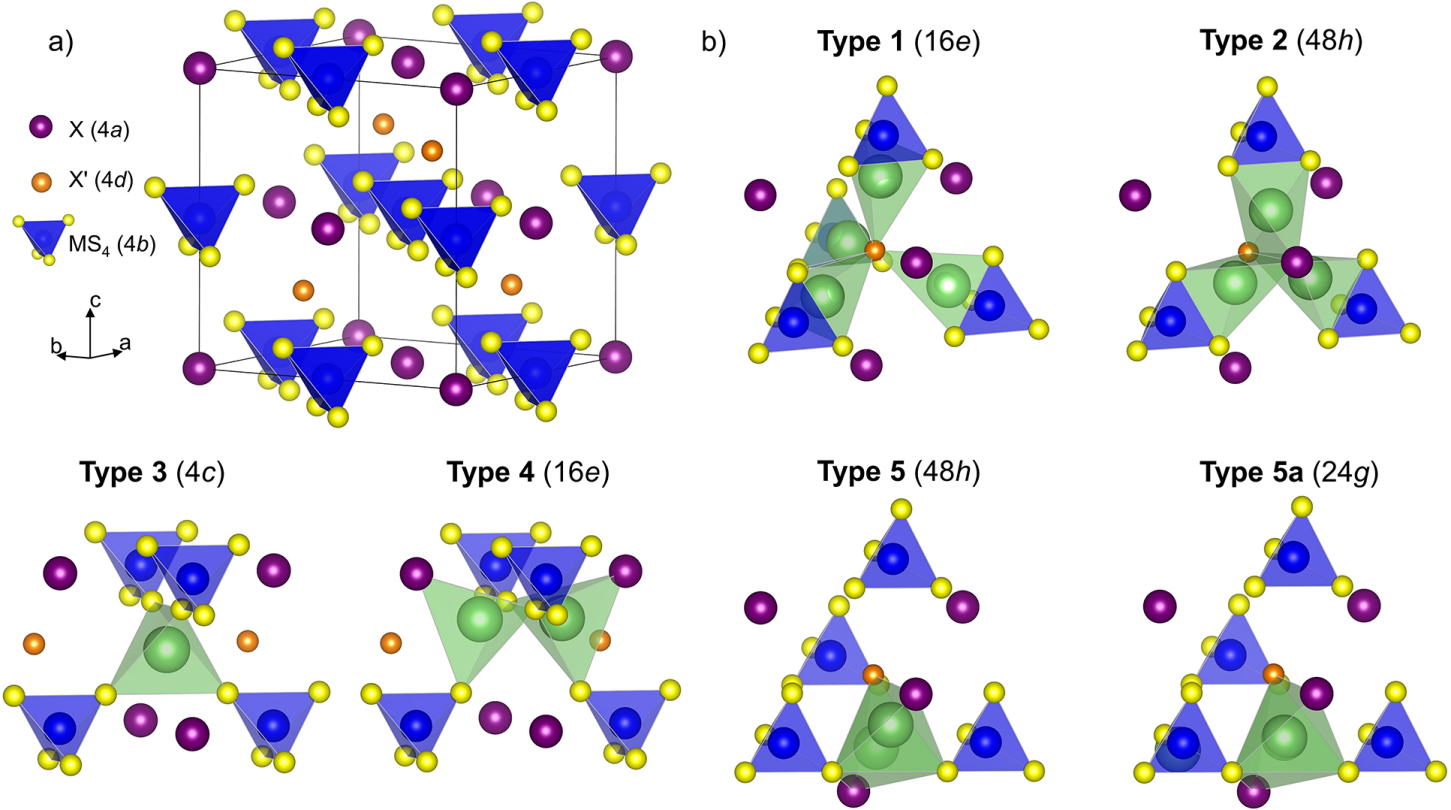
(a) Unit cell of Li_6_MS_4_XX′ cubic *F*43*m* argyrodite (M = Si^4+^, Ge^4+^, P^5+^; X = S^2–^, O^2–^, Cl^–^, Br^–^, I^–^) with Li atoms removed. MS_4_ polyanions have a cubic close-packed arrangement while X anions occupy octahedral voids and X′ anions occupy half of the tetrahedral voids between the MS_4_ tetrahedra. (b) Five types of vacant tetrahedral voids can be occupied by Li^+^ and their Wyckoff positions.^5,7^ T5a is a trigonal planar position that occupies the shared face between two T5 tetrahedra. Reproduced from *Acc. Chem. Res.***2021**, *54*, 2717–2728 (ref (5)). Copyright 2021 American Chemical Society.

The argyrodite family of materials, related to the mineral Ag_8_GeS_6_, exhibit considerable compositional flexibility and have been extensively studied for various applications including fast ion conduction (e.g., Ag_7_GeSe_5_I and Cu_6_PS_5_Cl),^1,2^ thermoelectrics (e.g., Cu_8_GeS_6_ and Ag_8_SnSe_6_),^3,4^ and nonlinear optical (Cd_3.25_PS_5.5_I_0.5_) materials.^1^ The degree of structural disorder, which can be controlled through cationic or anionic substitution, determines the properties of argyrodite materials, so reliable characterization of such crystal structures is critical to expanding our knowledge of these systems.^5^ The crystal structures of argyrodites such as Li_6_PS_5_X (X = Cl, Br) have tetrahedral close packed topologies related to that of the Laves phases (e.g., MgCu_2_) with high symmetry aristotype argyrodite polymorphs adopting cubic *F*43*m* symmetry.^1^ Recently, we demonstrated that the argyrodite structure can also be considered equivalent to that of antiperovskite through anion and vacancy ordering within a cubic stacking of two close-packed layers that enabled the discovery of a hexagonal argyrodite Li_6_SiO_4_Cl_2_.^6^ The argyrodite Li_6_MS_4_XX′ structure can be described for sulfides as a cubic close-packed arrangement of MS_4_ tetrahedral polyanions (where M can be Si^4+^, P^5+^, Ge^4+^, etc.) with individual X and X′ anions (X/X′ = S^2–^, O^2–^, Cl^–^, Br^–^, I^–^) occupying the octahedral and half of the tetrahedral voids, respectively, as shown in Figure 1a. This generates five distinct types of tetrahedral void (Figure 1b) that can be occupied by Li^+^ cations,^5,7,8^ and in high symmetry *F*43*m* argyrodite structures, the Li^+^ cations can be ordered such as in Li_6_PO_5_X (X = Cl, Br) or dynamically disordered as observed in Li_6_PS_5_X (X = Cl, Br).^9,10^ This dynamic disorder is manifested with Li^+^ ions delocalized across several lattice positions (commonly, the 24*g* and 48*h* Wyckoff sites frequently referred to as T5a and T5 sites, respectively),^5^ which leads to reduced activation barriers for ion mobility in these systems, with Li_6_PS_5_Br exhibiting an ionic conductivity of 6.8 × 10^–3^ S cm^–1^ at room temperature.^11^ In contrast, despite adopting *F*43*m* symmetry, Li_6_PO_5_Br has a room-temperature conductivity of ∼10^–9^ S cm^–1^, which results from statically ordered Li^+^ positions on the 24*g* (T5a) site, and an order–disorder transition is not observed for Li_6_PO_5_Br in the temperature range of 173–873 K.^10^ Dynamic Li^+^ disorder leading to superionic performance is common in mixed sulfide/halide argyrodites such as Li_6_PS_5_X (X = Cl, Br) in which a high degree of sulfide/halide disorder modifies the anionic charge distribution, leading to significantly delocalized lithium positions,^5,12^ and also in mixed cation materials such as Li_6+*x*_Sb_1–*x*_Sn_*x*_S_5_I and Li_6+*x*_P_1–*x*_Si_*x*_S_5_Br where aliovalent substitution increases Li^+^ carrier concentration and creates high-energy interstitial sites that participate in Li^+^ diffusion.^13,14^

Lower symmetry argyrodite polymorphs are stabilized when the Li^+^ cations are ordered, as in Li_7_PCh_6_ (Ch = S, Se), which adopt orthorhombic *Pna*2_1_ symmetry at room temperature and are isostructural to α-Cu_7_PSe_6_.^7,15,16^ Both Li_6_PS_5_I and Li_6_AsS_5_I adopt *Cc* symmetry at temperatures below 180 and 173 K, respectively, and are isostructural to Cu_6_PS_5_Br with full Li^+^ occupancy of either tetrahedral (T5) or trigonal planar (T5a) sites (4*a* Wyckoff sites in *Cc*) to yield ordered positions.^7,17^ These orthorhombic (*Pna*2_1_) and monoclinic (*Cc*) symmetries are subgroups of *F*43*m*, and the materials exhibit a transition to cubic higher symmetry disordered structures with significant enhancement in ion transport above these temperatures. In the high symmetry (*F*43*m*) structures, Li^+^ ions are disordered with partial occupancies of tetrahedral (48*h*, T5) positions for Li_7_PCh_6_ (Ch = S, Se) and Li_6_AsS_5_I and both tetrahedral (48*h*, T5) and trigonal (24*g*, T5a) positions for Li_6_PS_5_I. Three other structural symmetries are commonplace in non-Li argyrodite materials: orthorhombic *Pmn*2_1_ as observed for Cu_8_SiCh_6_ (Ch = S and Se), hexagonal *P*6_3_*cm* (e.g., for Cu_8_GeS_6_), and cubic *P*2_1_3 as adopted by Ag_7_PS_6_ and Cu_7_PS_6_.^1,3^

Here, we report the discovery and synthetic isolation of new Li_7_Zn_0.5_SiS_6_ as the first argyrodite material reported with a tetragonal (*I*4) crystal structure. A reversible transition is observed through differential scanning calorimetry and variable temperature X-ray and neutron powder diffraction above 411 K where Li_7_Zn_0.5_SiS_6_ exhibits a disordered cubic *F*43*m* structure analogous to other argyrodites that leads to a significant decrease in activation energy for Li^+^ ion transport to 0.34(1) eV from 0.66(1) eV for the ordered room-temperature *I*4 structure. Zn^2+^ cations occupy a single lattice site in the *F*43*m* structure shared with Li^+^, but only a small subset of these sites is occupied by Zn^2+^ at room temperature in the *I*4 structure. This addition of a small amount of Zn^2+^ drives the Zn and Li ordering to generate the lower symmetry tetragonal *I*4 structure at room temperature. Ionic conductivity is measured via AC impedance spectroscopy and Li^+^ ion mobility assessed via NMR as a local probe.

## 2 Experimental Section

### 2.1 Exploratory Synthesis in the LiS_0.5_-ZnS-SiS_2_ Phase Field

All reagents and samples were handled under inert helium atmosphere (O_2_ < 1 ppm). Solid state reactions were carried out by flame sealing reagent mixtures (typically, 300 mg for each reaction) inside carbon-coated evacuated quartz ampules (<10^–4^ mbar). Stoichiometric mixtures of lithium sulfide (Li_2_S, Sigma-Aldrich, 99.98%), zinc sulfide (ZnS, Sigma-Aldrich, 99.99%), silicon powder (Si, Alfa Aesar, 325 mesh, 99.5%), and elemental sulfur (S, Sigma-Aldrich, 99.999%) were used as provided and thoroughly ground for 15 min using an agate pestle and mortar. For initial exploratory reactions in the LiS_0.5_-ZnS-SiS_2_ phase field, these reaction mixtures were heated to 673 K at a heating rate of 5 K min^–1^, then heated to 973 K at a slower rate of 0.5 K min^–1^, and held at 973 K for 24 h before being cooled to ambient temperature at a rate of 5 K min^–1^. Resulting powders were ground before being fired again to 973 K for 24 h using a heating and cooling rate of 5 K min^–1^.

### 2.2 Synthesis of Li_7_Zn_0.5_SiS_6_

Powders of Li_7_Zn_0.5_SiS_6_ were obtained using stoichiometric mixtures of the above starting materials sealed in carbon-coated evacuated quartz ampules, which were fired to 973 K for 24 h using a heating and cooling rate of 5 K min^–1^ twice with the powders ground in between the two firings. This reaction procedure yielded phase pure samples of Li_7_Zn_0.5_SiS_6_ as assessed by laboratory powder X-ray diffraction (PXRD) data.

### 2.3 Powder Diffraction

Routine PXRD analysis of phase purity and lattice parameters was performed on a Bruker D8 Advance diffractometer with a monochromatic Cu X-ray source (Kα_1_, λ = 1.54056 Å) or Mo X-ray source (Kα_1_, λ = 0.70932 Å) in Debye–Scherrer geometry. Powder samples were sealed inside borosilicate glass capillaries. Structure determination and Rietveld refinements were carried out on synchrotron X-ray diffraction (SXRD) data collected at the I11 beamline (Diamond Light Source, U.K.) with an incident wavelength of 0.824878(10) Å. High-resolution data were collected at room temperature using the multianalyzer crystal (MAC) detectors. Samples were sealed inside Ø = 0.3 mm borosilicate capillaries. Variable temperature SXRD measurements from ambient temperature to 448 K in 25 K steps were carried out on beamline I11 using an Oxford Cryostream Plus with the Mythen position sensitive detector (PSD). Data were collected on heating using a heating and cooling rate of 10 K min^–1^.

Time-of-flight neutron powder diffraction (NPD) data were collected at ambient temperature (300 K) and at 448 K using the low temperature furnace on the Polaris instrument at ISIS, the U.K. spallation neutron source. Powders were loaded into thin-walled vanadium metal cans of 6 mm diameter under an inert helium atmosphere and sealed using a copper gasket. Data were collected on a ^7^Li enriched sample to minimize the impact of absorption using a ^7^Li_2_S precursor, which was synthesized from ^7^Li_2_CO_3_ (Sigma-Aldrich, 99%) that was heated to 923 K under flowing CS_2_ vapor for 6 h.^18^

### 2.4 Elemental Analysis

Elemental analysis of Li_7_Zn_0.5_SiS_6_ was carried out by Mikroanalytisches Labor Pascher (Remagen-Bandorf, Germany). The powder was dissolved, and elements were detected by inductively coupled plasma atomic emission spectroscopy (ICP-AES) using a Thermo Fisher Scientific iCap 6500 instrument.

### 2.5 Differential Scanning Calorimetry (DSC)

Heat flux profiles were measured from 17 mg of powdered sample in a 100 μL Ni/Cr crucible sealed under a helium atmosphere (O_2_ < 1 ppm) using a Netzsch DSC 404 F1 differential scanning calorimeter. Data were recorded on heating from 303 to 773 K and then cooling to 303 K using a heating and cooling rate of 1 K min^–1^ under a constant 50 mL min^–1^ flow of helium. The temperature of the observed transition is the average of the values obtained from both the heating and cooling data sets, which were extracted through peak fitting.

### 2.6 Raman Spectroscopy

Raman spectra were collected on samples sealed under a helium atmosphere in borosilicate glass capillaries (O_2_ < 1 ppm) using an inVia Reflex Qontor Confocal Raman Microscope from Renishaw with a laser excitation wavelength of 523 nm.

### 2.7 Alternating Current (AC) Impedance Spectroscopy and Direct Current (DC) Polarization

A pellet of Li_7_Zn_0.5_SiS_6_ was prepared by uniaxially pressing ∼30 mg of materials with a 5 mm cylindrical steel die at a pressure of 125 MPa and subsequent sintering at 923 K for 14 h using heating and cooling rates of 5 K min^–1^. Using this method, a density of 81% was achieved. The pellet faces were sputtered with gold, which were used as ion-blocking electrodes. Temperature dependent AC-impedance measurements were carried out by heating the sample from 303 to 503 K in an Ar-filled glovebox (O_2_ < 1 ppm, H_2_O < 1 ppm) at a rate of 3 K min^–1^. Measurements were recorded in 20 K steps following a 10 min equilibration period at each target temperature. Impedance data were recorded using a Keysight Impedance Analyzer E4990A. A sinusoidal amplitude of 50 mV was employed in the frequency range of 100 MHz to 100 mHz. Impedance data were fitted with an equivalent circuit using the program ZView2.^19^

The same pellet used for AC impedance measurements was used for DC polarization measurements. DC polarization data were collected at ambient conditions on an Au|Li_7_Zn_0.5_SiS_6_|Au symmetric cell using potentiostatic polarization measurements between 0.05 and 1.0 V for 2 h and monitoring the current variation with time using an Autolab 84515 instrument.

### 2.8 Nuclear Magnetic Resonance (NMR)

^6^Li and ^29^Si magic angle spinning (MAS) NMR experiments were recorded at room temperature on a 9.4 T Bruker DSX spectrometer equipped with a 4 mm HXY MAS probe (in double resonance mode) with the X channel tuned to ^6^Li at ω_0_/2π(^6^Li) = 59 MHz or ^29^Si at ω_0_/2π(^29^Si) = 79.5 MHz. 90° pulses of duration 3 μs at a radiofrequency (rf) amplitude of ω_1_/2π = 83 kHz were used. The MAS frequency ω_r_/2π was set to 10 kHz. All spectra were obtained under quantitative recycle delays of more than 5 times longer than the respective longitudinal relaxation times *T*_1_ measured through the saturation recovery pulse sequence, and the data were fitted with a stretch exponential function in the form of 1 – exp[−(τ/*T*_1_)^α^] (with α values of 0.9 for the ^6^Li and ^29^Si data sets).

Static ^7^Li variable temperature (VT) NMR experiments on a sample sealed in a glass ampule were recorded on a 9.4 T Bruker Avance III HD spectrometer equipped with a 4 mm HXY MAS probe (in double resonance mode) with the X channel tuned to ^7^Li at ω_0_/2π(^7^Li) = 156 MHz. All ^7^Li NMR spectra were recorded with a 90° pulse at rf field amplitude of ω_1_/2π = 83 kHz and under quantitative recycle delays of more than 5 times longer than the *T*_1_ time measured through the saturation recovery pulse sequence with data also fitted to the same stretch exponential function as above (with α ranging from 0.8 to 0.95). The stretch exponential was used in order to account for any distribution of correlation times and temperature gradients across the sample.

The temperature calibration of the probe was carried out using the ^207^Pb NMR chemical shift thermometer Pb(NO_3_)_2_.^20^ The temperature standard error associated with this method arises from the temperature gradients across the sample, which ranged from 2 to 8 K.

## 3 Results and Discussion

### 3.1 Synthesis and Isolation of New Li_7_Zn_0.5_SiS_6_

**Figure 2:**
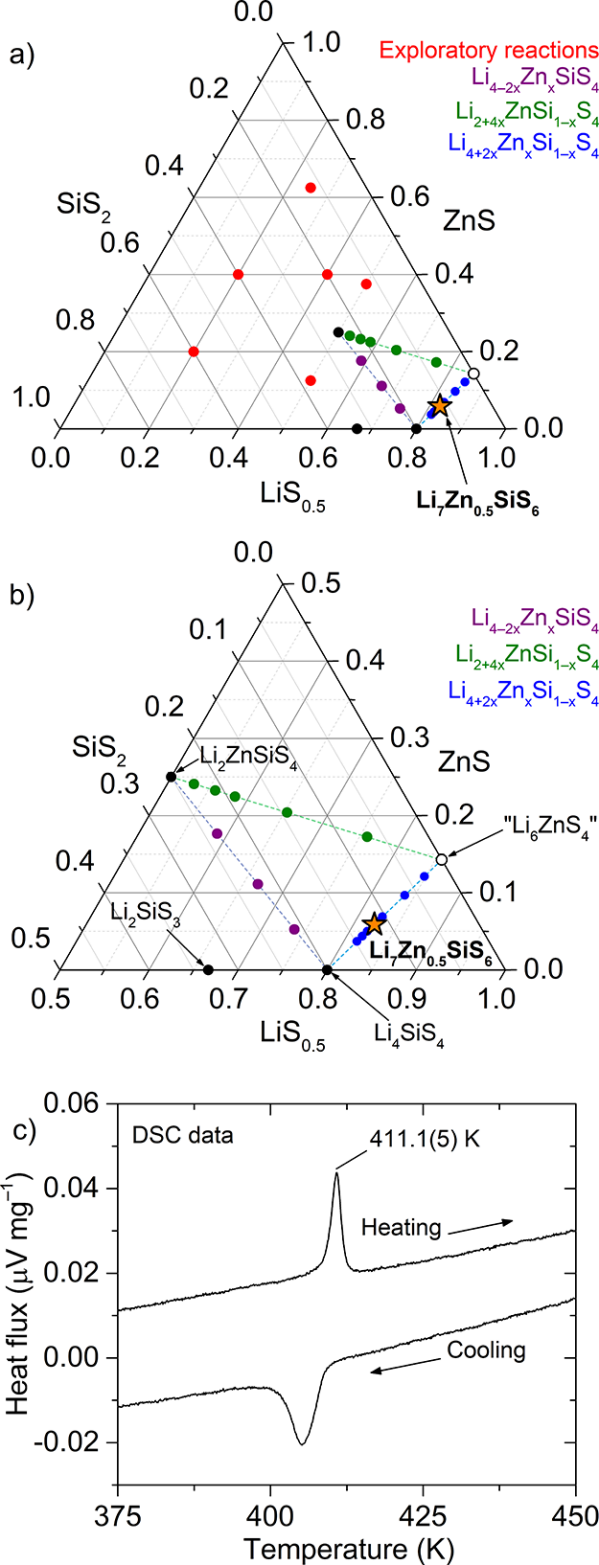
(a) Pseudoternary LiS_0.5_–ZnS–SiS_2_ phase field explored by synthesizing the compositions shown. (b) Enlargement of the lower-right corner of the LiS_0.5_–ZnS–SiS_2_ phase field highlighting greater exploration of the Li-rich region. Known phases are shown as black circles and are labeled. Red circles in (a) indicate exploratory compositions. Purple, green, and blue circles in (a) and (b) show compositions explored on the Li_4–2*x*_Zn_*x*_SiS_4_, Li_2+4*x*_ZnSi_1–*x*_S_4_, and Li_4+2*x*_Zn_*x*_Si_1–*x*_S_4_ tie lines, respectively. The orange star indicates new Li_7_Zn_0.5_SiS_6_. (c) DSC data show a reversible thermal event associated with a phase transition from tetragonal to cubic symmetry in Li_7_Zn_0.5_SiS_6_.

The LiS_0.5_–ZnS–SiS_2_ phase field was explored using solid state methods with reagent mixtures sealed inside the carbon-coated evacuated quartz ampules (<10^–4^ mbar). Syntheses were carried out within a range of reaction temperatures (873–973 K) with all starting materials and resulting powders handled under an inert helium atmosphere (O_2_ < 1 ppm). First, a selection of compositions within the phase field were synthesized, and the resulting phases were carefully matched to known materials through analysis of PXRD data (Figure 2a, red points). The majority of the synthesized compositions contained mixtures of binary sulfides and Li_2_ZnSiS_4_, which is the only quaternary that has been previously characterized within the LiS_0.5_–ZnS–SiS_2_ phase field.^21^ Second, to explore the Li-rich composition space of the phase field at greater granularity, compositions within solid solutions between the end member materials Li_4_SiS_4_, Li_2_ZnSiS_4_, and a proposed Li_6_ZnS_4_ were examined (Figure 2b). No evidence was observed for the formation of new phases in the solid solution Li_4–2*x*_Zn_*x*_SiS_4_ with end members Li_4_SiS_4_ and Li_2_ZnSiS_4_ being the only observed phases (Figures 2b (purple line) and S1). Reactions along the Li_2+4*x*_ZnSi_1–*x*_S_4_ tie line yielded mixtures of Li_2_ZnSiS_4_, Li_4_SiS_4_, Li_2_S, and ZnS (Figures 2b (green line) and S2). A first firing at 973 K for 24 h at the composition *x* = 0.2 along the Li_4+2*x*_Zn_*x*_Si_1–*x*_S_4_ tie line (Figure 2b blue line) produced a set of reflections in the PXRD pattern that could not be assigned to any known phase with the *x* = 0.4 composition also yielding these peaks but with lower intensity (Figure S3). A second additional firing at 973 K for 24 h was critical to increase the amount of this phase at other compositions with the reflections of the new phase observed strongly in Li_4.534_Zn_0.267_Si_0.733_S_4_ (*x* = 0.267) after the additional firing. The synthesis temperature was found to be critical to produce this new phase with the formation observed only in the reactions carried out at 973 K (Figure S4). Below this temperature, a mixture of Li_2_S and Li_4_SiS_4_ is obtained instead. A phase pure powder sample was obtained at the composition *x* = 0.33 (Li_4.67_Zn_0.33_S_0.67_S_4_), where neither Li_2_S nor Li_4_SiS_4_ (impurities observed in local compositions) could be detected by laboratory PXRD measurements. The optimal reaction conditions for the formation of Li_4.67_Zn_0.33_S_0.67_S_4_ are firing at 973 K for 24 h twice using heating and cooling rates of 5 K min^–1^ with the powder ground using a pestle and mortar between the two firings.

The nominal composition of *x* = 0.33 in Li_4+2*x*_Zn_*x*_Si_1–*x*_S_4_ can be written as Li_7_Zn_0.5_SiS_6_, indicating that the new phase is related to argyrodite, a well-known family of superionic conductors. The bulk composition of Li_7.07(2)_Zn_0.472(2)_Si_1.068(6)_S_6.00(6)_ measured by ICP-AES (Table S1) agrees well with the expected composition of Li_7_Zn_0.5_SiS_6_ and confirms that there is no loss of reagents through the reaction with the carbon-coated quartz ampule. The structure of this new phase was determined through the analysis of synchrotron X-ray diffraction (SXRD) and neutron powder diffraction data (NPD) described in detail in the following sections. All of the reflections observed for new Li_7_Zn_0.5_SiS_6_ can be indexed to a tetragonal unit cell at room temperature with lattice parameters *a* = *b* ≈ 21 Å and *c* ≈ 10 Å with systematic absences that are consistent with the *I* – – – diffraction symbol (Figure S5). High-resolution SXRD data were collected on Li_7_Zn_0.5_SiS_6_ powder in the temperature range of 298–448 K in 25 K steps. Above 423 K, the convergence of some peaks and disappearance of many small peaks associated with the tetragonal lattice indicate a phase transition to a higher-symmetry structure takes place (Figures S5 (inset) and S6). This transition, observed by powder diffraction, is consistent with differential scanning calorimetry (DSC) data, which show a single endothermic and exothermic thermal event at 411.1(5) K observed on heating and cooling, respectively, highlighting that the transition is reversible (Figure 2c). The peaks in the powder pattern of Li_7_Zn_0.5_SiS_6_ measured at >423 K can be indexed to a cubic unit cell with *a* = 10.04444(3) Å and space group symmetry *F*43*m* (Figure S5), which is similar to those observed for argyrodite materials. It is not possible to stabilize the high-temperature *F*43*m* structure of Li_7_Zn_0.5_SiS_6_ to room temperature through quenching. This yields tetragonal (*I*4) Li_7_Zn_0.5_SiS_6_ and Li_4_SiS_4_ as a secondary phase (Figure S7).

### 3.2 Structure Determination

#### 3.2.1 Determination of Li_7_Zn_0.5_SiS_6_ High-Temperature Structure

**Figure 3:**
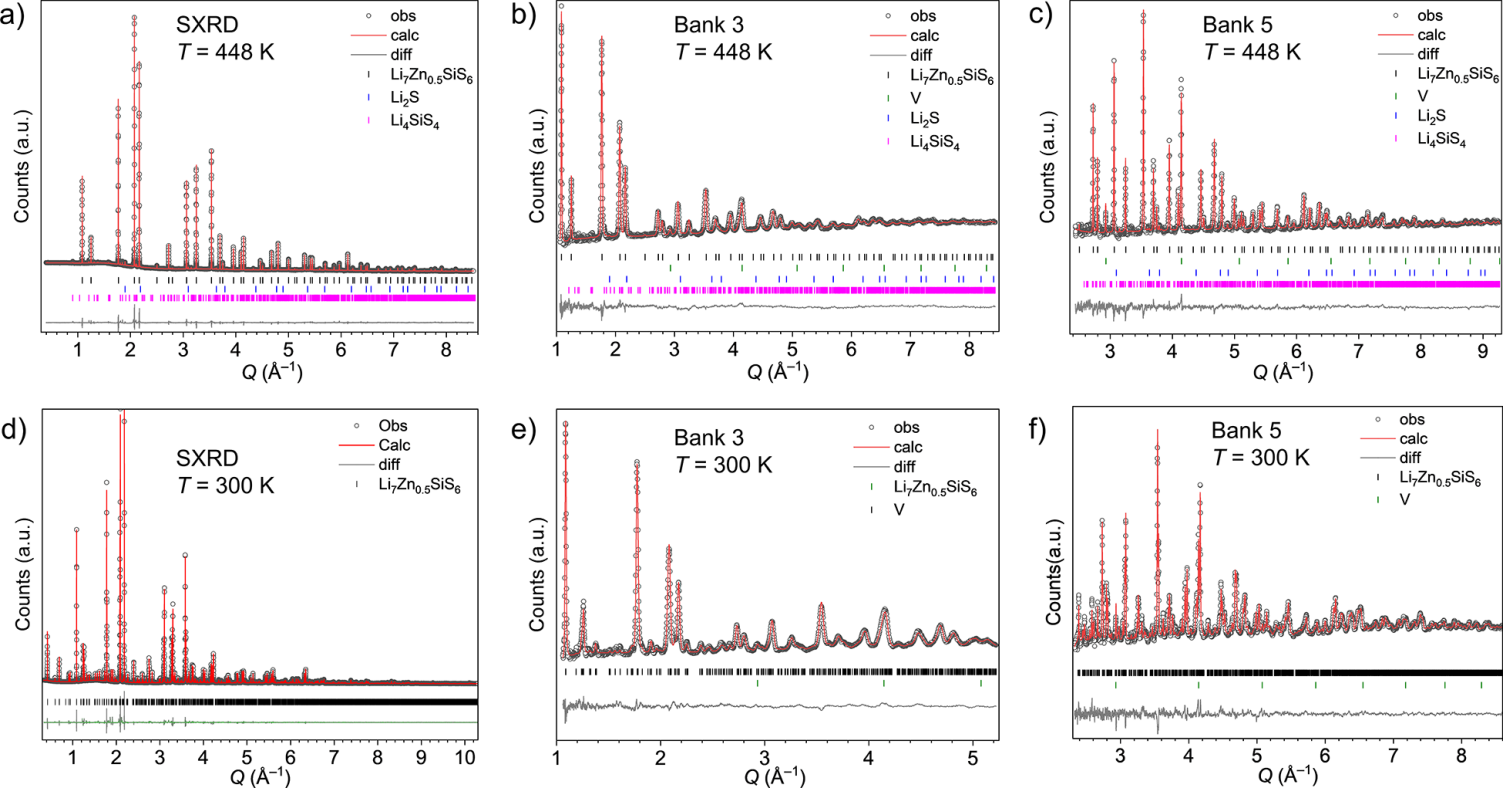
(a–c) Rietveld refinement against SXRD (I11, Diamond Light Source) and NPD data from Bank 3 and Bank 5 (Polaris, ISIS) at 448 K of the high-temperature structure of Li_7_Zn_0.5_SiS_6_ with *F*43*m* symmetry. (d–f) Rietveld refinement against SXRD and NPD Bank 3 and Bank 5 data at 300 K using the room-temperature structural model of Li_7_Zn_0.5_SiS_6_ with *I*4 symmetry. Traces *I*_obs_ (black circles), *I*_calc_ (red line), and *I*_obs_ – *I*_calc_ (gray line) and Bragg reflections are shown (black tick marks for Li_7_Zn_0.5_SiS_6_, green for V metal, blue for Li_2_S, pink for Li_4_SiS_4_). The high-temperature refinement has *R*_wp_ = 3.58% and χ^2^ = 38.2 for 109 refined parameters, and the room-temperature refinement has *R*_wp_ = 5.60% and χ^2^ = 14.8 for 245 refined parameters.

The high-temperature *F*43*m* structure of Li_7_Zn_0.5_SiS_6_ was obtained from the combined refinement of SXRD and NPD data, fitted using TOPAS Academic.^22^ The structure of Li_6_PS_5_Br (space group *F*43*m* with approximate lattice parameter of *a* ≈ 10.04 Å) was used as the starting point^9^ with P substituted for Si on the 4*b* Wyckoff site and Br substituted for S on the anion positions. The lattice parameter, background, and peak shapes were refined via the Le Bail method and then fixed throughout until the final refinement. Two Li sites (48*h* T5 and 24*g* T5a) were present in the starting model. Compared against the nominal composition of Li_7_Zn_0.5_SiS_6_, this structure is missing 1.5 cations (1 Li^+^ and 0.5 Zn^2+^). Three additional tetrahedral Li sites were suggested from a Fourier difference map generated using NPD data from Bank 3 of Polaris with the initial model and profile parameters from the Le Bail fit. The Li site occupancies were initially set such that the total Li content within the model was equal to 7.5 from five sites (four tetrahedra and one trigonal planar). The four tetrahedral sites were duplicated for Zn and set to an initial occupancy of zero. The site distribution of Li/Zn within the structural model was then refined using the automatic simulated annealing algorithm in TOPAS, refining for 100 000 cycles. During the simulated annealing, only the occupancies of the Li/Zn sites were allowed to vary, and the refinement was subject to a global composition restraint set by the nominal composition (Li_7_Zn_0.5_SiS_6_). The simulated annealing resulted in Zn located on the 48*h* (T5) site only with Li occupying the 48*h* (T5), 24*g* (T5a), 48*h* (T2), and 16*e* (T4) sites. The fifth candidate tetrahedral site was refined to an occupancy of zero, so it was removed from the refinement. These results from simulated annealing were then used as the basis for a Rietveld refinement in which all structural parameters and profile parameters were refined. During this final refinement, the results of which are shown in Figures 3a–c and S9a–c and given in Table 1, the global composition restraint was still used, and soft restraints were applied to the Si–S and Li–S distances of 2.1(1) and to 2.5(1) Å, respectively. These values were based on the averages if both distances were freely refined, while corresponding to sensible Si–S and Li–S distances as found in other argyrodites.^13^ The final refinement resulted in the unit cell parameter *a* = 10.04444(3) Å and composition of Li_7.00(12)_Zn_0.497(6)_SiS_6_, which is in good agreement with the composition of Li_7.07(2)_Zn_0.472(2)_Si_1.068(6)_S_6.00(6)_ determined analytically from ICP-AES analysis (Table S1). Though many Cu and Ag argyrodites exist with high mobile cation content (e.g., Cu_8_GeS_6_ and Ag_8_SnSe_6_),^3,4^ comparatively few Li argyrodites with such a high occupancy of the mobile cation sites exist.

**Table 1:** High-Temperature Structure of Li_7_Zn_0.5_SiS_6_ at 448 K in Space Group *F*43*m* with Refined Unit Cell Parameter *a* = 10.04444(3) Å and Total Refined Composition of Li_7.00(12)_Zn_0.497(6)_SiS_6_

site	Wyckoff position	*x*	*y*	*z*	*U*_iso_ (Å^2^)	occ.
S1	4*c*	0.25	0.25	0.25	0.0336(4)	1
S1	16*e*	0.12155(6)	0.12155(6)	0.62155(6)	0.03266(17)	1
S3	4*a*	0	0.5	0.5	0.0569(5)	1
Si1	4*b*	0	0	0.5	0.0229(3)	1
Li1	48*h*	0.1833(3)	0.3167(3)	0.4843(4)	0.0567(12)	0.405(8)
Zn1	48*h*	0.1833(3)	0.3167(3)	0.4843(4)	0.0567(12)	0.0414(5)
Li2	24*g*	0.25	0.25	0.494(6)	0.0567(12)	0.099(7)
Li3	16*e*	0.1280(13)	0.3720(13)	0.6280(13)	0.0567(12)	0.243(6)
Li4	48*h*	0.074(3)	0.286(5)	0.426(3)	0.0567(12)	0.048(5)

#### 3.2.2 Determination of Li_7_Zn_0.5_SiS_6_ Room-Temperature Structure

In principle, it should be possible to solve the room-temperature ordering of Li_7_Zn_0.5_SiS_6_ by creating the corresponding supercell of the high-temperature structure using ISODISTORT^23,24^ and then performing a Rietveld refinement. However, this generates a supercell containing 77 unique Li/Zn sites, 3 Si sites, and 15 S sites (a total of 95 sites). Consequently, solving the room-temperature structure via this route was impractical, as preliminary refinement attempts immediately indicate that the majority of the potential Li/Zn sites are not occupied at room temperature; therefore, the determination of which sites were occupied by Li and Zn starting from all possible sites would be less efficient compared to resolving through the method described below.

To solve the room-temperature structure of Li_7_Zn_0.5_SiS_6_, the unit cell was first indexed using the autoindexing function in GSAS-II with the SXRD data,^25^ which resulted in the unit cell indexed as tetragonal with approximate lattice parameters *a* = *b* ≈ 21 Å and *c* ≈ 10 Å and systematic absences which are consistent with the *I* – – – diffraction symbol. This approximate unit cell was then used as the basis for a Le Bail refinement in Jana2006.^26^ For the Le Bail fit, the space group was set to *P*1 and the background, peak profile, the six unit cell parameters, and zero error were then refined. The “make space group test” function within Jana2006 was used to suggest possible space groups with a tolerance of 0.02 Å on the *a*, *b*, and *c* unit cell parameters and 0.2° on the unit cell angles and was combined with the HKL table from the Le Bail fit to suggest possible space groups, which typically returns the highest symmetry space group from the best fitting diffraction symbol. The best achieved fit was to the space group *I*4*m*2, consistent with *I* – – −. The initial structure was solved using SUPERFLIP^27,28^ as implemented in Jana2006. The best solutions from this method can often be achieved by starting with the lowest symmetry space group allowed by the *I* – – – diffraction symbol. As such, the structure solution was started in space group *I*4, and SUPERFLIP with its own symmetry determination is able to suggest higher symmetry solutions, where appropriate, although none that yielded a solution with an improved fitting parameter were found.

The solution from SUPERFLIP using the results from a Le Bail fit in *I*4 symmetry against SXRD data yielded a structure containing 15× S, 3× Si, and 12× mixed Li/Zn sites and retained the *I*4 symmetry. This model was refined using the Rietveld method in Jana2006 against the SXRD data, allowing the occupancies of the mixed Li/Zn sites to be refined while restricting each total site occupancy to one, resulting in a structural model with an approximate composition of Li_4.55_Zn_0.5_SiS_6_. This indicated a significant amount of Li that was unaccounted for in the structure. In this model, no further sites could be located using Fourier difference mapping against the SXRD data, and due to the large difference between the model and nominal composition of Li_7_Zn_0.5_SiS_6_, it was concluded that the additional Li was likely distributed across several partially occupied sites.

To locate further Li sites within the structure, the structure derived above was refined in TOPAS academic to create an equivalent starting point and NPD data were now included in the refinement. Computing Fourier difference maps against the NPD data from Bank 3 of Polaris did not locate any additional Li sites. We repeated the following process until no new sites could be located: an additional Li/Zn site was included in the structure with randomly generated (within TOPAS) fractional coordinates with the total occupancy of the site restricted to one; this updated model is then refined using the Rietveld method for at least 10 000 refinement cycles, and each time the refinement converged, the coordinates of the new site were re-randomized (the rest of the structure and profile parameters continue to be refined as normal). Then, the solution with the lowest *R*_wp_ fit parameter is carried forward. This process resulted in the location of an additional four Li sites, bringing the approximate composition to Li_7_Zn_0.5_SiS_6_ in line with the nominal composition.

At this stage, the restriction of all Li/Zn sites having a total occupancy of one led to a number of nonphysical M–M distances (M = Li and Zn) of approximately 1.6 Å between sites within the model. This was resolved by restricting the total occupancies of these neighboring positions (sites with short M–M distances) to a maximum of one. The original restriction that maintained Li/Zn site occupancies to equate to one was changed such that the occupancy contribution on any given Li/Zn site could not fall below zero. With these restrictions in place, simulated annealing was used in TOPAS for at least 100 000 refinement cycles using its automatic temperature regime, allowing only the occupancies of the Li/Zn sites to be refined. After each simulated annealing run, further Li sites were trialed in the structure as outlined previously. With the addition of each new Li/Zn site, the constraints on the occupancy of the neighboring Li/Zn sites were updated and the simulated annealing stage repeated. This process introduced an additional five Li/Zn sites into the structure at which point the composition of the model was stable and no new sites could be located (i.e., no new Li/Zn sites could be introduced that had total refined occupancies greater than one estimated standard deviation above zero). The refined composition of the model at this stage was Li_6.85(10)_ Zn_0.470(6)_ SiS_6_.

To achieve the final refinement model, distance restraints were used for the Si–S and Li/Zn–S bonds. Si–S distances were constrained softly to 2.1(±0.1) Å, while for Li/Zn–S bonds, the distance was constrained to 2.5(±0.1) Å. These values were based on the averages if distances were freely refined, while simultaneously corresponding to sensible Si–S and Li–S distances as found in other argyrodites.^13^ Additionally, a soft global composition restraint, set to the nominal composition of Li_7_Zn_0.5_SiS_6_, was used in the model. The final refined model has the composition Li_7.00(8)_Zn_0.480(5)_SiS_6_ in excellent agreement with the composition determined through ICP analysis (Li_7.07(2)_Zn_0.472(2)_Si_1.068(6)_S_6.00(6)_) with lattice parameters *a* = *b* = 21.15065(2) Å and *c* = 10.05640(15) Å and a global *R*_wp_ of 5.6% and χ^2^ of 14.8 for 245 parameters. For reference, the equivalent Le Bail fit of the data used in the final refinement has a *R*_wp_ of 5.1% and χ^2^ of 12. Attempts to identify additional symmetry elements in the final model using FINDSYM^29^ were unsuccessful, confirming the *I*4 space group. The results from the final structure solution are shown in Figures 3d–f and S9d–f and given in Table S2. The room-temperature structure of Li_7_Zn_0.5_SiS_6_ contains 40 unique atomic sites, 22× Li sites, of which 6 contain Zn, 3× Si sites, and 15× S sites, and are discussed in detail below.

### 3.3 Structure Description

#### 3.3.1 Description of High-Temperature Li_7_Zn_0.5_SiS_6_ Structure

**Figure 4:**
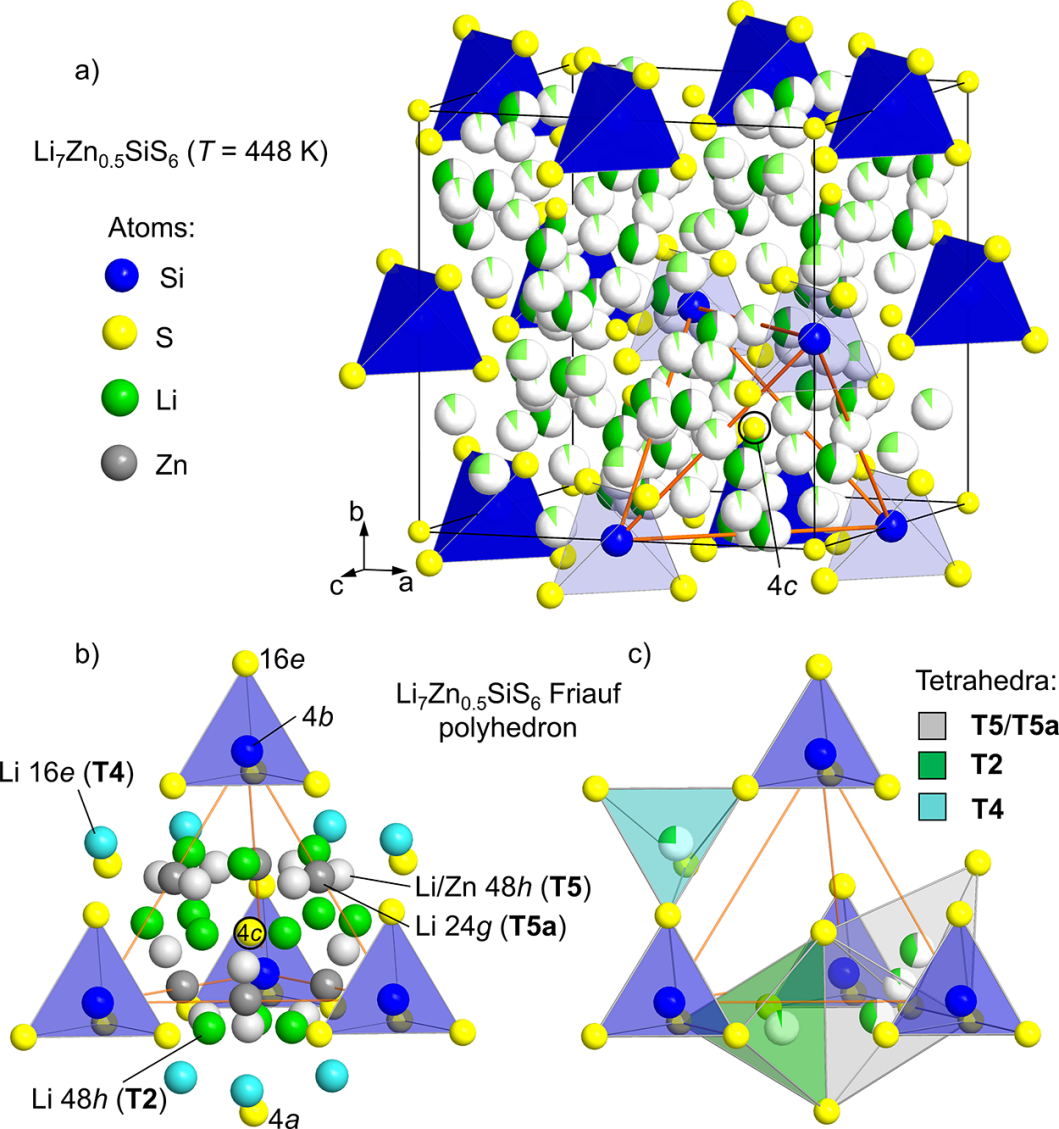
(a) Unit cell of *F*43*m* Li_7_Zn_0.5_SiS_6_ and (b) representation of the Li sublattice within a Friauf polyhedron. The Friauf polyhedron is formed from four SiS_4_ tetrahedra (blue) centered around the S^2–^ anion on the 4*c* Wyckoff position (circled in (a) and (b)). The orange lines in (a) show the Friauf polyhedron within the unit cell. For ease of viewing, the Li sites in (b) are represented as fully occupied spheres and type by color: light gray, T5 site (0.405(8) Li and 0.0414(5) Zn); dark gray, T5a site (0.099(7) Li); green, T2 site (0.048(5) Li); light blue, T4 site (0.243(6) Li). Li occupies all four sites: 48*h* (T5), 24*g* (T5a), 48*h* (T2), and 16*e* (T4). Zn occupies the 48*h* (T5) site only. A single tetrahedron for each type of Li environment is shown in (c): gray, T5 and T5a; green, T2; light blue, T4. For clarity, all atoms outside of these tetrahedra have been removed. The T2 and T4 sites are typically vacant in most Li-containing *F*43*m* argyrodite structures, and the occupancy of one of the T2 or T4 sites is observed in only a small number of materials.^31−34^

Li_7_Zn_0.5_SiS_6_ adopts the typical cubic *F*43*m* argyrodite structure at high temperatures (>423 K), comparable to that of Li_6_PS_5_Br,^9^ with lattice parameter *a* = 10.04444(3) Å obtained from the Rietveld refinement against SXRD and NPD data (Figures 3a–c and 4a). Si^4+^ occupies the 4*b* Wyckoff site and is tetrahedrally coordinated by S^2–^ anions on the 16*e* site, and these SiS_4_^4–^ polyanions are packed in a cubic close-packed arrangement along [111]. S^2–^ anions occupy half of the tetrahedral and all of the octahedral voids between these SiS_4_^4–^ polyanions on the 4*c* and 4*a* positions, respectively. Considering the anion sublattice from the perspective of antiperovskite, the SiS_4_^4–^ polyanions and S^2–^ anions on the 4*a* position occupy the anti-A site in a rocksalt ordered fashion, and the S^2–^ anions on the 4*c* position occupy half of the octahedral anti-B site also in a rock salt ordered manner where half of the octahedra are empty such that the chemical formula of Li_7_Zn_0.5_SiS_6_ argyrodite can also be written as [((SiS_4_)_0.5_S_0.5_)(S_0.5_□_0.5_)Li_3_Zn_0.25_]_2_.^6^

Li^+^ cations are dynamically disordered across four distinct crystallographic sites in the high-temperature structure of Li_7_Zn_0.5_SiS_6_ and can be represented by viewing Friauf polyhedra formed from the four SiS_4_^4–^ polyanions that are centered around the 4*c* S^2–^ position (Figure 4). The 48*h* (T5) and 24*g* (T5a) positions are occupied with 0.405(8) and 0.099(7) Li, respectively, accounting for most of the Li. Despite having a comparable ionic radius to Li^+^ (tetrahedral *r*_Li_^+^ = 0.59 Å and *r*_Zn_^2+^ = 0.6 Å),^30^ Zn^2+^ occupies only one site in the high-temperature structure, which is the 48*h* (T5) site with an occupancy of 0.0414(5) Zn, giving an overall cation occupancy of 0.99(2) across the T5 and T5a sites (Figure 4b,c). The remaining Li occupies the 16*e* (T4) and 48*h* (T2) positions with occupancies of 0.243(6) and 0.048(5), respectively (Figure 4b,c). These additional (T4 and T2) positions are sites that are less frequently occupied than the T5 and T5a sites in most Li-containing argyrodites; however, they have been observed in several other systems such as Li_6+*x*_Sb_1–*x*_Sn_*x*_S_5_I,^14^ Li_6.15_Al_0.15_Si_1.35_S_5.4_O_0.6_,^31^ Li_6.15_Al_0.15_Si_1.35_S_5.734_O_0.266_,^32^ and Li_6_PS_5_X (X = Cl and Br) from the analysis of high-resolution neutron diffraction data,^33^ and the simultaneous occupation of T5, T5a, T2, and T4 sites was very recently reported in Li_6.6_P_0.4_Ge_0.6_S_5_I.^34^ The 48*h* (T5) tetrahedra share two corners with the SiS_4_^4–^ polyanions with the other two corners formed by the S^2–^ 4*c* and 4*a* positions. A small amount of Li^+^ (0.099(7)) is delocalized onto the 24*g* (T5a) site, which lies in between the 48*h* (T5) positions, yielding a trigonal planar environment (Figure 4b,c). The 48*h* (T2) position acts as an interstitial site between adjacent 48*h* (T5) positions forming tetrahedra that face share with T5 sites and share edges with the SiS_4_^4–^ polyanions. The 16*e* (T4) sites also share faces with the adjacent 48*h* (T5) tetrahedra and share edges with the 48*h* (T2) tetrahedra. As such, all four faces of the 48*h* (T5) tetrahedral environment in the average high-temperature structure of Li_7_Zn_0.5_SiS_6_ are shared with other tetrahedral environments (one 48*h* T5, two 48*h* T2, one 16*e* T4), yielding a significantly delocalized distribution of Li^+^, such as those observed recently in Li_6_PS_5_X (X = Cl, Br), Li_6.15_Al_0.15_Si_1.35_S_5.4_O_0.6_, Li_6.15_Al_0.15_Si_1.35_S_5.734_O_0.266_, and Li_6.6_P_0.4_Ge_0.6_S_5_I.^31−34^ The T2 site is located within the Li cages, which are formed by the T5 and T5a Li^+^ positions centered around the 4*d* S^2–^/X^–^ site, whereas the T4 site is located between these cages; both the T2 and T4 sites are known to play an important role in the formation of ionic diffusion pathways throughout the argyrodite structure, which will be discussed in Section 3.4.

#### 3.3.2 Description of Room-Temperature Li_7_Zn_0.5_SiS_6_ Structure

**Figure 5:**
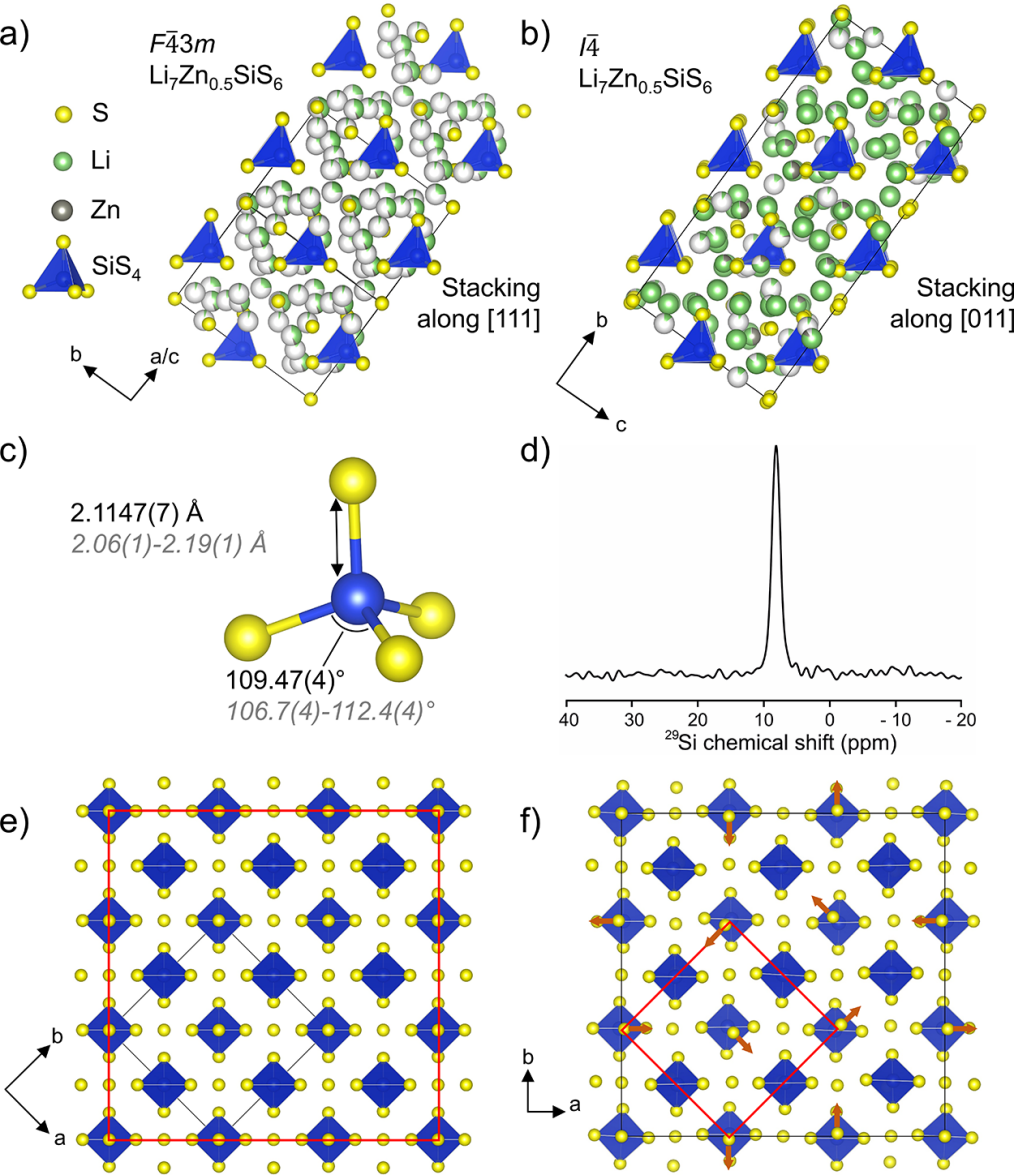
(a) High-temperature (*T* = 448 K) *F*43*m* and (b) room-temperature (*T* = 300 K) *I*4 structures of Li_7_Zn_0.5_SiS_6_ showing cubic close-packed layers of SiS_4_^4–^ polyanions stacked along the [111] and [011] directions, respectively. Black lines represent the unit cell edges. Atom colors: Li, green; Zn, gray; S, yellow; SiS_4_ tetrahedra, blue. (c) SiS_4_ tetrahedral environment with a range of bond distances and angles listed in black and italicized gray for high-temperature *F*43*m* and room-temperature *I*4 structures of Li_7_Zn_0.5_SiS_6_, respectively. (d) ^29^Si MAS NMR spectrum of Li_7_Zn_0.5_SiS_6_ measured at 300 K. A larger spectral width covering the Q^n^ region is shown in Figure S12. The anion sublattice of the (e) high-temperature *F*43*m* structure and (f) room-temperature *I*4 structure of Li_7_Zn_0.5_SiS_6_. The black lines represent the unit cell edges, and the red lines highlight the relationship between the two structures. Small orange arrows in (f) emphasize the displacements of the two 8*g* positions (equivalent to the 4*a* position in the high-temperature *F*43*m* structure) away from the center of the octahedral voids formed from the SiS_4_^4–^ cubic close-packing.

Below 411 K, Li_7_Zn_0.5_SiS_6_ orders in a tetragonal structure with unit cell parameters of *a* = *b* = 21.15065(2) Å and *c* = 10.05640(15) Å with *I*4 symmetry, which is related to the high-temperature *F*43*m* unit cell through the relationship , where *a*_t_ and *a*_c_ are the unit cell parameters of the tetragonal and cubic structures, respectively. There are no previous reports of a tetragonal argyrodite, which arises here from the combination of Li^+^ and a small amount Zn^2+^ (6.7% of the total number of cations) on the mobile cation sites. The anion sublattice (S^2–^ and SiS_4_^4–^) of the Li_7_Zn_0.5_SiS_6_ room-temperature *I*4 structure has identical cubic close-packing of the SiS_4_^4–^ polyanions as in the *F*43*m* structure, along [011] in the *I*4 unit cell (Figure 5a,b), and the two distinct lone S^2–^ anions occupy half of the tetrahedral and all of the octahedral voids on 8*g*, 2*a*, and 2*d* Wyckoff positions (Table S2). There are three distinct Si^4+^ positions (one 2*b* and two 8*g*), which give three types of SiS_4_^4–^ tetrahedra. The tetrahedra in the room-temperature *I*4 structure are distorted slightly from the regular tetrahedra of the high-temperature *F*43*m* structure with Si–S distances in the range of 2.06(1)–2.19(1) Å and S–Si–S angles of 106.7(4)–112.4(4)° compared against 2.1147(7) Å and 109.47(4)°, respectively (Figure 5c, Tables S3–S6). Though slightly irregular, these distances and angles of the tetrahedra are similar enough such that different environments are not spectroscopically resolved by ^29^Si MAS NMR (Figure 5d). A single sharp resonance characteristic of SiS_4_ tetrahedra^35−37^ is observed at 8 ppm in the NMR spectrum of Li_7_Zn_0.5_SiS_6_, highlighting the similarity of the SiS_4_^4–^ tetrahedra in the room-temperature *I*4 structure. This is also consistent with the measured Raman spectrum of Li_7_Zn_0.5_SiS_6_ (Figure S10), which shows modes characteristic of SiS_4_^4–^ tetrahedra at frequencies comparable to those observed for Cu_8_MCh_6_ (M = Si, Ge and Ch = S, Se).^38^ The S^2–^ anions that occupy the 4*a* and 4*c* positions in the high-temperature *F*43*m* structure occupy the 2*a* and two 8*g* and the 2*d* and two 8*g* positions, respectively, in the room-temperature *I*4 structure. The 2*d* and two 8*g* positions (equivalent to the 4*c* position in the high-temperature *F*43*m* structure) occupy the center of half of the tetrahedral holes formed by the cubic closed-packed SiS_4_^4–^ sublattice. The 2*a* and two 8*g* positions (equivalent to the 4*a* position in the high-temperature *F*43*m* structure) occupy the octahedral voids formed from the SiS_4_^4–^ cubic close-packing; however, the S^2–^ anions on the two 8*g* positions are displaced away from the octahedral center in the room-temperature *I*4 structure by 0.199(8) and 0.377(9) Å compared to the high-temperature *F*43*m* structure (Figures 5e,f and S11). The sulfide anions are displaced toward the neighboring sites, which are occupied by both Li and Zn and away from the sites partially occupied with Li, likely increasing valence and bonding for the former sites (Figure S11).

**Figure 6:**
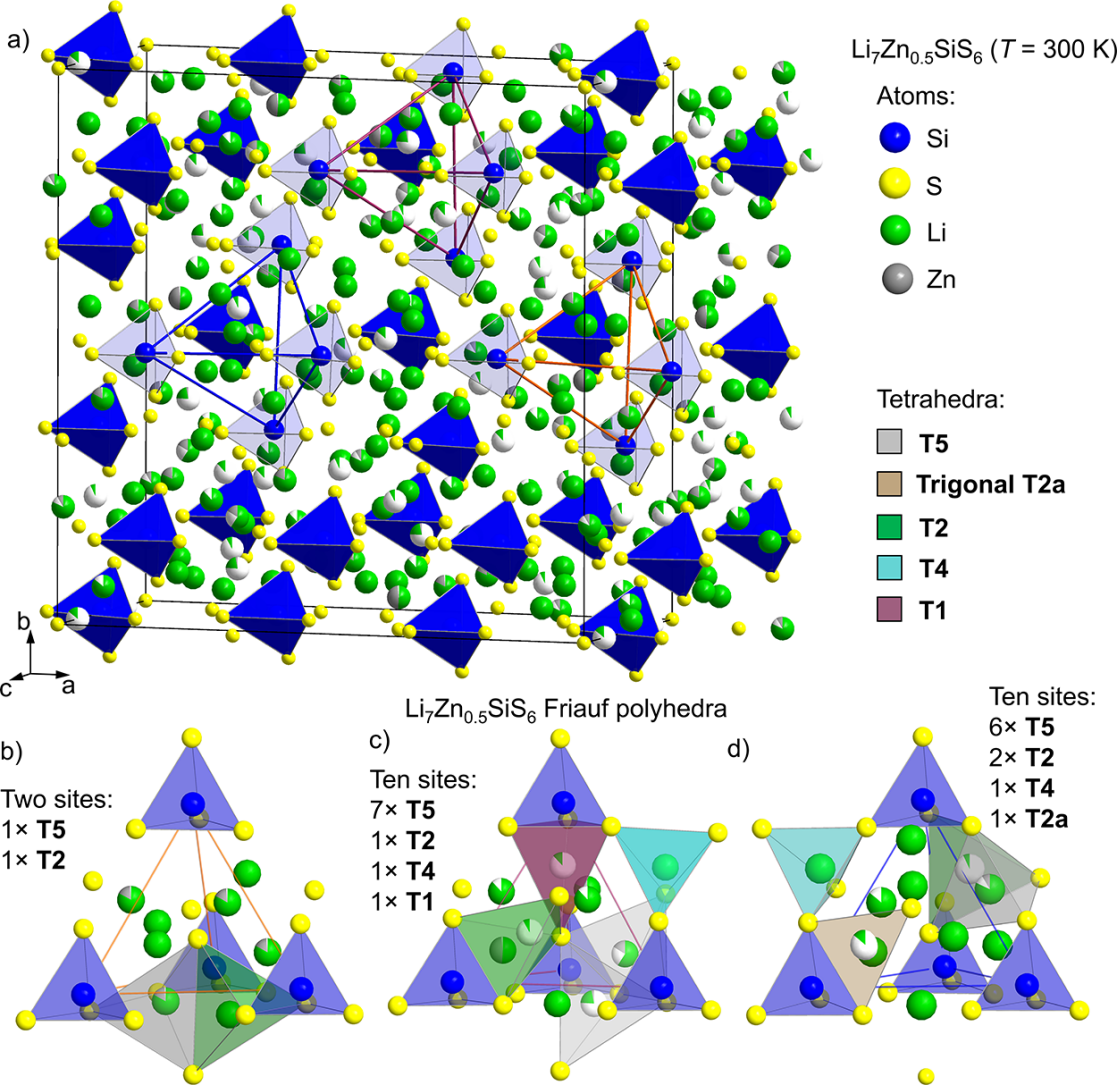
(a) Unit cell of room-temperature *I*4 Li_7_Zn_0.5_SiS_6_ and (b–d) representation of the Li sublattice within the three types of Friauf polyhedra. The Friauf polyhedra are formed from four SiS_4_ tetrahedra (blue) around a central S^2–^ anion on the 2*d* and two 8*g* Wyckoff positions (corresponding to the 4*c* site in the high-temperature *F*43*m* structure). The orange, purple, and blue lines in (a) show the Friauf polyhedra within the unit cell. In total, there are 22 distinct Li positions in *I*4 Li_7_Zn_0.5_SiS_6_, and all are shown in (b–d). Tetrahedra for different types of Li sites are shown: gray, T5 and T5a; green, T2; light blue, T4; purple, T1; orange, new trigonal planar T2a position. The T1 and new trigonal planar T2a positions (which occupy the shared face between two T2 neighboring tetrahedra) are not occupied in high-temperature *F*43*m* Li_7_Zn_0.5_SiS_6_ and are shown in (c) and (d), respectively.

In the room-temperature *I*4 structure of Li_7_Zn_0.5_SiS_6_, there are 22 crystallographically distinct Li positions (Table S2): 14 × T5, 4 × T2, 2 × T4, 1 × T1, and 1 trigonal planar (denoted as T2a) position, which has not been observed in argyrodites before. The latter two positions (T1 and T2a) are not occupied in the high-temperature *F*43*m* structure. These positions are represented in three separate Friauf polyhedra shown in Figure 6, which are formed from four SiS_4_^4–^ polyanions that center around S^2–^ anions on the 2*d* and two 8*g* positions (4*c* in the high-temperature *F*43*m* structure). Zn occupies the 48-fold T5 site at high temperature in *F*43*m* Li_7_Zn_0.5_SiS_6_; therefore, any of the T5 sites in *I*4 Li_7_Zn_0.5_SiS_6_ can be occupied by Zn. However, only a subset of the T5 sites in the room-temperature *I*4 structure are occupied by Zn. Six of the 14 T5 sites (48*h* position in high-temperature *F*43*m* structure) are occupied by both Li^+^ and Zn^2+^, the smallest and largest Zn occupancies being 0.031(5) and 0.473(4), respectively. These six T5 sites are the only positions that are occupied by Zn in the room-temperature *I*4 structure of Li_7_Zn_0.5_SiS_6_. Almost all of the T5 sites have occupancies higher than 0.85; five T5 sites are fully occupied by Li, and a further seven have occupancies higher than 0.86(4). The two remaining T5 sites (1× 0.162(5)Zn/0.59(6)Li and 1× 0.031(5)Zn/0.21(6)Li) occupy face-sharing tetrahedra, giving a combined occupancy of 0.99(9) across both sites, analogous to the disordered environments in the high-temperature *F*43*m* structure (Figure 6c). The large number of T5 sites occupied in Li_7_Zn_0.5_SiS_6_ is consistent with other ordered Li-rich argyrodites, such as Li_7_PCh_6_ (Ch = S, Se), in which Li predominantly occupies tetrahedral T5 sites (six T5 sites and one T5a site).^7^

None of the trigonal planar 24*g* (T5a) positions occupied in the high-temperature *F*43*m* structure are occupied at room temperature, reflecting the preference for a higher (tetrahedral) coordination environment for Li^+^ in Li_7_Zn_0.5_SiS_6_. This is distinct from the low temperature (*Cc*) structures of Li_6_AsS_5_I and Li_6_PS_5_I and the *F*43*m* structure of Li_6_PO_5_Br, all of which contain trigonal planar T5a positions fully occupied by Li.^7,10^ This is not exclusive to anion-ordered argyrodites, as fully occupied trigonal planar T5a environments are also observed in Li_7_PCh_6_ (Ch = S, Se).^7^

Two of the four T2 sites in Li_7_Zn_0.5_SiS_6_ are fully occupied with Li at room temperature and share edges with their neighboring Li sites. The remaining two T2 sites have low occupancies (<0.09) and share faces with occupied neighboring T5 sites. The two T4 sites are both fully occupied with Li at room temperature (Figure 6c,d). The occupancy of a single T1 site at room temperature in Li_7_Zn_0.5_SiS_6_ (Figure 6c) is surprising as this position is not usually occupied in other argyrodites. Through consideration of the tetrahedral holes available for occupancy by Li in the argyrodite structure, this site is the least favorable as it shares a common face with the SiS_4_^4–^ tetrahedra, which would result in significant repulsion from the nearby Si^4+^ cation located only 2.1(2) Å away.^7^ This is likely the reason the T1 site in Li_7_Zn_0.5_SiS_6_ is occupied with only 0.12(3) Li, and the position is not occupied in the high-temperature *F*43*m* structure. Finally, there is one Li site that adopts trigonal planar geometry in Li_7_Zn_0.5_SiS_6_ at room temperature (4*e* Wyckoff position), different from the commonly occupied T5a site, is not observed in other argyrodites, and like the T1 position is not occupied in the high-temperature structure. This is the trigonal face that is shared between two neighboring T2 tetrahedra, and as such, this new site is denoted as T2a. This position shares edges with one SiS_4_^4–^ tetrahedra and two T5 Li tetrahedra, yielding a short Li–Li of 1.82(7) Å (Figure 6d). The combined occupancy of these positions sums to unity.

^6^Li MAS NMR was utilized to obtain insights into the local structure and ordering of Li_7_Zn_0.5_SiS_6_ at room temperature. The ^6^Li MAS NMR spectrum recorded at room temperature (Figure S12b) displays a single narrow resonance at 1.65 ppm, which agrees well with all (but one) of the lithium atoms occupying tetrahedral sites. The low occupancy of the remaining single lithium in the T2a trigonal planar position among the 21 other Li sites likely limits the clear observation of this local environment in the ^6^Li spectrum. It is also quite likely that the ^6^Li MAS NMR spectrum is motionally averaged over all crystallographic sites due to fast Li ion hopping (see ^7^Li NMR data below) and prevents spectral resolution of the two different Li local environments.

**Figure 7:**
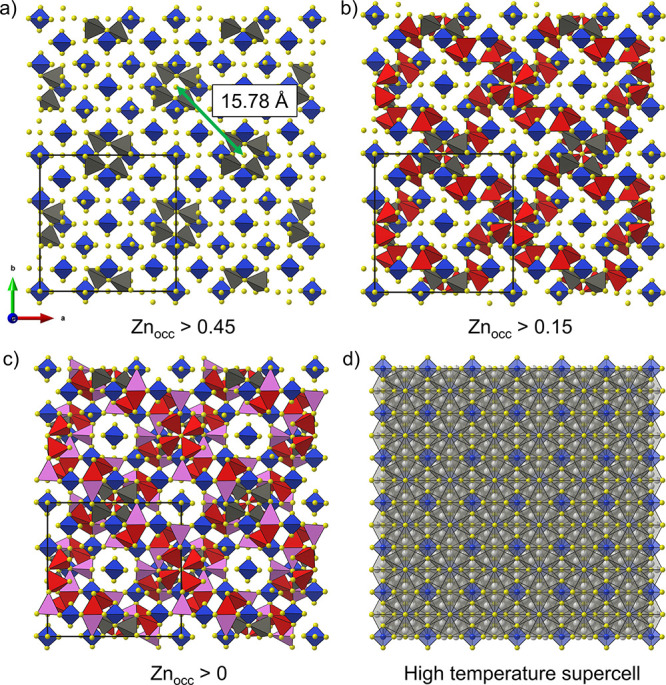
A 2 × 2 × 1 super cell of Li_7_Zn_0.5_SiS_6_ with Li sites omitted, viewed along the [001] direction. Zn sites are shown with a decreasing occupancy threshold between panels: (a) Zn occupancy > 0.45 with one unique site (gray tetrahedra), (b) Zn occupancy > 0.15 with three additional unique sites (red tetrahedra), and (c) Zn occupancy > 0 with two additional unique sites (pink tetrahedra). The equivalent supercell of the (d) high-temperature *F*43*m* structure highlights Zn distributed throughout the average structure occupying the 48*h* T5 position in contrast to the ordering observed at room temperature.

The addition of a relatively small amount of Zn^2+^ (Li/Zn ratio is 14:1 in Li_7_Zn_0.5_SiS_6_) results in the ordering observed below 411 K in the tetragonal *I*4 Li_7_Zn_0.5_SiS_6_ structure. Argyrodites that contain more than one metal on the mobile cation sites (i.e., sharing the site with Li^+^) are rare but are not unheard of. Both Al^3+^ and Si^4+^ occupy 48*h* and 16*e* positions alongside Li^+^ in Li_6.15_Al_0.15_Si_1.35_S_5.4_O_0.6_ and Li_6.15_Al_0.15_Si_1.35_S_5.734_O_0.266_,^31,32^ while Li_3.5_Ge_1.5_P_0.5_S_6_ has Ge^4+^ present on both the 48*h* (shared with Li^+^) and 4*b* (shared with P^5+^) positions.^39^ These three materials retain the *F*43*m* symmetry typical of argyrodites, and the additional cations (Al^3+^, Si^4+^, and Ge^4+^) present on the Li sites in these materials do not exhibit ordering of any kind. This is distinct from Li_7_Zn_0.5_SiS_6_ in which a unique ordering pattern occurs in the Zn distribution that stabilizes the room-temperature *I*4 structure, explaining why only 6 of the 14 available T5 positions are occupied by Zn in the room-temperature *I*4 structure. The distribution of Zn at room temperature in the *I*4 structure is separated into groups on the basis of Zn site occupancy. At the center of each group are four corner-connected tetrahedral sites with the highest Zn occupancy of 0.473(4) (the remainder is 0.51(5) Li) as shown in Figure 7a. Additional Zn occupied positions are located around these clusters, and Zn site occupancies decrease with an increase in distance from the group center (Figure 7a–c). These groups of Zn-rich sites are ordered to maximize the distance (15.78 Å) between neighboring groups (Figure 7a). This is distinct from the high-temperature *F*43*m* structure in which Zn is distributed throughout the structure on the disordered 48*h* T5 positions (Figure 7d). Though beyond the scope of the current study, this unique ordering of Zn positions in *I*4 Li_7_Zn_0.5_SiS_6_ could be further investigated through electron microscopy, pair distribution analysis, and large-box reverse Monte Carlo modeling.

### 3.4 Ionic Transport in Li_7_Zn_0.5_SiS_6_

**Figure 8:**
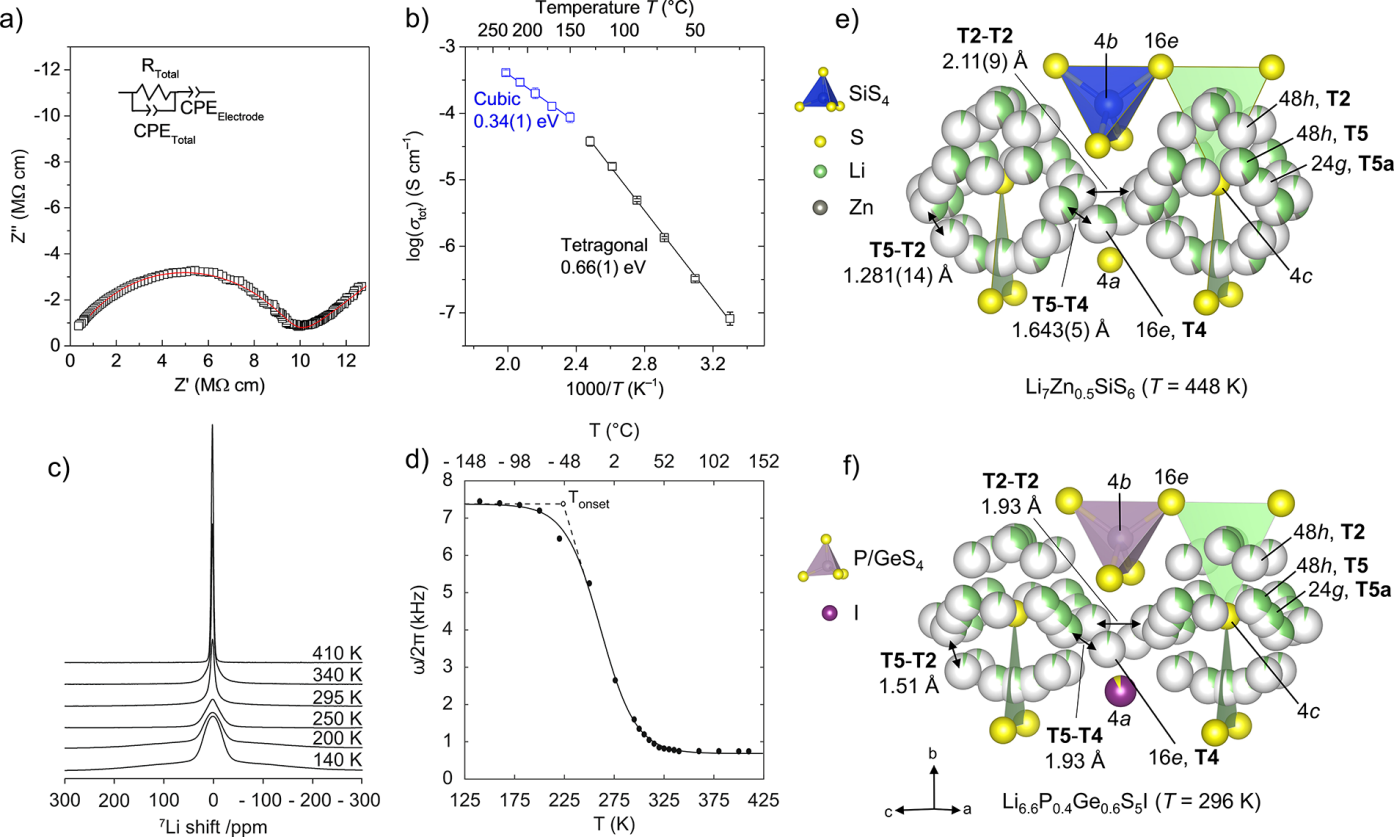
(a) Impedance complex plane plots Z* of Li_7_Zn_0.5_SiS_6_ measured at 303 K with the circuit used to model the data shown in the inset. (b) Arrhenius plot of the total conductivity of Li_7_Zn_0.5_SiS_6_ in the temperature range of 303–503 K with activation energies extracted from the plotted data. The change in activation energy coincides with the structural transition from tetragonal (black data points) to cubic (blue data points) symmetry observed in VT-XRD and DSC measurements. (c) ^7^Li NMR spectra of Li_7_Zn_0.5_SiS_6_ under static conditions plotted in the temperature range of 140–410 K. Data above the phase transition to cubic *F*43*m* symmetry have not been recorded. (d) Motional narrowing of the line width (full width at half-maximum) of the central ^7^Li NMR transition of tetragonal Li_7_Zn_0.5_SiS_6_. The solid line is a sigmoidal regression fit and is a guide to the eye. The onset temperature of motional narrowing (*T*_onset_) is indicated with a dashed line. Pseudo-octahedral Li^+^ cages centered around the 4*c* Wyckoff position in *F*43*m* (e) Li_7_Zn_0.5_SiS_6_ and (f) Li_6.6_P_0.4_Ge_0.6_S_5_I,^34^ highlighting the comparable site occupancies between both materials and the extensively delocalized distribution of Li in the high-temperature *F*43*m* Li_7_Zn_0.5_SiS_6_ structure. Double-headed arrows compare distances between sites likely involved in forming an extended conduction pathway.

The total ionic conductivity of Li_7_Zn_0.5_SiS_6_ was investigated via AC impedance spectroscopy, and local lithium ionic mobility was assessed through ^7^Li solid-state NMR. AC impedance measurements were carried out on a sintered pellet of Li_7_Zn_0.5_SiS_6_ of 81% theoretical density (≈30 mg of powder pressed into a 5 mm diameter pellet at a pressure of 125 MPa). A typical data set measured at 303 K under an inert Ar atmosphere is shown in Figure 8a. The impedance complex plane plots, Z*, consist of a higher-frequency arc and low frequency spike with the latter being associated with the capacitance of the sample–electrode interface that blocks the Li ions. The higher-frequency arc is attributed to the total conductivity (σ) of Li_7_Zn_0.5_SiS_6_ with an associated capacitance of 0.1 pF cm^–1^, corresponding to a permittivity of ≈1, consistent with the bulk response of the sample (Figure S13). To a first approximation, this arc can be modeled with an equivalent circuit consisting of a resistor in parallel with a constant phase element (CPE) (Figure 8a (inset)). Li_7_Zn_0.5_SiS_6_ presents a total conductivity of 1.0(2) × 10^–7^ S cm^–1^ at 303 K and 4.3(4) × 10^–4^ S cm^–1^ at 503 K. Values of total resistance were obtained in the temperature range from 303 to 503 K from the low-frequency intercept on the *Z*′ axis of the impedance arc and are shown in an Arrhenius plot in Figure 8b. Two separate regimes are observed, separated by a change in slope between 403 and 423 K, which coincides with the structural transition from tetragonal (*I*4) to cubic (*F*43*m*) symmetry observed via DSC and VT-PXRD measurements. Tetragonal Li_7_Zn_0.5_SiS_6_ has an activation energy of 0.66(1) eV, while cubic Li_7_Zn_0.5_SiS_6_ exhibits an activation energy of 0.34(1) eV above 423 K. Figure S14b–d shows the current–time curves of the Au|Li_7_Zn_0.5_SiS_6_|Au cell under DC polarization measured at −0.05, 0.1, and 0.7 V at which steady state current is achieved. The steady current is attributed to electronic leakage as two ion-blocking electrodes were used. Such a method provides an estimation of the upper limit of the electronic conductivity.^40^ The electronic conductivity (σ_e_) determined from the *I*–*E* curve is 5.1(4) × 10^–10^ S cm^–1^ at 303 K, which accounts for 0.51% of the overall conductivity, extracted through σ_e_ = *Id*/*EA* where *I* is the current, *d* is the pellet thickness, *E* is the polarization voltage, and *A* is the electrode area.

Insights into the local Li ion mobility in tetragonal Li_7_Zn_0.5_SiS_6_ were obtained through VT ^7^Li solid-state NMR. Static ^7^Li NMR spectra over the 140–410 K temperature range were recorded (Figure 8c) to observe the temperature dependence of the ^7^Li line width. At temperatures below ≈175 K, ^7^Li ion mobility is in the rigid lattice regime; hence, the ^7^Li central transition is broadened by the strong ^7^Li–^7^Li homonuclear dipolar coupling, and a line width of ≈7.5 kHz is observed. As the temperature is increased above 225 K, onset of motional narrowing (*T*_onset_) occurs and the increased motion of the ^7^Li spins continuously averages the dipolar interactions, causing the line width to decrease (Figure 8d). Using the temperature of this motional narrowing,^41^ an estimation of the activation energy for the local lithium ion diffusion process of 0.4 eV is obtained for tetragonal Li_7_Zn_0.5_SiS_6_. This value is lower than that obtained through AC impedance spectroscopy (0.66(1) eV) and captures the easier local hops between neighboring Li sites while the impedance data probe longer range translational lithium ion mobility. As the temperature is increased further, the ^7^Li NMR line widths continue to decrease up to >340 K where Li_7_Zn_0.5_SiS_6_ is in the fast-motional regime, resulting in an averaging of the dipolar interaction and giving rise to narrow spectra with line widths of ≈750 Hz. The Li ion jump rate, τ^–1^, at the temperature of the inflection point of the line narrowing curve is on the order of the central transition line width in the rigid lattice regime (≈7.5 kHz) and yields a value of 4.7 × 10^4^ s^–1^.

Argyrodite site hopping mechanisms that form conduction pathways for Li^+^ ions can be categorized into three types:^8^ “doublet” jumps that represent local motion between face-sharing 48*h* (T5) sites via the trigonal planar 24*g* (T5a) position, “intracage” jumps between neighboring 48*h* (T5) tetrahedra that share edges likely facilitated by 48*h* (T2) tetrahedra acting as intermediate sites, and “intercage” jumps where Li^+^ ions move between 48*h* (T5) sites in adjacent cages via either 48*h* (T2) or 16*e* (T4) positions, where the latter would produce a continuous face-sharing tetrahedral pathway for Li^+^ ions (Figure 8e). These T2 and T4 positions have partial occupancy in Li_7_Zn_0.5_SiS_6_ and likely form part of the extended conduction pathway. It is clear that the delocalization of lithium positions (Figure 8e) in the high-temperature cubic structure (>411 K) has a significant impact on Li ion mobility with an activation energy approximately half (0.34(1) eV) that of the room-temperature ordered tetragonal structure (0.66(1) eV). This significant decrease in activation energy between the *I*4 and *F*43*m* structures of Li_7_Zn_0.5_SiS_6_ can be understood by considering the Li–Li site distances that become available in the high-temperature structure. The intracage distances (T5–T5 and T5–T2) are very similar between the *I*4 and *F*43*m* structures, while the intercage distances (represented by T5–T4 and T2–T2) are much longer in the room-temperature *I*4 structure of Li_7_Zn_0.5_SiS_6_ (Table S7). In addition, the ordered Li sublattice in *I*4 Li_7_Zn_0.5_SiS_6_ reduces the number of these distances, severely limiting long-range Li ion diffusion.

The room-temperature conductivity of Li_7_Zn_0.5_SiS_6_ (σ_303 K_ = 1.0(2) × 10^–7^ S cm^–1^) is low and is comparable to other ordered argyrodite materials such as Li_7_PS_6_ and Li_6_PS_5_I, which have room-temperature conductivities reported in the range of 3.3 × 10^–8^ to 2 × 10^–6^ S cm^–1^.^11,12,42−45^ The conductivity in the high-temperature *F*43*m* regime (Li_7_Zn_0.5_SiS_6_, σ_503 K_ = 4.3(4) × 10^–4^ S cm^–1^) is an order of magnitude higher than that of *F*43*m* Li_7_PS_6_ (5.9 × 10^–5^ S cm^–1^) and Li_6_PS_5_I (1.3 × 10^–5^ S cm^–1^) at the same temperature.^43^ This results from the more delocalized distribution of Li in the high-temperature *F*43*m* structure (Figure 8e) compared against Li_6_PS_5_I and Li_7_PS_6_ in which only two sites (T5 and T5a) and one site (T5) are occupied, respectively.^7,9^ Interestingly, the low temperature ordered structures of argyrodites such as Li_7_PS_6_ (*Pna*2_1_) and Li_6_PS_5_I (*Cc*) exhibit lower activation energies than their higher temperature *F*43*m* polymorphs;^12,43^ this is not the case for Li_7_Zn_0.5_SiS_6_. The activation energy of *F*43*m* Li_7_Zn_0.5_SiS_6_ (0.34(1) eV) aligns well with the range reported (0.15–0.42 eV) for several superionic argyrodite conductors such as Li_6.35_P_0.65_Si_0.35_S_5_Br and Li_6.15_Al_0.15_Si_1.35_S_5.734_O_0.266_,^13,14,31,32,42,46^ indicating that, in the high-temperature *F*43*m* regime of Li_7_Zn_0.5_SiS_6_, comparable Li ion mobility is possible likely due to the availability of extensively delocalized Li sites that form part of the diffusion pathways. Though Li_7_Zn_0.5_SiS_6_ and Li_6.6_P_0.4_Ge_0.6_S_5_I both exhibit simultaneous occupation of the T5, T5a, T2, and T4 sites, specific site occupancies and distances between these sites that form part of the extended conduction pathway are considerably different.^34^ In *F*43*m* Li_7_Zn_0.5_SiS_6_, the T5 sites are occupied by 0.405(8) Li and 0.0414(5) Zn with small Li occupancy of 0.099(7) on the T5a site, whereas the local Li distribution in Li_6.6_P_0.4_Ge_0.6_S_5_I is more homogeneous with the T5 site occupied by 0.318 Li and T5a site, by 0.358 Li. The additional T2 and T4 sites are occupied with 0.037 and 0.042 Li in Li_6.6_P_0.4_Ge_0.6_S_5_I and 0.048(5) and 0.243(6) Li in Li_7_Zn_0.5_SiS_6_. The T5–T5 distance of 1.26 Å in Li_6.6_P_0.4_Ge_0.6_S_5_I is much shorter than that of 1.896(7) Å for Li_7_Zn_0.5_SiS_6_, and this consequently means that the T5–T2 intracage distances are much shorter (1.281(14) Å) in Li_7_Zn_0.5_SiS_6_ compared to that (1.51 Å) in Li_6.6_P_0.4_Ge_0.6_S_5_I (see Table S7). Related to this, the intercage T5–T4 distances are much shorter (1.643(5) Å) for Li_7_Zn_0.5_SiS_6_ compared to that (1.93 Å) for Li_6.6_P_0.4_Ge_0.6_S_5_I, while the T2–T2 intercage distances are 2.11(9) Å for Li_7_Zn_0.5_SiS_6_ compared to 1.93 Å for Li_6.6_P_0.4_Ge_0.6_S_5_I (Figure 8e,f).

The conductivity of *F*43*m* Li_7_Zn_0.5_SiS_6_ is higher than that of other argyrodites, which show order–disorder behavior in the same regime (e.g., Li_6_PS_5_I and Li_7_PS_6_), and although the conductivity is lower than those reported for highly disordered *F*43*m* argyrodites, Li_7_Zn_0.5_SiS_6_ exhibits a comparable activation energy, indicating that Li ion mobility is similar. This can be attributed to the presence of Zn^2+^ on the Li sublattice in Li_7_Zn_0.5_SiS_6_. The small subset of T5 sites occupied by Zn in *I*4 Li_7_Zn_0.5_SiS_6_ compared to the disordered distribution across all T5 sites at higher temperatures in *F*43*m* Li_7_Zn_0.5_SiS_6_ indicates that a low level of Zn^2+^ mobility exists to stabilize the RT ordering. It is unlikely that Zn^2+^ contributes significantly to the measured ionic conductivity of Li_7_Zn_0.5_SiS_6_ as the Zn distribution is limited to T5 sites only, which implies that local mobility does not extend beyond intracage T5–T5 hops. The incorporation of Zn onto the Li sites may lead to blocking of Li ions as postulated for Li_3.5_Ge_1.5_P_0.5_S_6_ and Li_6.15_Al_0.15_Si_1.35_S_5.734_O_0.266_;^32,39,47^ however, it is the presence of Zn in this concentration that leads to the occupation of the additional T2 and T4 sites that provide a large number of possible routes for Li ion mobility such that possible blocking effects become negligible. The overload of the mobile cation sites with a total cation (Li and Zn) content of 7.5 further enables the delocalization of Li sites by ensuring sufficient occupancy of the additional sites. Anion disorder in argyrodites is known to impact significantly on Li ion conductivity by influencing the energy landscape for Li^+^ ion mobility.^5^ Disorder on the anion sublattice, which can be controlled through anionic or cationic substitutions or the synthesis method,^14,48−51^ yields an inhomogeneous distribution of charge density generating spatially diffuse and delocalized distributions of Li^+^ ions, resulting in higher ionic mobility. Li_7_Zn_0.5_SiS_6_ has a single cation (Si^4+^) on the 4*b* position and is a single anion (S^2–^ only) system; however, it achieves a highly delocalized Li distribution in the *F*43*m* regime comparable to that of the highest conducting argyrodites. It is possible through additional substitution chemistry that the disordered *F*43*m* structure of Li_7_Zn_0.5_SiS_6_ could be stabilized to lower temperatures or a more inhomogeneous charge density distribution could be achieved to further improve the ionic conductivity.

## 4 Conclusions

Li_7_Zn_0.5_SiS_6_, the first argyrodite with a tetragonal (*I*4) crystal structure has been synthesized and characterized through a combination of X-ray, neutron powder diffraction, NMR, and impedance spectroscopy. Li_7_Zn_0.5_SiS_6_ is a rare example of an argyrodite with >7 mobile cations, and the incorporation of a small amount (6.7%) of Zn into the Li sublattice stabilizes a complex tetragonal superstructure of *F*43*m* argyrodites with a unique Li distribution previously unseen in these materials. At high temperatures, Li_7_Zn_0.5_SiS_6_ adopts a *F*43*m* cubic argyrodite structure with simultaneous occupation of the 48*h* (T5), 24*g* (T5a), 48*h* (T2), and 16*e* (T4) sites, a unique site distribution that provides a range of possible pathways for ion hopping, yielding a higher conductivity than other Li-only materials such as Li_7_PS_6_ that show order–disorder behavior. This combination of sites leads to an extensively delocalized Li distribution forming a continuous face-sharing tetrahedral pathway for Li ions that is accessible in *F*43*m* Li_7_Zn_0.5_SiS_6_. This is achieved via incorporation of Zn and overloading of the mobile cation content, which has a profound effect on the structure of Li_7_Zn_0.5_SiS_6_. This significantly impacts the Li ion conductivity and reveals how property-controlling cation ordering in argyrodites arises from compositional control. The realization of this unique ordering pattern offers new routes to tuning and further understanding the unexplored chemistries of argyrodite materials, in particular the unexpected increase in conductivity in the high-temperature disordered form compared to some lithium-only argyrodites.

## Supporting Information Available

Further details on the synthetic isolation and structural characterization (diffraction, NMR, Raman) of Li_7_Zn_0.5_SiS_6_, impedance results, elemental analysis (ICP), and tables of bond distances and angles in high-temperature *F*43*m* and room-temperature *I*4 structures (PDF)
Li_7_Zn_0.5_SiS_6_ powder CIF for HT structure (CIF)
Li_7_Zn_0.5_SiS_6_ powder CIF for RT structure (CIF)
Li_7_Zn_0.5_SiS_6_ RT structure only, with dummy atoms used for bond distance restraints during refinement (CIF)

## Supplementary Material

cm2c00320_si_001.pdf

cm2c00320_si_002.cif

cm2c00320_si_003.cif

cm2c00320_si_004.cif

## Notes

Room-temperature and high-temperature crystal structures of Li_7_Zn_0.5_SiS_6_ are deposited with ICSD numbers 2144376-2144384 and 2144370-2144373, respectively. Underlying data collected as part of this work is available via the University of Liverpool data repository at https://datacat.liverpool.ac.uk/id/eprint/1603.

## Dedication

This paper is dedicated to Professor John Goodenough.
