# Supplementary material for: Cation Disorder and Large Tetragonal Supercell Ordering in the Li-Rich Argyrodite Li7Zn0.5SiS6
Source: Chem Mater. 2022 Apr 18;34(9):4073–87. doi: 10.1021/acs.chemmater.2c00320 (PMC9097155; doi:10.1021/acs.chemmater.2c00320)
Supplement: Supplementary file 1 — cm2c00320_si_001.pdf [file cm2c00320_si_001.pdf]

Supplementary Information

for

Cation Disorder and Large Tetragonal Supercell Ordering in the Li-rich Argyrodite $Li_7Zn_{0.5}SiS_6$

Bernhard T. Leube,[1] Christopher M. Collins,[1] Luke M. Daniels,[1] Benjamin B. Duff,[1,2] Yun Dang,[1] Ruiyong Chen,[1] Michael W. Gaultois,[1,3] Troy D. Manning,[1] Frédéric Blanc,[1,2,3] Matthew S. Dyer,[1,3] John B. Claridge,[1,3] Matthew J. Rosseinsky[1,3]*

[1] Department of Chemistry, University of Liverpool, Crown Street, L69 7ZD, Liverpool, UK

[2] Stephenson Institute for Renewable Energy, University of Liverpool, Peach Street, L69 7ZF, Liverpool, UK

[3] Leverhulme Research Centre for Functional Materials Design, Materials Innovation Factory, Oxford Street, L7 3NY, UK

*Corresponding author: rossein@liverpool.ac.uk

Contents

Synthetic isolation of $Li_7Zn_{0.5}SiS_6$	Figures S1-S4	Pages 2-3
Further structural characterisation	Figures S5-S12	Pages 4-7
Further property characterisation	Figures S13-S14	Page 8
Elemental analysis	Table S1	Page 9
Structural tables	Tables S2-S7	Pages 9-33
References		Page 33

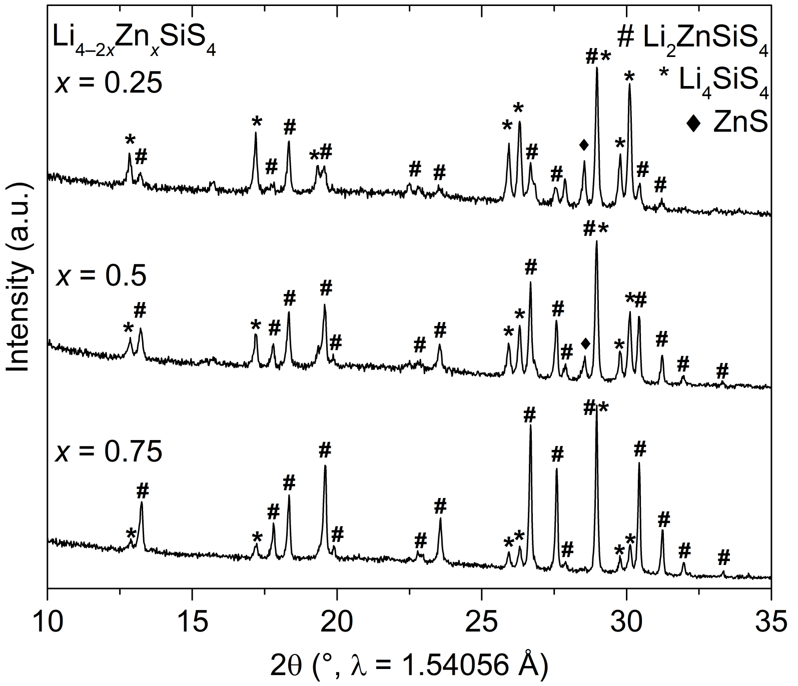

Figure S1. PXRD patterns measured on powder samples synthesised along the $Li_{4-2x}Zn_xSiS_4$ tie line in the $LiS_{0.5}$-ZnS-SiS_2 phase field (Figure 1a and b, purple line). There is no evidence for a solid solution between Li_4SiS_4 and Li_2ZnSiS_4 as only known phases are observed (Li_4SiS_4 (*), Li_2ZnSiS_4 (#), ZnS (◆)).

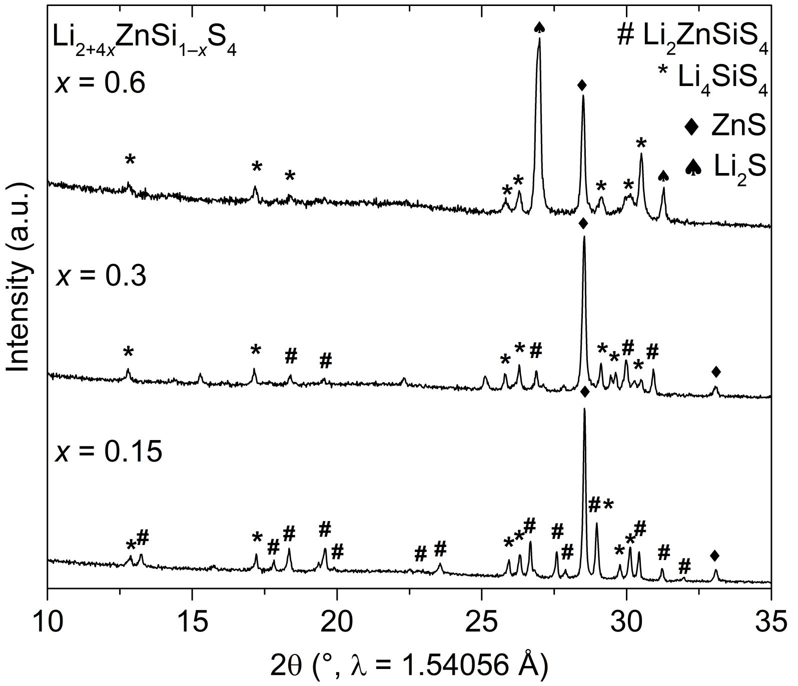

Figure S2. PXRD patterns measured on powder samples synthesised along the $Li_{2+4x}ZnSi_{1-x}S_4$ tie line in the $LiS_{0.5}$-ZnS-SiS_2 phase field (Figure 1a and b, green line). All observed peaks can be assigned to known materials (Li_4SiS_4 (*), Li_2ZnSiS_4 (#), ZnS (◆), Li_2S (♠)).

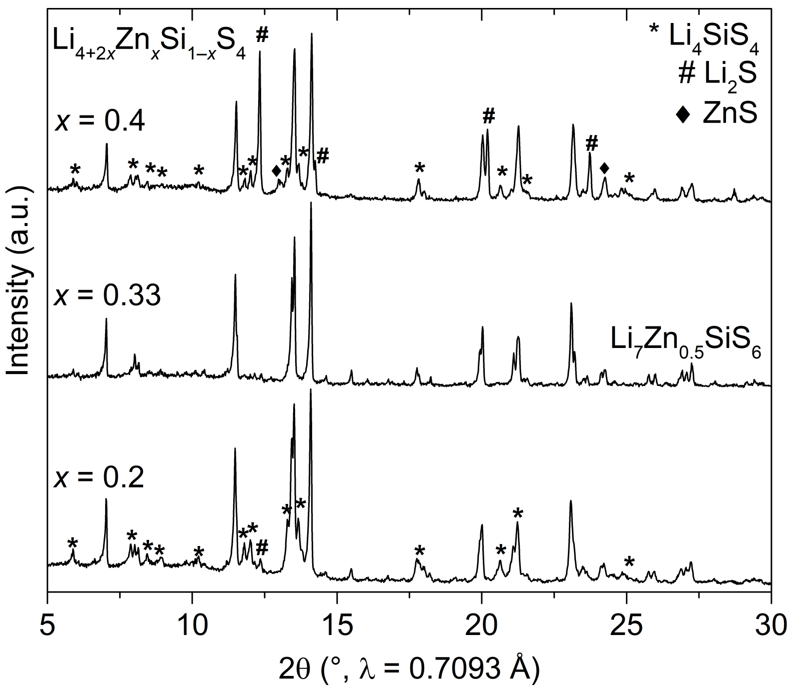

Figure S3. PXRD patterns measured on powder samples synthesised along the $Li_{4+2x}Zn_xSi_{1-x}S_4$ tie line in the $LiS_{0.5}$-ZnS-SiS_2 phase field (Figure 1a and b, blue line). This includes the new $Li_7Zn_{0.5}SiS_6$ argyrodite material (x = 0.33 in $Li_{4+2x}Zn_xSi_{1-x}S_4$) which gives a phase pure powder (Figure 1a and b, orange star), while powders from compositions on either side of x = 0.33 contain known phases as impurities (Li_4SiS_4 (*), Li_2S (#), ZnS (◆)).

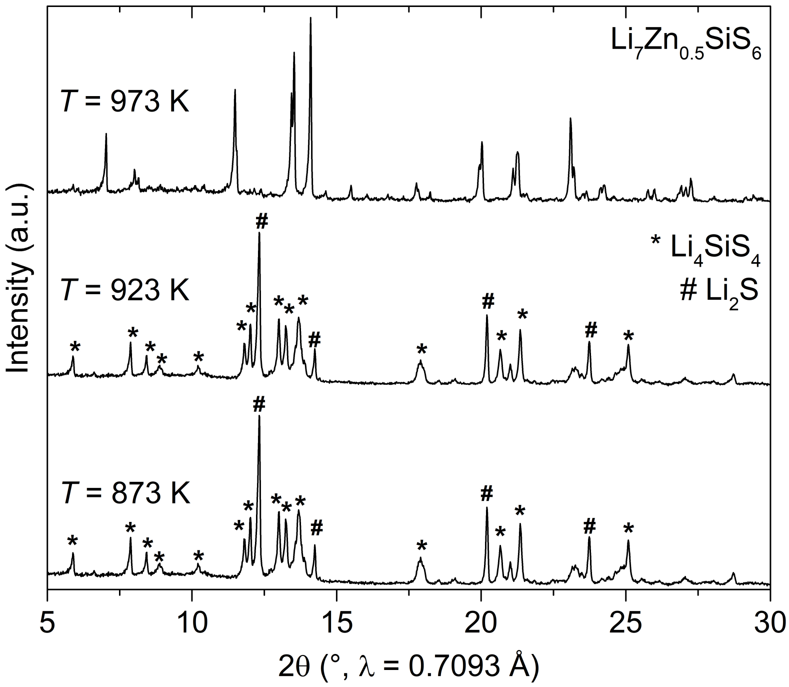

Figure S4. PXRD patterns measured from $Li_7Zn_{0.5}SiS_6$ powder samples synthesised in the temperature range 873-973 K for 24 h. Samples synthesised at temperatures < 973 K yield mixtures of Li_4SiS_4 (*) and Li_2S (#), and the new $Li_7Zn_{0.5}SiS_6$ phase is formed only at 973 K (top pattern).

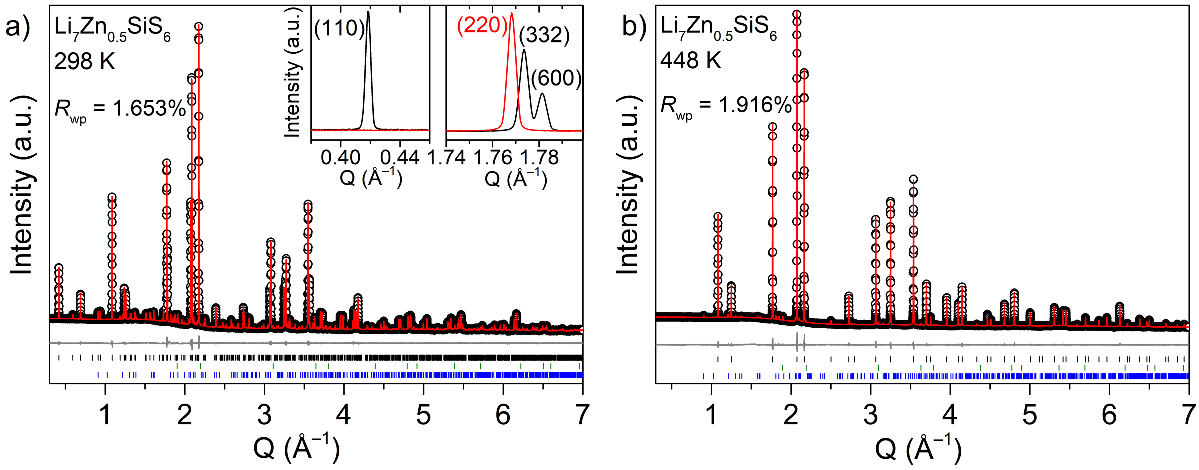

Figure S5. a) Le Bail fit against SXRD data of $Li_7Zn_{0.5}SiS_6$ measured at room temperature (298 K) using $I\bar{4}$ symmetry (the lowest symmetry tetragonal space group consistent with the I – – – diffraction symbol). The inset compares reflections from $I\bar{4}$ (T = 298 K, black line) and $F\bar{4}3m$ (T = 448 K, red line) structures in $Li_7Zn_{0.5}SiS_6$. b) Le Bail fit against SXRD data of $Li_7Zn_{0.5}SiS_6$ measured at 448 K using $F\bar{4}3m$ symmetry. I_{obs} (black circles), I_{calc} (red line), I_{obs}-I_{calc} (grey line), and Bragg reflections (black tick marks for $Li_7Zn_{0.5}SiS_6$, green for Li_2S, blue for Li_4SiS_4) are shown.

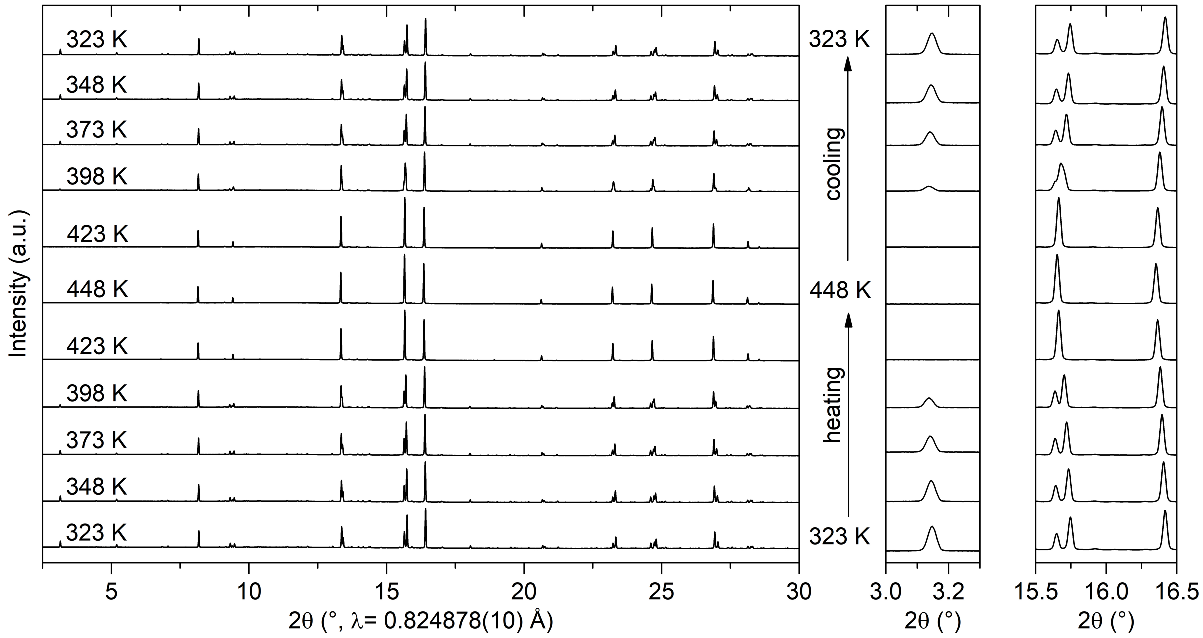

Figure S6. Synchrotron X-ray powder diffraction data (SXRD) measured in the temperature range 323-448 K highlighting the reversible phase transition from $I\bar{4}$ to $F\bar{4}3m$ symmetry in $Li_7Zn_{0.5}SiS_6$ above 411 K. Enlargements of the (110) reflection, and the (303), (631), and (602) reflections are shown on the right.

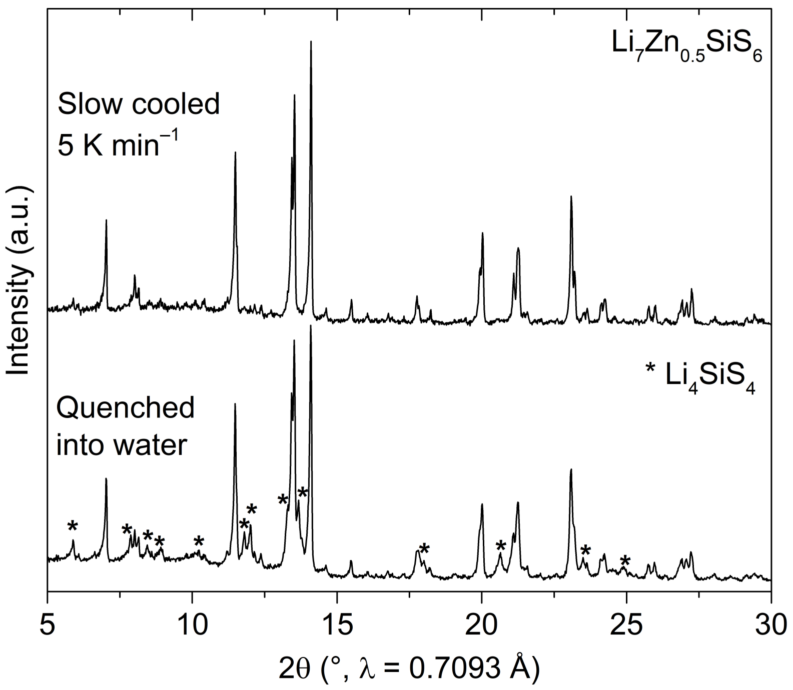

Figure S7. PXRD patterns measured from $Li_7Zn_{0.5}SiS_6$ powder samples synthesised under the optimal reaction conditions (two firings at 973 K for 24 hours each using heating and cooling rates of 5 K min^{-1}) and a sample quenched into a water bath from 973 K after the same reaction duration. Tetragonal $I\bar{4}$ $Li_7Zn_{0.5}SiS_6$ is the majority phase following quenching, with Li_4SiS_4 (*) as a secondary phase.

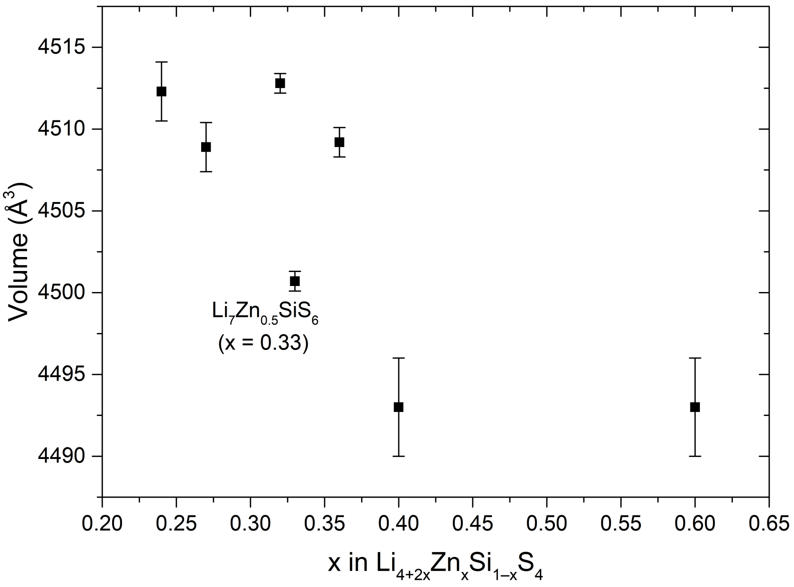

Figure S8. Variation in the unit cell volume of tetragonal $I\bar{4}$ $Li_7Zn_{0.5}SiS_6$ as a function of composition (x in $Li_{4+2x}Zn_xSi_{1-x}S_4$) extracted from samples which contain the new phase (Figure 2a and b). $Li_7Zn_{0.5}SiS_6$ corresponds to x = 0.33.

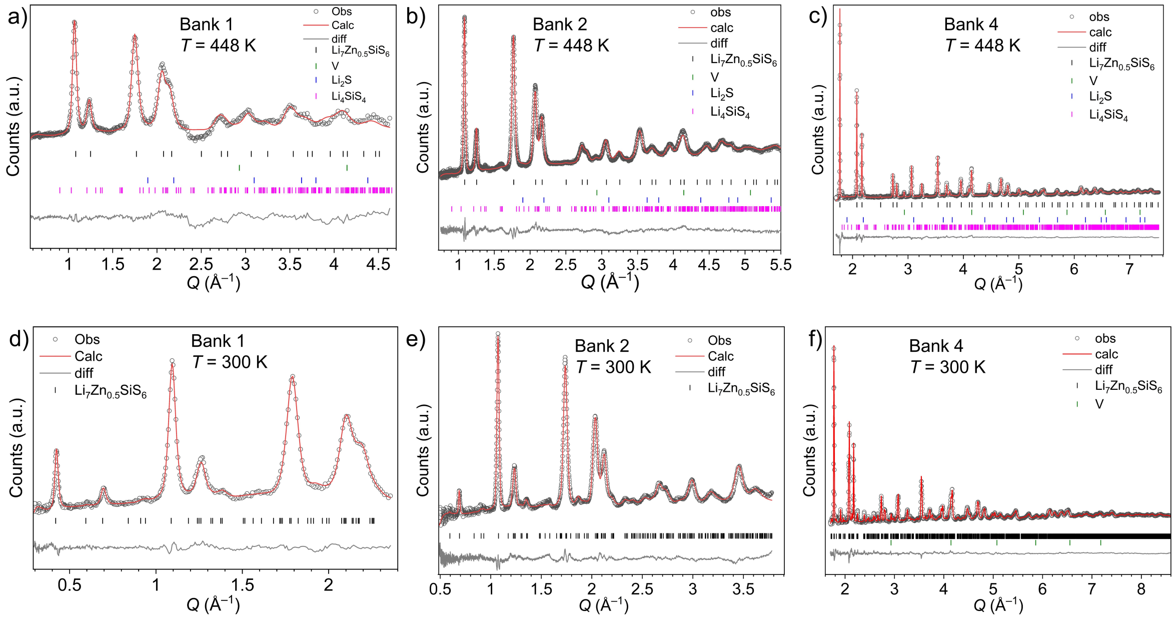

Figure S9. a-c) Rietveld refinement against NPD data from Bank 1, Bank 2, and Bank 4 (Polaris, ISIS) at 448 K of the high-temperature structure of $Li_7Zn_{0.5}SiS_6$ with $F\bar{4}3m$ symmetry. d-f) Rietveld refinement against NPD data from Bank 1, Bank 2, and Bank 4 (Polaris, ISIS) at 300 K of the room-temperature structure of $Li_7Zn_{0.5}SiS_6$ with $I\bar{4}$ symmetry. Traces I_{obs} (black circles), I_{calc} (red line), I_{obs}-I_{calc} (grey line), and Bragg reflections are shown (black tick marks for $Li_7Zn_{0.5}SiS_6$, green for V metal, blue for Li_2S, pink for Li_4SiS_4). The high-temperature refinement has R_{wp} = 3.58 % and χ^2 = 38.2 for 109 refined parameters, and the room-temperature refinement has R_{wp} = 5.60% and χ^2 = 14.8 for 245 refined parameters.

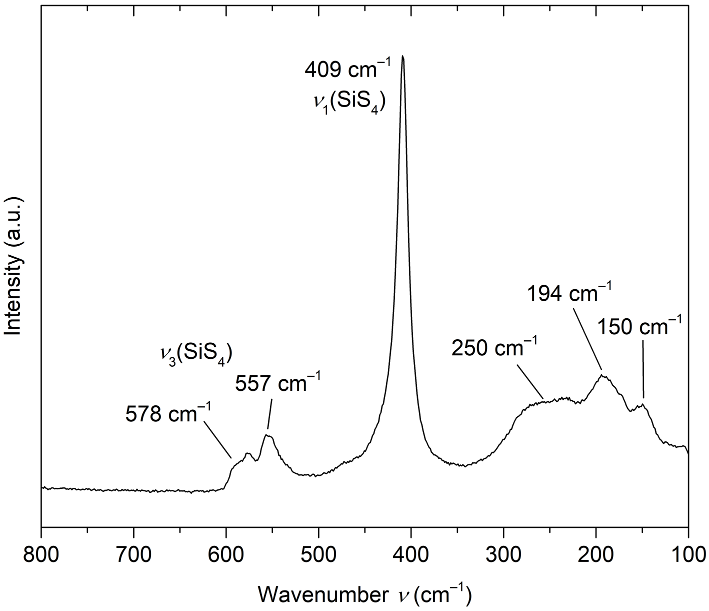

Figure S10. Raman spectrum of $Li_7Zn_{0.5}SiS_6$ measured at 298 K using a 523 nm incident wavelength laser. The peaks observed around 560 cm^{-1} and 410 cm^{-1} are assigned to $\nu_3(SiS_4)$ and $\nu_1(SiS_4)$ modes respectively, based on mode assignment reported for Cu_8MX_6 (M = Si, Ge and X = S, Se).[1]

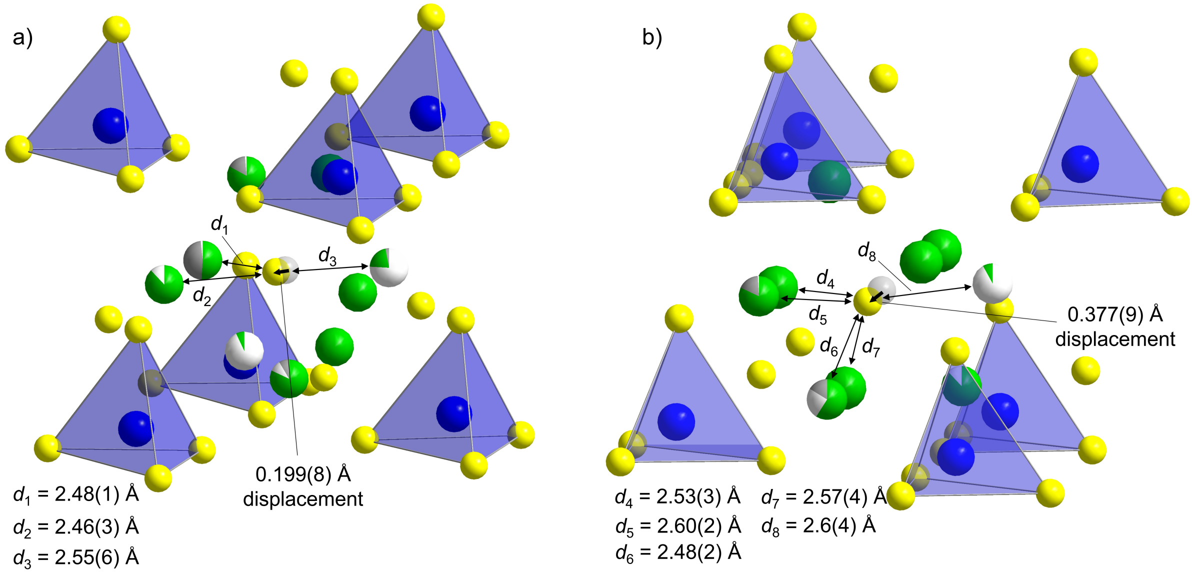

Figure S11. Two Friauf icosahedra (SS_{12} polyhedra) formed from six SiS_4^{4-} polyanions surrounding S^{2-} anions (8*g* positions) that occupy the octahedral voids generated from the cubic close-packing of SiS_4^{4-} polyanions in room-temperature $I\bar{4}$ $Li_7Zn_{0.5}SiS_6$. Translucent grey atoms show the central S^{2-} anions in the perfect octahedron centre as is the case in high-temperature $F\bar{4}3m$ $Li_7Zn_{0.5}SiS_6$. These two S^{2-} anions are displaced from the octahedral centre in room-temperature $I\bar{4}$ $Li_7Zn_{0.5}SiS_6$ by a) 0.199(8) Å and b) 0.377(9) Å. The S^{2-} anions displace away from partially occupied Li sites towards sites occupied by both Li and Zn increasing the valence of these positions. The ordering of Zn and Li sites in $I\bar{4}$ $Li_7Zn_{0.5}SiS_6$ results in these displacements of S^{2-} anions.

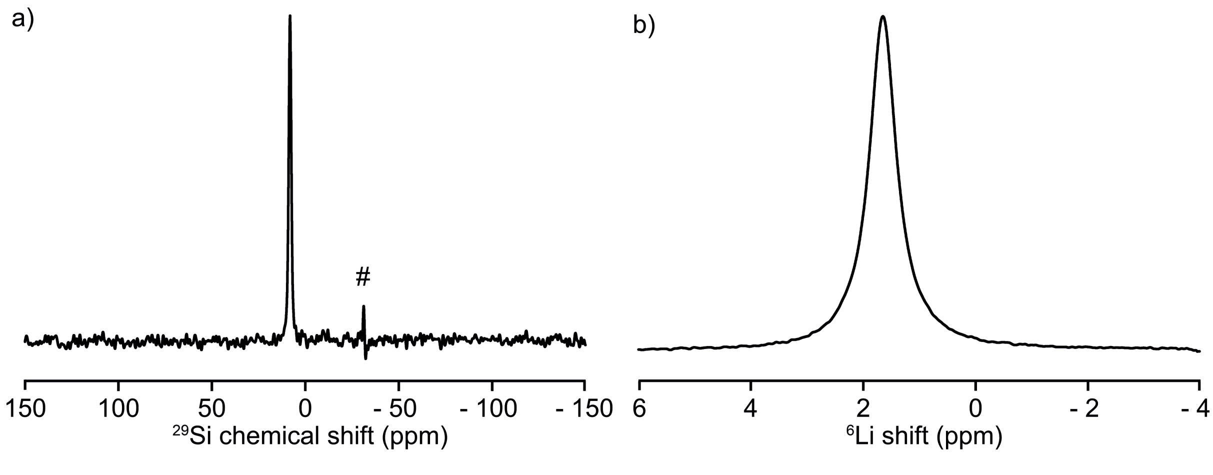

Figure S12. a) ^{29}Si and b) ^{6}Li MAS NMR spectra of $Li_7Zn_{0.5}SiS_6$ at room temperature. The hash sign (#) denotes the centre spike from the carrier frequency. Both spectra were collected at 9.4 T and with a MAS rate of 10 kHz.

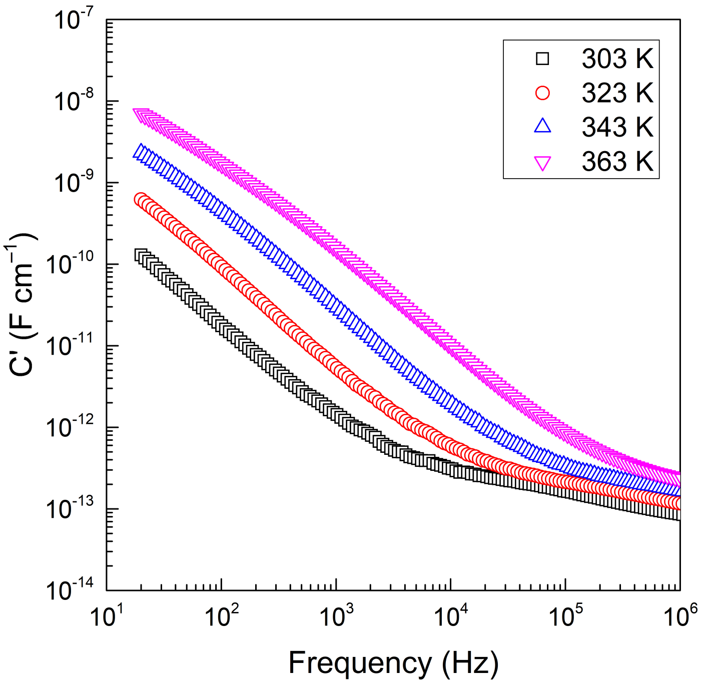

Figure S13. Capacitance vs frequency in the 303-363 K temperature range measured from $Li_7Zn_{0.5}SiS_6$. The limiting high-frequency plateau has a value of 0.1 pF cm^{-1}, corresponding to a permittivity of ≈1, and thus represents the bulk response of the sample.

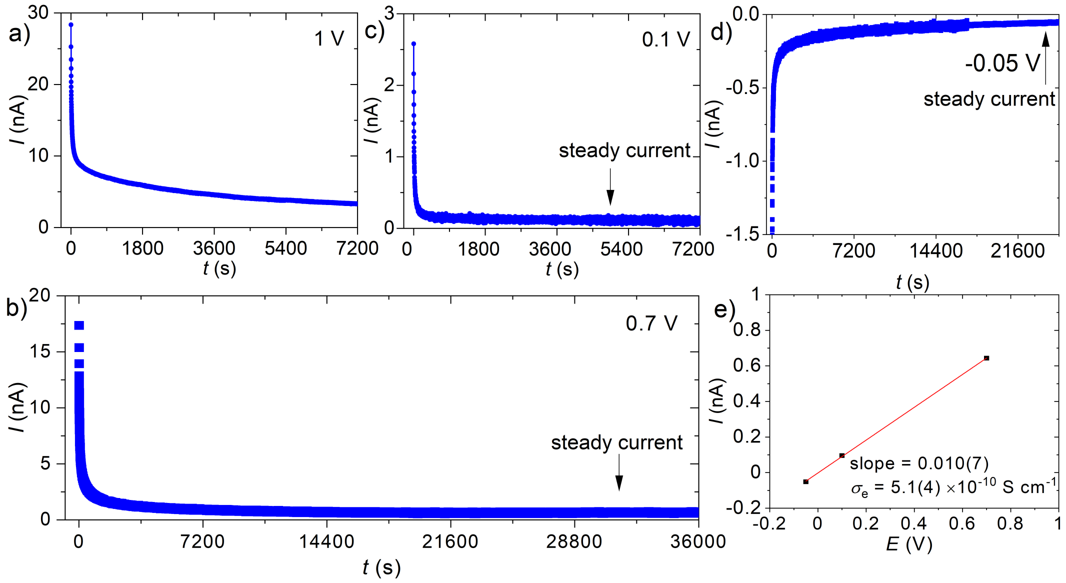

Figure S14. a-d) *I-t* curves measured between 1 and –0.05 V at 303 K for the Au|$Li_7Zn_{0.5}SiS_6$|Au cell under DC polarization. Steady current was obtained at 0.7, 0.1 and –0.05 V and are plotted in e) *I-E* curve. Electronic conductivity was calculated to be 5.1(4) × 10^{-10} S cm^{-1} at 303 K from the DC polarization data. This value is lower than values reported for Li_6PS_5X (X = Cl, Br) type materials.[2-5]

Table S1. Measured composition of $Li_7Zn_{0.5}SiS_6$. Elemental analysis is performed via inductively coupled plasma atomic emission spectroscopy (ICP-AES). The measured values are normalised to give six sulfur allowing for easy comparison with the expected composition.

Element	Measured wt%	Normalised	Expected composition
Li	15.90(5)	7.07(2)	7.00
Zn	10.00(5)	0.472(2)	0.50
Si	9.725(5)	1.068(6)	1.00
S	62.35(65)	6.00(6)	6.00

Table S2. Room-temperature structure of $Li_7Zn_{0.5}SiS_6$ at 300 K in space group $I\bar{4}$ with refined unit cell parameters a = b = 21.15065(2) Å, c = 10.05640(15) Å, and total refined composition of $Li_{7.00(8)}Zn_{0.480(5)}SiS_6$.

Site	Wyckoff position	*x*	*y*	*Z*	U_{iso} (Å^2)	Occ.
S01	8*g*	0.3209(3)	0.6541(3)	−0.0051(7)	0.01614(14)	1
S02	2*d*	0	0.5	0.75	0.01614(14)	1
S03	8*g*	0.2566(3)	0.6749(3)	0.6236(6)	0.01614(14)	1
S04	8*g*	−0.0755(3)	0.6648(3)	0.6209(6)	0.01614(14)	1
S05	8*g*	0.0056(3)	0.5853(3)	0.3752(5)	0.01614(14)	1
S06	8*g*	0.0884(3)	0.6657(3)	0.6147(6)	0.01614(14)	1
S07	8*g*	0.3395(3)	0.5911(3)	0.3754(6)	0.01614(14)	1
S08	8*g*	0.1663(3)	0.9994(3)	0.2376(5)	0.01614(14)	1
S09	8*g*	0.1675(3)	0.6578(2)	0.2414(6)	0.01614(14)	1
S10	8*g*	0.0063(3)	0.7494(3)	0.3769(7)	0.01614(14)	1
S11	8*g*	0.4204(3)	0.6700(3)	0.6212(7)	0.01614(14)	1
S12	8*g*	0.3372(3)	0.7521(3)	0.3777(6)	0.01614(14)	1
S13	8*g*	0.4163(3)	0.5042(3)	0.1190(6)	0.01614(14)	1
S14	8*g*	0.1677(3)	0.5092(2)	0.4963(7)	0.01614(14)	1
S15	2*a*	0	1	0	0.01614(14)	1
Si1	8*g*	0.0058(3)	0.6661(3)	0.4981(7)	0.0108(4)	1
Si2	8*g*	0.3399(3)	0.6721(3)	0.4943(7)	0.0108(4)	1
Si3	2*b*	0.5	0.5	0	0.0108(4)	1
Zn1	8*g*	0.1156(3)	0.5609(3)	0.3044(6)	0.0239(6)	0.473(4)
Li1	8*g*	0.1156(3)	0.5609(3)	0.3044(6)	0.0239(6)	0.51(5)
Zn2	8*g*	0.1004(6)	0.5533(6)	0.6886(13)	0.0239(6)	0.158(4)
Li2	8*g*	0.1004(6)	0.5533(6)	0.6886(13)	0.0239(6)	0.83(4)
Zn3	8*g*	0.2646(6)	0.6013(6)	0.1956(13)	0.0239(6)	0.171(6)
Li3	8*g*	0.2646(6)	0.6013(6)	0.1956(13)	0.0239(6)	0.82(7)
Zn4	8*g*	0.2041(6)	0.6609(6)	0.0148(15)	0.0239(6)	0.162(5)
Li4	8*g*	0.2041(6)	0.6609(6)	0.0148(15)	0.0239(6)	0.59(6)
Li5	8*g*	0.1683(16)	0.9648(14)	0.474(3)	0.0239(6)	1
Li6	8*g*	0.0510(17)	0.7286(15)	0.818(4)	0.0239(6)	1
Li7	8*g*	0.0654(18)	0.7150(17)	0.181(4)	0.0239(6)	1
Li8	8*g*	0.2733(15)	0.5947(15)	0.796(4)	0.0239(6)	1
Li9	8*g*	0.4044(14)	0.6187(16)	0.170(4)	0.0239(6)	1
Li10	8*g*	0.161(3)	0.389(2)	0.482(5)	0.0239(6)	0.21(6)
Zn5	8*g*	0.161(3)	0.389(2)	0.482(5)	0.0239(6)	0.031(5)
Li11	8*g*	0.0714(14)	0.4495(14)	0.557(3)	0.0239(6)	1

Li12	8*g*	0.4525(15)	0.5998(15)	0.441(3)	0.0239(6)	1
Li13	8*g*	0.0833(15)	0.8343(13)	0.367(3)	0.0239(6)	1.00(3)
LI14	4*e*	0.0619(19)	0.9441(18)	0.187(4)	0.0239(6)	0.86(4)
Li15	8*g*	0	1	0.23(2)	0.0239(6)	0.14(5)
Li16	8*g*	0.2887(9)	0.5089(9)	0.510(2)	0.0239(6)	0.82(8)
Zn6	8*g*	0.2887(9)	0.5089(9)	0.510(2)	0.0239(6)	0.085(6)
Li17	8*g*	0.27(2)	0.56(2)	0.57(4)	0.0239(6)	0.07(5)
Li18	8*g*	0.1640(15)	0.7528(15)	0.631(3)	0.0239(6)	1
Li19	8*g*	0.2812(18)	0.7378(19)	0.824(4)	0.0239(6)	0.88(4)
Li20	8*g*	0.258(9)	0.671(13)	0.38(2)	0.0239(6)	0.12(3)
Li21	8*g*	0.1771(16)	0.6249(16)	0.477(4)	0.0239(6)	0.88(4)
Li22	8*g*	0.127(17)	0.739(18)	0.42(4)	0.0239(6)	0.08(4)

Table S3. Bond distances obtained from Rietveld refinement against SXRD and NPD data of the high temperature $F\bar{4}3m$ structure (T = 448 K) of $Li_7Zn_{0.5}SiS_6$.

Site 1	Site 2	Distance (Å)
Li1	Li2	0.953(10)
Li2	Zn1	0.953(10)
Li1	Li4	1.281(14)
Li4	Zn1	1.281(14)
Li1	Li3	1.643(5)
Li3	Zn1	1.643(5)
Li1	Li1	1.896(7)
Zn1	Zn1	1.896(7)
Li2	Li4	1.93(3)
Li4	Li4	1.99(8)
S2	Si1	2.1147(11)
Li2	Li3	2.20(4)
Li3	S3	2.23(2)
Li2	S2	2.23(4)
Li3	Li4	2.27(3)
Li4	S3	2.40(5)
Li2	S1	2.45(6)
Li1	S2	2.475(2)
S2	Zn1	2.475(2)
Li3	S2	2.518(12)
Li4	S1	2.52(5)
Li1	S1	2.537(4)
S1	Zn1	2.537(4)
Li1	S3	2.608(4)
S3	Zn1	2.608(4)
Li4	S2	2.61(4)
Li4	Si1	3.06(5)

Table S4. Angles obtained from Rietveld refinement against SXRD and NPD data of the high temperature $F\bar{4}3m$ structure (T = 448 K) of $Li_7Zn_{0.5}SiS_6$.

Site 1	Site 2	Site 3	Angle at site 2 (°)	Site 1	Site 2	Site 3	Angle at site 2 (°)
Li1	Li1	Li1	120	Li4	Zn1	S2	132.43(9)
Li1	Li1	Li2	6(4)	Li4	Zn1	S3	156.3(9)
Li1	Li1	Li3	118.6(8)	Li4	Zn1	Zn1	30.10(7)
Li1	Li1	Li4	116(2)	S1	Li1	Li1	62.02(7)
Li1	Li1	S1	147.43(3)	S1	Li1	Li2	74(4)
Li1	Li1	S2	99.57(8)	S1	Li1	Li3	173.4(8)
Li1	Li1	S3	57.20(6)	S1	Li1	Li4	59.7(11)
Li1	Li2	Li1	169(7)	S1	Li1	S2	111.92(10)
Li1	Li2	Li3	156.76(7)	S1	Li1	Zn1	62.02(7)
Li1	Li2	Li4	56.2(17)	S1	Li2	Li1	84(4)
Li1	Li2	S1	54.0(10)	S1	Li2	Li3	127.9(15)
Li1	Li2	S2	90.9(3)	S1	Li2	Li4	69(2)
Li1	Li2	Zn1	169(7)	S1	Li2	S2	125.1(14)
Li1	Li3	Li1	118.7(4)	S1	Li2	Zn1	84(4)
Li1	Li3	Li2	107.4(9)	S1	Li4	Li1	76(2)
Li1	Li3	Li4	59.1(9)	S1	Li4	Li2	65(2)
Li1	Li3	S2	120.4(8)	S1	Li4	Li3	120.9(11)
Li1	Li3	S3	116.5(4)	S1	Li4	Li4	171.8(11)
Li1	Li4	Li1	137(4)	S1	Li4	S3	124.3(19)
Li1	Li4	Li2	84.5(16)	S1	Li4	Zn1	76(2)
Li1	Li4	Li3	89.0(14)	S1	Zn1	Li1	62.02(7)
Li1	Li4	Li4	127.8(11)	S1	Zn1	Li2	74(4)
Li1	Li4	S1	60.4(11)	S1	Zn1	Li3	173.4(8)
Li1	Li4	S2	118.5(17)	S1	Zn1	Li4	59.7(11)
Li1	Li4	S3	63.9(12)	S1	Zn1	S2	111.92(10)
Li1	S1	Li1	114.85(7)	S1	Zn1	Zn1	62.02(7)
Li1	S1	Li2	158.06(8)	S2	Li1	Li1	102.51(7)
Li1	S1	Li4	64.7(4)	S2	Li1	Li2	64(2)
Li1	S2	Li1	69.61(17)	S2	Li1	Li3	72.2(5)
Li1	S2	Li2	129.3(9)	S2	Li1	Li4	81(2)
Li1	S2	Si1	106.28(7)	S2	Li1	S2	94.97(13)
Li1	S3	Li1	65.61(13)	S2	Li1	Zn1	102.51(7)
Li1	S3	Li3	88.00(5)	S2	Li2	Li1	93.3(19)
Li1	S3	Li4	66.7(9)	S2	Li2	Li3	69.3(14)
Li1	Zn1	Li2	6(4)	S2	Li2	Li4	77.4(14)
Li1	Zn1	Li3	118.6(8)	S2	Li2	S2	110(3)
Li1	Zn1	Li4	116(2)	S2	Li2	Zn1	93.3(19)
Li1	Zn1	S1	147.43(3)	S2	Li3	Li1	69.4(3)
Li1	Zn1	S2	99.57(8)	S2	Li3	Li2	56.0(7)
Li1	Zn1	S3	57.20(6)	S2	Li3	Li4	102.9(9)
Li2	Li1	Li1	97.7(9)	S2	Li3	S2	92.9(6)
Li2	Li1	Li2	110(4)	S2	Li3	S3	123.2(4)

Li2	Li1	Li3	124.0(11)	S2	Li3	Zn1	69.4(3)
Li2	Li1	Li4	71.3(10)	S2	Li4	Li1	138.3(6)
Li2	Li1	S1	51.3(10)	S2	Li4	Li2	130(2)
Li2	Li1	S2	96.3(4)	S2	Li4	Li3	61.6(8)
Li2	Li1	S3	77.8(7)	S2	Li4	Li4	66.1(9)
Li2	Li3	Li1	120.60(14)	S2	Li4	S1	108.1(14)
Li2	Li3	Li2	111.9(13)	S2	Li4	S2	82.8(14)
Li2	Li3	Zn1	120.60(14)	S2	Li4	S3	112.9(14)
Li2	Li4	Li1	25.8(10)	S2	Li4	Zn1	138.3(6)
Li2	Li4	Li2	128(4)	S2	Si1	S2	109.471
Li2	Li4	Zn1	25.8(10)	S2	Zn1	Li1	102.51(7)
Li2	S1	Li2	90	S2	Zn1	Li2	64(2)
Li2	S2	Li2	109.3(14)	S2	Zn1	Li3	72.2(5)
Li2	S2	Si1	109.6(14)	S2	Zn1	Li4	81(2)
Li2	Zn1	Li2	110(4)	S2	Zn1	S2	94.97(13)
Li2	Zn1	Li3	124.0(11)	S2	Zn1	Zn1	102.51(7)
Li2	Zn1	Li4	71.3(10)	S3	Li1	Li1	62.84(7)
Li2	Zn1	S1	51.3(10)	S3	Li1	Li2	171(4)
Li2	Zn1	S2	96.3(4)	S3	Li1	Li3	58.0(8)
Li2	Zn1	S3	77.8(7)	S3	Li1	Li4	55.7(11)
Li2	Zn1	Zn1	97.7(9)	S3	Li1	S1	115.40(14)
Li3	Li1	Li1	96.0(3)	S3	Li1	S2	110.41(10)
Li3	Li1	Li2	113(4)	S3	Li1	Zn1	62.84(7)
Li3	Li1	Li3	90.3(12)	S3	Li3	Li1	83.3(8)
Li3	Li1	Li4	101(2)	S3	Li3	Li2	106.9(15)
Li3	Li1	S1	96.3(4)	S3	Li3	Zn1	83.3(8)
Li3	Li1	S2	53.05(16)	S3	Li4	Li1	85(2)
Li3	Li1	S3	148.3(4)	S3	Li4	Li2	110.0(17)
Li3	Li2	Li1	44(2)	S3	Li4	Li3	56.8(11)
Li3	Li2	Li3	104(3)	S3	Li4	Li4	63.9(11)
Li3	Li2	Li4	66.6(14)	S3	Li4	Zn1	85(2)
Li3	Li2	Zn1	44(2)	S3	Zn1	Li1	62.84(7)
Li3	Li4	Li1	141(3)	S3	Zn1	Li2	171(4)
Li3	Li4	Li2	62.4(16)	S3	Zn1	Li3	58.0(8)
Li3	Li4	Li3	106(2)	S3	Zn1	Li4	55.7(11)
Li3	Li4	Li4	62.3(10)	S3	Zn1	S1	115.40(14)
Li3	Li4	Zn1	141(3)	S3	Zn1	S2	110.41(10)
Li3	S2	Li1	38.4(2)	S3	Zn1	Zn1	62.84(7)
Li3	S2	Li2	54.7(7)	Si1	Li4	Li1	78.3(11)
Li3	S2	Li3	87.0(7)	Si1	Li4	Li2	87.8(15)
Li3	S2	Si1	127.3(4)	Si1	Li4	Li3	101.4(14)
Li3	S2	Zn1	122.0(4)	Si1	Li4	Li4	60.6(7)
Li3	S3	Li3	109.471	Si1	Li4	S1	102.0(15)
Li3	Zn1	Li2	113(4)	Si1	Li4	S2	42.9(7)
Li3	Zn1	Li3	90.3(12)	Si1	Li4	S3	133.6(17)
Li3	Zn1	Li4	101(2)	Si1	Li4	Zn1	78.3(11)

Li3	Zn1	S1	96.3(4)	Zn1	Li1	Li2	6(4)
Li3	Zn1	S2	53.05(16)	Zn1	Li1	Li3	118.6(8)
Li3	Zn1	S3	148.3(4)	Zn1	Li1	Li4	116(2)
Li3	Zn1	Zn1	96.0(3)	Zn1	Li1	S1	147.43(3)
Li4	Li1	Li1	30.10(7)	Zn1	Li1	S2	99.57(8)
Li4	Li1	Li2	118(3)	Zn1	Li1	S3	57.20(6)
Li4	Li1	Li3	113.7(13)	Zn1	Li2	Li3	156.76(7)
Li4	Li1	Li4	102(4)	Zn1	Li2	Li4	56.2(17)
Li4	Li1	S1	57.3(9)	Zn1	Li2	S1	54.0(10)
Li4	Li1	S2	132.43(9)	Zn1	Li2	S2	90.9(3)
Li4	Li1	S3	156.3(9)	Zn1	Li2	Zn1	169(7)
Li4	Li2	Li1	138(4)	Zn1	Li3	Li1	118.7(4)
Li4	Li2	Li4	102(3)	Zn1	Li3	Li2	107.4(9)
Li4	Li2	Zn1	138(4)	Zn1	Li3	Li4	101.9(12)
Li4	Li3	Li1	88.0(11)	Zn1	Li3	S2	51.8(2)
Li4	Li3	Li2	98.1(13)	Zn1	Li3	S3	116.5(4)
Li4	Li3	Li4	129(2)	Zn1	Li3	Zn1	118.7(4)
Li4	Li3	S3	64.4(12)	Zn1	Li4	Li1	137(4)
Li4	Li3	Zn1	88.0(11)	Zn1	Li4	Li2	84.5(16)
Li4	Li4	Li1	99(2)	Zn1	Li4	Li3	89.0(14)
Li4	Li4	Li2	58.9(15)	Zn1	Li4	Li4	127.8(11)
Li4	Li4	Li3	95.9(11)	Zn1	Li4	S1	60.4(11)
Li4	Li4	Li4	60	Zn1	Li4	S2	118.5(17)
Li4	Li4	S1	53.4(9)	Zn1	Li4	S3	63.9(12)
Li4	Li4	S2	95.0(9)	Zn1	Li4	Zn1	137(4)
Li4	Li4	S3	148.8(2)	Zn1	S1	Li2	158.06(8)
Li4	Li4	Zn1	99(2)	Zn1	S1	Li4	64.7(4)
Li4	S1	Li2	45.58(17)	Zn1	S1	Zn1	114.85(7)
Li4	S1	Li4	73.1(17)	Zn1	S2	Li2	129.3(9)
Li4	S2	Li1	133.2(9)	Zn1	S2	Si1	106.28(7)
Li4	S2	Li2	154.7(10)	Zn1	S2	Zn1	69.61(17)
Li4	S2	Li3	52.6(9)	Zn1	S3	Li1	65.61(13)
Li4	S2	Li4	70.2(19)	Zn1	S3	Li3	88.00(5)
Li4	S2	Si1	79.9(7)	Zn1	S3	Li4	66.7(9)
Li4	S2	Zn1	109.7(10)	Zn1	S3	Zn1	65.61(13)
Li4	S3	Li3	99.1(11)	Zn1	Zn1	Li2	6(4)
Li4	S3	Li4	117.5(5)	Zn1	Zn1	Li3	118.6(8)
Li4	Si1	Li4	113.5(6)	Zn1	Zn1	Li4	116(2)
Li4	Si1	S2	105.1(8)	Zn1	Zn1	S1	147.43(3)
Li4	Zn1	Li2	118(3)	Zn1	Zn1	S2	99.57(8)
Li4	Zn1	Li3	113.7(13)	Zn1	Zn1	S3	57.20(6)
Li4	Zn1	Li4	102(4)	Zn1	Zn1	Zn1	120
Li4	Zn1	S1	57.3(9)				

Table S5. Bond distances obtained from Rietveld refinement against SXRD and NPD data of the room temperature $I\bar{4}$ structure (T = 300 K) of $Li_7Zn_{0.5}SiS_6$.

Site 1	Site 2	Distance (Å)	Site 1	Site 2	Distance (Å)
Li16	Li17	1.3(4)	Li12	S08	2.49(3)
Zn6	Li17	1.3(4)	S06	Li21	2.49(4)
Li17	Li16	1.3(4)	Li6	Li16	2.49(4)
Li14	Li15	1.82(7)	Li6	Zn6	2.49(4)
Zn4	Zn5	1.98(5)	Li16	Li6	2.49(4)
Zn4	Li10	1.98(5)	Zn6	Li6	2.49(4)
Li4	Zn5	1.98(5)	Li21	S06	2.49(4)
Li4	Li10	1.98(5)	S03	Li17	2.5(4)
Li10	Zn4	1.98(5)	S07	Li17	2.5(4)
Zn5	Li4	1.98(5)	S08	Li17	2.5(4)
Zn5	Zn4	1.98(5)	S14	Li17	2.5(4)
S12	Si2	2.060(9)	Li17	S03	2.5(4)
S07	Si2	2.089(9)	Li17	Li21	2.5(4)
Si2	Li20	2.1(2)	Li17	S07	2.5(4)
S06	Si1	2.104(8)	Li17	S08	2.5(4)
S05	Si1	2.108(9)	Li21	Li17	2.5(4)
S04	Si1	2.117(9)	S04	Li11	2.50(3)
S11	Si2	2.128(9)	S06	Li2	2.505(15)
S13	Si3	2.138(6)	S06	Zn2	2.505(15)
S10	Si1	2.143(9)	Li2	S06	2.505(15)
S03	Si2	2.191(9)	Li5	Li8	2.51(5)
Li20	Li21	2.2(2)	S01	Li5	2.53(3)
Li19	Li20	2.2(3)	S11	Li7	2.53(4)
Li19	Li22	2.2(4)	S11	Li14	2.53(4)
Li7	Li10	2.22(7)	Li7	S11	2.53(4)
Li7	Zn5	2.22(7)	Li14	S11	2.53(4)
Li10	Li7	2.22(7)	S08	Li6	2.54(3)
Zn5	Li7	2.22(7)	S13	Li13	2.54(3)
S15	Li15	2.3(2)	Li6	S08	2.54(3)
Li13	Li22	2.3(4)	Li13	S13	2.54(3)
Li18	Li22	2.3(4)	S14	Zn5	2.54(5)
Li22	Li13	2.3(4)	S14	Li10	2.54(5)
S13	Li15	2.33(15)	Zn5	S14	2.54(5)
S05	Li1	2.378(8)	S05	Li11	2.55(3)
S05	Zn1	2.378(8)	S07	Li9	2.55(3)
Li1	S05	2.378(8)	Li11	S05	2.55(3)
Li10	Li11	2.39(6)	S08	Li14	2.55(4)
Zn5	Li11	2.39(6)	S09	Li7	2.55(4)
Li11	Li10	2.39(6)	Zn1	Li21	2.55(4)
Li11	Zn5	2.39(6)	Li1	Li21	2.55(4)
S04	Zn2	2.397(14)	Li7	S09	2.55(4)
S04	Li2	2.397(14)	Li14	S08	2.55(4)

Zn2	S04	2.397(14)	Li21	Zn1	2.55(4)
Li2	S04	2.397(14)	Li21	Li1	2.55(4)
S03	Li20	2.4(2)	S04	Li10	2.55(5)
S07	Li20	2.4(2)	S04	Zn5	2.55(5)
S09	Li20	2.4(2)	S09	Li10	2.55(5)
S12	Li20	2.4(2)	S09	Zn5	2.55(5)
Li20	S09	2.4(2)	Li10	S09	2.55(5)
Li20	S12	2.4(2)	Li10	S04	2.55(5)
Li20	S07	2.4(2)	Zn5	S09	2.55(5)
Li20	S03	2.4(2)	Zn5	S04	2.55(5)
Zn3	Li20	2.4(3)	Zn3	Li4	2.554(19)
Li3	Li20	2.4(3)	Zn3	Zn4	2.554(19)
Li20	Zn3	2.4(3)	Li3	Li4	2.554(19)
Li8	Li17	2.4(4)	Li3	Zn4	2.554(19)
S13	Li5	2.40(3)	Zn4	Zn3	2.554(19)
S09	Li4	2.408(16)	Li4	Li3	2.554(19)
S09	Zn4	2.408(16)	Li4	Zn3	2.554(19)
Li4	S09	2.408(16)	S03	Li18	2.56(3)
S09	Li1	2.409(7)	S06	Li6	2.56(4)
S09	Zn1	2.409(7)	S14	Zn6	2.562(19)
Li1	S09	2.409(7)	S14	Li16	2.562(19)
S01	Li13	2.41(3)	Zn6	S14	2.562(19)
S15	Li12	2.41(3)	S01	Li8	2.57(3)
Li13	S01	2.41(3)	S12	Li19	2.57(4)
S10	Zn3	2.411(14)	S14	Li2	2.576(14)
S10	Li3	2.411(14)	S14	Zn2	2.576(14)
Zn3	S10	2.411(14)	Li2	S14	2.576(14)
Li3	S10	2.411(14)	S14	Li7	2.58(4)
S04	Li4	2.411(15)	S15	Li14	2.58(4)
S04	Zn4	2.411(15)	Li7	S14	2.58(4)
Li4	S04	2.411(15)	Li14	S15	2.58(4)
S07	Zn3	2.414(14)	S12	Zn5	2.58(5)
S07	Li3	2.414(14)	S12	Li10	2.58(5)
Zn3	S07	2.414(14)	Zn5	S12	2.58(5)
Li3	S07	2.414(14)	S09	Li19	2.59(4)
S09	Li3	2.418(13)	S01	Li3	2.596(14)
S09	Zn3	2.418(13)	S01	Zn3	2.596(14)
Li3	S09	2.418(13)	Li3	S01	2.596(14)
S01	Li18	2.42(3)	S01	Li22	2.6(4)
S10	Li13	2.43(3)	S06	Li22	2.6(4)
S08	Li8	2.44(3)	S09	Li22	2.6(4)
S11	Li12	2.44(3)	S10	Li22	2.6(4)
Li8	S08	2.44(3)	Li22	S06	2.6(4)
Li12	S11	2.44(3)	Li22	S01	2.6(4)
S10	Li7	2.44(4)	Li22	S09	2.6(4)
S07	Li16	2.45(2)	S14	Li6	2.60(4)

S07	Zn6	2.45(2)	S01	Li9	2.61(3)
S11	Zn6	2.45(2)	S01	Li19	2.61(4)
S11	Li16	2.45(2)	Zn2	Li11	2.64(3)
Li16	S07	2.45(2)	Li2	Li11	2.64(3)
Zn6	S11	2.45(2)	Li11	Zn2	2.64(3)
Zn6	S07	2.45(2)	Li12	Li14	2.66(5)
S03	Li8	2.45(3)	S02	Li11	2.68(3)
S06	Li18	2.45(3)	Li12	Zn6	2.69(4)
S08	Li9	2.45(3)	Li12	Li16	2.69(4)
Li8	S03	2.45(3)	Zn6	Li12	2.69(4)
Li9	S08	2.45(3)	Si3	Li15	2.7(2)
Li18	S06	2.45(3)	Li21	Li22	2.7(4)
S10	Li5	2.46(3)	Li6	Li10	2.78(6)
S14	Li21	2.46(3)	Li6	Zn5	2.78(6)
Li5	S10	2.46(3)	Li10	Li6	2.78(6)
Li21	S14	2.46(3)	Zn5	Li6	2.78(6)
S12	Zn4	2.461(15)	Li6	Li17	2.8(4)
S12	Li4	2.461(15)	Li7	Li22	2.8(4)
Zn4	S12	2.461(15)	Li17	Li6	2.8(4)
Li4	S12	2.461(15)	Li22	Li7	2.8(4)
S04	Li8	2.47(3)	Si2	Li12	2.88(3)
S11	Li13	2.47(3)	Si2	Li17	2.9(4)
S14	Li11	2.47(3)	Li12	Li15	2.90(15)
Li8	S04	2.47(3)	Li7	Zn6	2.93(5)
Li11	S14	2.47(3)	Li7	Li16	2.93(5)
Li13	S11	2.47(3)	Li9	Li12	2.93(5)
S03	Li19	2.47(4)	Li16	Li7	2.93(5)
S03	Li21	2.47(4)	Zn6	Li7	2.93(5)
S12	Li6	2.47(4)	Li5	Li13	2.98(4)
S13	Li14	2.47(4)	Li13	Li14	2.98(5)
Li6	S12	2.47(4)	Zn3	Li9	2.99(3)
Li14	S13	2.47(4)	Zn3	Li5	2.99(3)
Li19	S03	2.47(4)	Li3	Li9	2.99(3)
Li21	S03	2.47(4)	Li3	Li5	2.99(3)
S14	Li1	2.477(8)	Li5	Zn3	2.99(3)
S14	Zn1	2.477(8)	Li9	Li3	2.99(3)
Li1	S14	2.477(8)	Li9	Zn3	2.99(3)
S12	Li18	2.48(3)	Zn4	Li8	2.99(4)
S09	Li21	2.48(4)	Li4	Li8	2.99(4)
S01	Zn4	2.481(14)	Li8	Zn4	2.99(4)
S01	Li4	2.481(14)	Li8	Li4	2.99(4)
Zn4	S01	2.481(14)	Li17	Li20	3.0(5)
Li4	S01	2.481(14)	Si1	Li11	3.00(3)
S02	Zn2	2.482(13)	Zn4	Li19	3.00(4)
S02	Li2	2.482(13)	Li4	Li19	3.00(4)
Zn2	S02	2.482(13)	Li19	Zn4	3.00(4)

Li2	S02	2.482(13)	Li8	Li19	3.05(5)
S08	Li16	2.49(2)	Li5	Li9	3.06(5)
S08	Zn6	2.49(2)	Zn2	Li21	3.08(4)
Li16	S08	2.49(2)	Li2	Li21	3.08(4)
Zn6	S08	2.49(2)	Li21	Zn2	3.08(4)
S07	Li12	2.49(3)	Li6	Li18	3.08(5)
S08	Li5	2.49(3)	Si1	Li22	3.1(4)
S08	Li12	2.49(3)	Li9	Li18	3.10(5)
S13	Li9	2.49(3)	Li18	Li21	3.13(5)
Li5	S08	2.49(3)	Li7	Li13	3.16(5)
Li9	S13	2.49(3)	Li18	Li19	3.16(5)
Li12	S07	2.49(3)	Li20	Li22	3.2(4)

Table S6. Angles obtained from Rietveld refinement against SXRD and NPD data of the room temperature $I\bar{4}$ structure (T = 300 K) of $Li_7Zn_{0.5}SiS_6$

Site 1	Site 2	Site 3	Angle at site 2 (°)	Site 1	Site 2	Site 3	Angle at site 2 (°)
Li1	Li21	Li17	110(10)	Li6	Zn5	Li7	94(2)
Li1	Li21	Li20	110(6)	Li6	Zn5	S04	99.9(19)
Li1	Li21	S03	167.7(17)	Li6	Zn5	S09	150(2)
Li1	Li21	S06	100.3(12)	Li6	Zn5	S12	54.9(13)
Li1	Li21	S09	57.2(8)	Li6	Zn5	S14	58.4(13)
Li1	Li21	S14	59.1(8)	Li6	Zn5	Zn4	116(2)
Li1	S05	Li1	113.1(2)	Li6	Zn6	Li17	87(19)
Li1	S05	Si1	103.2(3)	Li6	Zn6	S07	165.3(12)
Li1	S05	Zn1	113.1(2)	Li6	Zn6	S08	61.3(10)
Li1	S09	Li20	108(6)	Li6	Zn6	S11	99.7(11)
Li1	S09	Li4	114.7(4)	Li7	Li10	Li4	116(3)
Li1	S09	Zn4	114.7(4)	Li7	Li10	Zn4	116(3)
Li1	S14	Li11	92.9(7)	Li7	Li13	Li14	104.3(14)
Li1	S14	Li21	62.3(9)	Li7	Li13	Li22	59(9)
Li10	Li6	Li16	89.4(17)	Li7	Li13	Li5	101.6(13)
Li10	Li6	Li17	104(9)	Li7	Li13	S01	119.8(13)
Li10	Li6	S06	99.8(15)	Li7	Li13	S10	49.7(9)
Li10	Li6	S08	143.8(19)	Li7	Li13	S11	51.5(10)
Li10	Li6	S12	58.4(13)	Li7	Li13	S13	119.4(14)
Li10	Li6	S14	56.2(12)	Li7	Li16	Li12	110.5(12)
Li10	Li6	Zn6	89.4(17)	Li7	Li16	Li17	124(19)
Li10	S04	Li11	56.6(13)	Li7	Li16	Li6	85.0(13)
Li10	S04	Li2	108.7(12)	Li7	Li16	S07	104.5(11)
Li10	S04	Li4	46.9(11)	Li7	Li16	S08	140.4(11)
Li10	S04	Li8	119.1(13)	Li7	Li16	S11	55.1(9)
Li10	S04	Si1	105.0(12)	Li7	Li16	S14	55.6(9)
Li10	S04	Zn2	108.7(12)	Li7	Li22	Li13	76(10)
Li10	S04	Zn4	46.9(11)	Li7	Li22	Li18	172(18)
Li10	S09	Li1	92.0(12)	Li7	Li22	Li19	94(12)

Li10	S09	Li20	154(5)	Li7	Li22	Li21	102(12)
Li10	S09	Li21	152.5(14)	Li7	Li22	S01	127(14)
Li10	S09	Li3	104.2(11)	Li7	Li22	S06	113(13)
Li10	S09	Li4	47.0(11)	Li7	Li22	S09	56(7)
Li10	S09	Zn1	92.0(12)	Li7	Li22	S10	54(7)
Li10	S09	Zn3	104.2(11)	Li7	S09	Li1	94.5(9)
Li10	S09	Zn4	47.0(11)	Li7	S09	Li10	51.6(15)
Li10	S12	Li18	125.6(14)	Li7	S09	Li20	140(6)
Li10	S12	Li19	118.3(14)	Li7	S09	Li21	115.4(13)
Li10	S12	Li20	144(6)	Li7	S09	Li3	155.3(10)
Li10	S12	Li4	46.2(11)	Li7	S09	Li4	92.0(10)
Li10	S12	Li6	66.8(13)	Li7	S09	Zn1	94.5(9)
Li10	S12	Si2	105.4(13)	Li7	S09	Zn3	155.3(10)
Li10	S12	Zn4	46.2(11)	Li7	S09	Zn4	92.0(10)
Li10	S14	Li1	111.7(12)	Li7	S09	Zn5	51.6(15)
Li10	S14	Li11	57.1(14)	Li7	S10	Li13	81.0(12)
Li10	S14	Li17	120(10)	Li7	S10	Li3	106.2(10)
Li10	S14	Li21	172.0(15)	Li7	S10	Si1	102.4(10)
Li10	S14	Zn1	111.7(12)	Li7	S10	Zn3	106.2(10)
Li11	Li10	Li4	122(3)	Li7	S11	Li12	136.9(12)
Li11	Li10	Li7	114(2)	Li7	S11	Li13	78.5(12)
Li11	Li10	Zn4	122(3)	Li7	S11	Li16	72.1(10)
Li11	Li2	Li11	119.2(12)	Li7	S11	Si2	102.7(9)
Li11	Li2	S02	63.1(7)	Li7	S11	Zn6	72.1(10)
Li11	Li2	S04	134.2(9)	Li7	S14	Li1	84.4(9)
Li11	Li2	S06	128.1(8)	Li7	S14	Li10	51.3(15)
Li11	Li2	S14	56.6(7)	Li7	S14	Li11	100.2(11)
Li11	S02	Li11	121.7(7)	Li7	S14	Li16	69.5(10)
Li11	S02	Li2	96.9(7)	Li7	S14	Li17	98(10)
Li11	S02	Zn2	96.9(7)	Li7	S14	Li2	162.5(9)
Li11	S04	Li2	69.6(8)	Li7	S14	Li21	121.3(12)
Li11	S04	Li4	102.3(8)	Li7	S14	Zn1	84.4(9)
Li11	S04	Li8	171.0(11)	Li7	S14	Zn2	162.5(9)
Li11	S04	Si1	80.4(7)	Li7	S14	Zn5	51.3(15)
Li11	S04	Zn2	69.6(8)	Li7	S14	Zn6	69.5(10)
Li11	S04	Zn4	102.3(8)	Li7	Zn5	Li4	116(3)
Li11	S05	Li1	137.9(7)	Li7	Zn5	Zn4	116(3)
Li11	S05	Si1	79.4(7)	Li7	Zn6	Li12	110.5(12)
Li11	S05	Zn1	137.9(7)	Li7	Zn6	Li17	124(19)
Li11	S14	Li21	126.4(11)	Li7	Zn6	Li6	85.0(13)
Li11	Si1	S04	55.4(6)	Li7	Zn6	S07	104.5(11)
Li11	Si1	S05	56.9(6)	Li7	Zn6	S08	140.4(11)
Li11	Si1	S06	109.9(6)	Li7	Zn6	S11	55.1(9)
Li11	Si1	S10	141.7(6)	Li7	Zn6	S14	55.6(9)
Li11	Zn2	Li11	119.2(12)	Li8	Li17	Li16	130(30)
Li11	Zn2	S02	63.1(7)	Li8	Li17	Zn6	130(30)

Li11	Zn2	S04	134.2(9)
Li11	Zn2	S06	128.1(8)
Li11	Zn2	S14	56.6(7)
Li11	Zn5	Li4	122(3)
Li11	Zn5	Li7	114(2)
Li11	Zn5	Zn4	122(3)
Li12	Li14	Li12	82.5(14)
Li12	Li14	Li15	78(4)
Li12	Li14	S08	57.1(11)
Li12	Li14	S11	127.7(18)
Li12	Li14	S13	138.4(18)
Li12	Li14	S15	54.8(11)
Li12	Li15	Li12	107(7)
Li12	Li15	Li14	64(4)
Li12	Li15	S13	95.4(8)
Li12	Li15	S15	54(4)
Li12	Li15	Si3	126(4)
Li12	Li16	Li17	130(20)
Li12	Li16	Li6	95.3(12)
Li12	Li16	S07	91.8(9)
Li12	Li16	S08	57.5(8)
Li12	Li16	S11	56.5(8)
Li12	Li16	S14	152.5(11)
Li12	Li9	S01	154.5(14)
Li12	Li9	S07	53.4(9)
Li12	Li9	S08	54.3(9)
Li12	Li9	S13	91.4(12)
Li12	S07	Li16	109.2(9)
Li12	S07	Li20	129(6)
Li12	S07	Li3	145.3(8)
Li12	S07	Si2	77.6(8)
Li12	S07	Zn3	145.3(8)
Li12	S07	Zn6	109.2(9)
Li12	S08	Li16	65.4(9)
Li12	S08	Li5	144.7(11)
Li12	S08	Li8	143.3(11)
Li12	S08	Li9	72.6(11)
Li12	S08	Zn6	65.4(9)
Li12	S11	Si2	77.8(8)
Li12	S15	Li12	93.5(3)
Li12	S15	Li15	104.3(8)
Li12	Si2	Li20	126(7)
Li12	Si2	S03	142.3(7)
Li12	Si2	S07	57.4(7)
Li12	Si2	S11	56.0(7)
Li12	Si2	S12	110.6(7)
Li8	Li19	Li20	152(6)
Li8	Li19	Li22	108(10)
Li8	Li19	Li4	59.2(10)
Li8	Li19	S01	53.3(10)
Li8	Li19	S03	51.5(10)
Li8	Li19	S09	147.1(17)
Li8	Li19	S12	92.7(13)
Li8	Li19	Zn4	59.2(10)
Li8	Li4	Li10	120.0(17)
Li8	Li4	Li3	92.7(8)
Li8	Li4	S01	55.0(7)
Li8	Li4	S04	53.3(7)
Li8	Li4	S09	147.2(8)
Li8	Li4	S12	96.4(8)
Li8	Li4	Zn3	92.7(8)
Li8	Li4	Zn5	120.0(17)
Li8	Li5	S08	58.4(11)
Li8	Li5	S10	105.4(15)
Li8	Li5	S13	159.5(17)
Li8	S01	Li13	87.9(11)
Li8	S01	Li18	153.0(11)
Li8	S01	Li4	72.6(8)
Li8	S01	Li5	58.9(11)
Li8	S01	Zn4	72.6(8)
Li8	S03	Li20	133(7)
Li8	S03	Si2	106.6(8)
Li8	S04	Li2	106.8(9)
Li8	S04	Li4	75.4(9)
Li8	S04	Si1	108.6(8)
Li8	S04	Zn2	106.8(9)
Li8	S04	Zn4	75.4(9)
Li8	Zn4	Li10	120.0(17)
Li8	Zn4	Li3	92.7(8)
Li8	Zn4	S01	55.0(7)
Li8	Zn4	S04	53.3(7)
Li8	Zn4	S09	147.2(8)
Li8	Zn4	S12	96.4(8)
Li8	Zn4	Zn3	92.7(8)
Li8	Zn4	Zn5	120.0(17)
Li9	Li12	Li14	150.6(17)
Li9	Li12	Li15	71(4)
Li9	Li12	Li16	101.9(13)
Li9	Li12	S07	55.3(9)
Li9	Li12	S08	53.0(9)
Li9	Li12	S11	120.7(14)
Li9	Li12	S15	119.6(14)

Li12	Zn6	Li17	130(20)
Li12	Zn6	Li6	95.3(12)
Li12	Zn6	S07	91.8(9)
Li12	Zn6	S08	57.5(8)
Li12	Zn6	S11	56.5(8)
Li12	Zn6	S14	152.5(11)
Li13	Li14	Li12	108.1(15)
Li13	Li14	Li15	118(6)
Li13	Li14	S08	96.1(14)
Li13	Li14	S11	52.6(9)
Li13	Li14	S13	54.7(10)
Li13	Li14	S15	151.4(17)
Li13	Li22	Li18	104(15)
Li13	Li22	Li19	93(14)
Li13	Li5	Li8	144.6(16)
Li13	Li5	S01	142.4(14)
Li13	Li5	S08	105.4(12)
Li13	Li5	S10	52.0(8)
Li13	Li5	S13	55.1(9)
Li13	Li7	Li10	143(2)
Li13	Li7	Li16	91.6(12)
Li13	Li7	Li22	45(8)
Li13	Li7	S09	97.9(13)
Li13	Li7	S10	49.3(9)
Li13	Li7	S11	50.0(9)
Li13	Li7	S14	142.2(15)
Li13	Li7	Zn5	143(2)
Li13	Li7	Zn6	91.6(12)
Li13	S10	Li3	121.5(8)
Li13	S10	Si1	129.4(8)
Li13	S10	Zn3	121.5(8)
Li13	S11	Li12	136.8(11)
Li13	S11	Li16	125.1(9)
Li13	S11	Si2	125.1(8)
Li13	S11	Zn6	125.1(9)
Li13	S13	Li14	72.9(12)
Li13	S13	Li15	117(2)
Li13	S13	Li5	74.0(10)
Li13	S13	Li9	128.9(10)
Li13	S13	Si3	124.5(8)
Li14	Li12	Li14	116.0(18)
Li14	Li12	S07	129.9(16)
Li14	Li12	S08	59.2(11)
Li14	Li12	S11	140.5(16)
Li14	Li12	S15	60.9(11)
Li14	Li13	Li22	154(9)
Li9	Li12	Si2	79.3(10)
Li9	Li12	Zn6	101.9(13)
Li9	Li18	Li22	94(9)
Li9	Li18	Li6	73.0(11)
Li9	Li18	S01	54.6(9)
Li9	Li18	S03	157.5(14)
Li9	Li18	S06	111.3(12)
Li9	Li18	S12	84.6(11)
Li9	Li3	Li20	93(5)
Li9	Li3	Li4	111.9(8)
Li9	Li3	S01	55.1(7)
Li9	Li3	S07	55.0(7)
Li9	Li3	S09	142.6(8)
Li9	Li3	S10	102.8(8)
Li9	Li3	Zn4	111.9(8)
Li9	Li5	Li13	97.4(13)
Li9	Li5	Li3	59.2(9)
Li9	Li5	Li8	114.6(14)
Li9	Li5	S01	54.5(9)
Li9	Li5	S08	141.0(15)
Li9	Li5	S10	99.6(12)
Li9	Li5	S13	52.5(9)
Li9	Li5	Zn3	59.2(9)
Li9	S01	Li13	79.7(11)
Li9	S01	Li18	76.2(11)
Li9	S01	Li3	70.2(8)
Li9	S01	Li4	129.5(8)
Li9	S01	Li5	73.2(11)
Li9	S01	Li8	130.7(11)
Li9	S01	Zn3	70.2(8)
Li9	S01	Zn4	129.5(8)
Li9	S07	Li12	71.2(10)
Li9	S07	Li16	147.8(10)
Li9	S07	Li17	175(9)
Li9	S07	Li20	104(6)
Li9	S07	Li3	74.1(8)
Li9	S07	Si2	105.9(8)
Li9	S07	Zn3	74.1(8)
Li9	S07	Zn6	147.8(10)
Li9	S08	Li8	143.3(12)
Li9	S13	Li14	114.3(13)
Li9	S13	Li15	88.8(14)
Li9	S13	Li5	77.6(11)
Li9	S13	Si3	103.9(8)
Li9	Zn3	Li20	93(5)
Li9	Zn3	Li4	111.9(8)

Li14	Li13	S01	111.9(13)
Li14	Li13	S10	120.0(14)
Li14	Li13	S11	54.4(10)
Li14	Li13	S13	52.4(10)
Li14	Li15	Li14	153(14)
Li14	S08	Li12	63.7(12)
Li14	S08	Li16	96.7(11)
Li14	S08	Li17	96(10)
Li14	S08	Li5	94.0(12)
Li14	S08	Li6	155.9(13)
Li14	S08	Li8	97.3(12)
Li14	S08	Li9	95.8(12)
Li14	S08	Zn6	96.7(11)
Li14	S11	Li12	64.8(12)
Li14	S11	Li13	73.0(12)
Li14	S11	Li16	111.6(10)
Li14	S11	Li7	146.9(13)
Li14	S11	Si2	107.2(10)
Li14	S11	Zn6	111.6(10)
Li14	S13	Li15	44(3)
Li14	S13	Li5	144.3(12)
Li14	S13	Si3	102.8(9)
Li14	S15	Li12	94.1(11)
Li14	S15	Li14	86.2(18)
Li14	S15	Li15	136.9(9)
Li15	Li12	Li14	110(4)
Li15	Li12	Li16	126.5(16)
Li15	Li12	S07	97.1(19)
Li15	Li12	S08	81(3)
Li15	Li12	S11	168(4)
Li15	Li12	S15	51(4)
Li15	Li12	Si2	142(2)
Li15	Li12	Zn6	126.5(16)
Li15	S13	Si3	75(5)
Li15	S15	Li15	180
Li15	Si3	Li15	180.0000(10)
Li15	Si3	S13	124.03(17)
Li16	Li12	Li14	100.6(14)
Li16	Li12	S07	122.7(14)
Li16	Li12	S08	57.2(9)
Li16	Li12	S11	56.8(9)
Li16	Li12	S15	122.5(13)
Li16	Li6	S12	103.4(14)
Li16	Li7	Li10	91.2(19)
Li16	Li7	Li22	136(8)
Li16	Li7	S09	149.5(17)

Li9	Zn3	S01	55.1(7)
Li9	Zn3	S07	55.0(7)
Li9	Zn3	S09	142.6(8)
Li9	Zn3	S10	102.8(8)
Li9	Zn3	Zn4	111.9(8)
S01	Li13	Li22	68(9)
S01	Li18	Li22	68(9)
S01	Li19	Li20	123(6)
S01	Li19	Li22	65(9)
S01	Li19	S03	103.9(14)
S01	Li19	S09	130.9(15)
S01	Li19	S12	103.3(14)
S01	Li22	Li13	58(9)
S01	Li22	Li18	59(8)
S01	Li22	Li19	64(9)
S01	Li22	S06	115(13)
S01	Li22	S10	113(14)
S01	Li3	Li20	112(6)
S01	Li3	Li4	57.6(4)
S01	Li3	S07	108.7(5)
S01	Li3	S09	109.0(5)
S01	Li3	S10	103.4(5)
S01	Li3	Zn4	57.6(4)
S01	Li4	Li10	175.0(17)
S01	Li4	S04	104.8(5)
S01	Li4	S09	113.2(6)
S01	Li4	S12	110.6(5)
S01	Li4	Zn5	175.0(17)
S01	Li5	Li8	61.2(10)
S01	Li5	S08	112.0(12)
S01	Li5	S10	103.8(12)
S01	Li5	S13	106.7(12)
S01	Li8	Li17	155(11)
S01	Li8	Li5	59.9(11)
S01	Li8	S03	105.6(12)
S01	Li8	S04	100.5(12)
S01	Li8	S08	112.5(13)
S01	Li9	S07	104.4(10)
S01	Li9	S08	134.4(15)
S01	Li9	S13	102.0(12)
S01	Zn3	Li20	112(6)
S01	Zn3	Li4	57.6(4)
S01	Zn3	S07	108.7(5)
S01	Zn3	S09	109.0(5)
S01	Zn3	S10	103.4(5)
S01	Zn3	Zn4	57.6(4)

Li16	Li7	S10	95.5(13)
Li16	Li7	S11	52.8(8)
Li16	Li7	S14	54.9(9)
Li16	Li7	Zn5	91.2(19)
Li16	S07	Li20	100(6)
Li16	S07	Li3	100.9(6)
Li16	S07	Si2	105.5(6)
Li16	S07	Zn3	100.9(6)
Li16	S08	Li5	148.5(9)
Li16	S08	Li8	87.9(10)
Li16	S08	Li9	124.3(10)
Li16	S11	Li12	66.7(9)
Li16	S11	Si2	106.3(6)
Li16	S14	Li1	119.1(6)
Li16	S14	Li10	93.4(13)
Li16	S14	Li11	143.9(9)
Li16	S14	Li17	30(10)
Li16	S14	Li21	85.7(9)
Li16	S14	Zn1	119.1(6)
Li16	S14	Zn5	93.4(13)
Li17	Li20	Li19	152(13)
Li17	Li20	Li21	56(10)
Li17	Li20	Li3	90(12)
Li17	Li20	S03	54(10)
Li17	Li20	S07	55(10)
Li17	Li20	S09	110(12)
Li17	Li20	S12	121(11)
Li17	Li20	Si2	67(11)
Li17	Li20	Zn3	90(12)
Li17	Li21	Li20	79(12)
Li17	Li21	S03	60(10)
Li17	Li21	S06	124(10)
Li17	Li21	S09	124(10)
Li17	Li21	S14	60(10)
Li17	Li6	Li16	28(9)
Li17	Li6	S06	139(9)
Li17	Li6	S08	57(9)
Li17	Li6	S12	132(9)
Li17	Li6	S14	55(9)
Li17	Li6	Zn6	28(9)
Li17	S03	Li19	134(10)
Li17	S03	Li20	75(12)
Li17	S03	Li21	61(10)
Li17	S03	Li8	59(10)
Li17	S03	Si2	76(10)
Li17	S07	Li12	113(10)
S01	Zn4	Li10	175.0(17)
S01	Zn4	S04	104.8(5)
S01	Zn4	S09	113.2(6)
S01	Zn4	S12	110.6(5)
S01	Zn4	Zn5	175.0(17)
S02	Li11	Li10	151.9(18)
S02	Li11	Li2	55.6(6)
S02	Li11	S04	102.5(10)
S02	Li11	S05	105.9(11)
S02	Li11	S14	116.0(11)
S02	Li11	Zn2	55.6(6)
S02	Li11	Zn5	151.9(18)
S02	Li2	S04	112.1(5)
S02	Zn2	S04	112.1(5)
S03	Li17	Li16	160(30)
S03	Li17	Li8	60(10)
S03	Li17	S14	114(16)
S03	Li17	Zn6	160(30)
S03	Li18	Li22	99(9)
S03	Li18	S01	113.9(13)
S03	Li18	S06	90.7(10)
S03	Li18	S12	90.6(11)
S03	Li19	Li20	128(6)
S03	Li19	Li22	131(9)
S03	Li20	Li19	103(10)
S03	Li20	Li21	65(6)
S03	Li20	Li3	144(12)
S03	Li20	S07	93(8)
S03	Li20	S09	126(8)
S03	Li20	S12	90(8)
S03	Li20	Si2	57(6)
S03	Li20	Zn3	144(12)
S03	Li21	Li20	63(6)
S03	Li21	S14	115.6(14)
S03	Li8	Li17	62(10)
S03	Li8	S08	118.0(14)
S03	Si2	Li20	70(6)
S03	Si2	S07	111.0(4)
S03	Si2	S11	106.7(4)
S03	Si2	S12	107.1(4)
S04	Li10	Li11	60.7(14)
S04	Li10	Li4	62.8(14)
S04	Li10	Li7	165(3)
S04	Li10	S09	104.7(18)
S04	Li10	S14	117(2)
S04	Li10	Zn4	62.8(14)

Li17	S07	Li16	30(10)
Li17	S07	Li20	74(11)
Li17	S07	Li3	101(10)
Li17	S07	Si2	77(10)
Li17	S07	Zn3	101(10)
Li17	S07	Zn6	30(10)
Li17	S08	Li12	91(10)
Li17	S08	Li16	30(10)
Li17	S08	Li5	119(10)
Li17	S08	Li6	66(10)
Li17	S08	Li8	58(10)
Li17	S08	Li9	153(10)
Li17	S08	Zn6	30(10)
Li17	S14	Li1	114(10)
Li17	S14	Li11	149(10)
Li17	S14	Li21	61(10)
Li17	S14	Zn1	114(10)
Li17	Si2	Li12	93(9)
Li17	Si2	Li20	72(11)
Li17	Si2	S03	57(9)
Li17	Si2	S07	58(9)
Li17	Si2	S11	105(9)
Li17	Si2	S12	143(9)
Li18	Li19	Li20	77(6)
Li18	Li19	Li22	158(9)
Li18	Li19	Li4	91.2(12)
Li18	Li19	Li8	90.0(14)
Li18	Li19	S01	136.4(16)
Li18	Li19	S03	52.4(10)
Li18	Li19	S09	92.7(13)
Li18	Li19	S12	50.0(9)
Li18	Li19	Zn4	91.2(12)
Li18	Li21	Li1	138.5(14)
Li18	Li21	Li17	111(10)
Li18	Li21	Li2	92.0(12)
Li18	Li21	Li20	84(7)
Li18	Li21	Li22	45(8)
Li18	Li21	S03	52.8(9)
Li18	Li21	S06	50.0(9)
Li18	Li21	S09	102.9(13)
Li18	Li21	S14	144.2(16)
Li18	Li21	Zn1	138.5(14)
Li18	Li21	Zn2	92.0(12)
Li18	Li22	Li19	94(13)
Li18	Li6	Li10	100.2(16)
Li18	Li6	Li16	137.1(14)
S04	Li11	Li10	62.7(14)
S04	Li11	S14	121.9(12)
S04	Li11	Zn5	62.7(14)
S04	Li4	Li10	70.3(16)
S04	Li4	S09	113.8(5)
S04	Li4	Zn5	70.3(16)
S04	Li8	Li17	103(10)
S04	Li8	S03	102.6(12)
S04	Li8	S08	115.7(13)
S04	Si1	S05	109.3(4)
S04	Si1	S06	110.5(4)
S04	Zn4	Li10	70.3(16)
S04	Zn4	S09	113.8(5)
S04	Zn4	Zn5	70.3(16)
S04	Zn5	Li11	60.7(14)
S04	Zn5	Li4	62.8(14)
S04	Zn5	Li7	165(3)
S04	Zn5	S09	104.7(18)
S04	Zn5	S14	117(2)
S04	Zn5	Zn4	62.8(14)
S05	Li1	S05	95.1(3)
S05	Li1	S09	109.0(3)
S05	Li1	S14	106.5(3)
S05	Li11	Li10	97.2(16)
S05	Li11	S04	85.9(9)
S05	Li11	S14	119.8(11)
S05	Li11	Zn5	97.2(16)
S05	Si1	S06	109.0(4)
S05	Zn1	S05	95.1(3)
S05	Zn1	S09	109.0(3)
S05	Zn1	S14	106.5(3)
S06	Li18	Li22	67(9)
S06	Li18	S01	131.4(14)
S06	Li2	S02	114.7(5)
S06	Li2	S04	96.2(5)
S06	Li21	Li20	133(7)
S06	Li21	S03	91.8(12)
S06	Li21	S09	111.9(13)
S06	Li21	S14	103.8(13)
S06	Li22	Li13	125(15)
S06	Li22	Li18	60(9)
S06	Li22	Li19	137(16)
S06	Li22	S10	83(10)
S06	Li6	Li16	167.1(17)
S06	Li6	S08	114.4(14)
S06	Li6	S12	89.2(12)

Li18	Li6	Li17	150(9)	S06	Li6	Zn6	167.1(17)
Li18	Li6	S06	50.3(9)	S06	Zn2	S02	114.7(5)
Li18	Li6	S08	93.1(12)	S06	Zn2	S04	96.2(5)
Li18	Li6	S12	51.7(9)	S07	Li12	S11	88.5(10)
Li18	Li6	S14	154.0(15)	S07	Li12	S15	113.7(13)
Li18	Li6	Zn5	100.2(16)	S07	Li16	Li17	78(19)
Li18	Li6	Zn6	137.1(14)	S07	Li16	S11	95.1(8)
Li18	Li9	Li12	113.5(14)	S07	Li17	Li16	71(18)
Li18	Li9	Li3	70.0(9)	S07	Li17	Li21	92(14)
Li18	Li9	Li5	100.9(12)	S07	Li17	Li8	128(17)
Li18	Li9	S01	49.2(8)	S07	Li17	S03	89(14)
Li18	Li9	S07	93.0(11)	S07	Li17	S14	114(17)
Li18	Li9	S08	94.3(12)	S07	Li17	Zn6	71(18)
Li18	Li9	S13	150.8(15)	S07	Li20	Li19	152(11)
Li18	Li9	Zn3	70.0(9)	S07	Li20	Li21	105(11)
Li18	S01	Li13	96.2(11)	S07	Li20	Li3	61(6)
Li18	S03	Li17	135(10)	S07	Li20	S09	119(11)
Li18	S03	Li19	77.9(12)	S07	Li20	S12	90(6)
Li18	S03	Li20	94(5)	S07	Li20	Si2	55(5)
Li18	S03	Li21	76.9(11)	S07	Li20	Zn3	61(6)
Li18	S03	Li8	122.3(11)	S07	Li3	Li20	60(5)
Li18	S03	Si2	130.5(8)	S07	Li3	S10	103.2(5)
Li18	S06	Si1	125.3(8)	S07	Li9	S08	103.6(13)
Li18	S12	Li20	89(5)	S07	Li9	S13	90.0(11)
Li18	S12	Li4	126.0(9)	S07	Si2	Li20	71(7)
Li18	S12	Li6	76.9(12)	S07	Si2	S12	110.3(4)
Li18	S12	Si2	122.3(8)	S07	Zn3	Li20	60(5)
Li18	S12	Zn4	126.0(9)	S07	Zn3	S10	103.2(5)
Li19	Li18	Li21	98.5(13)	S07	Zn6	Li17	78(19)
Li19	Li18	Li22	146(9)	S07	Zn6	S11	95.1(8)
Li19	Li18	Li6	102.6(14)	S08	Li12	S07	104.2(12)
Li19	Li18	Li9	112.1(14)	S08	Li12	S11	107.8(13)
Li19	Li18	S01	108.9(13)	S08	Li12	S15	120.1(13)
Li19	Li18	S03	49.8(9)	S08	Li14	Li15	106(3)
Li19	Li18	S06	118.6(14)	S08	Li14	S11	111.2(15)
Li19	Li18	S12	52.6(10)	S08	Li14	S13	116.3(16)
Li19	Li20	Li21	102(8)	S08	Li16	Li17	78(19)
Li19	Li20	Si2	117(12)	S08	Li16	S07	113.0(8)
Li19	Li4	Li10	123.8(18)	S08	Li16	S11	107.7(7)
Li19	Li4	Li3	116.8(9)	S08	Li17	Li16	72(18)
Li19	Li4	Li8	61.1(10)	S08	Li17	Li21	160(20)
Li19	Li4	S01	55.9(8)	S08	Li17	Li8	59(10)
Li19	Li4	S04	103.4(9)	S08	Li17	S03	112(16)
Li19	Li4	S09	142.7(10)	S08	Li17	S07	108(16)
Li19	Li4	S12	55.1(8)	S08	Li17	S14	116(16)
Li19	Li4	Zn3	116.8(9)	S08	Li17	Zn6	72(18)

Li19	Li4	Zn5	123.8(18)
Li19	Li8	Li17	113(10)
Li19	Li8	Li4	59.6(10)
Li19	Li8	Li5	114.0(16)
Li19	Li8	S01	54.6(10)
Li19	Li8	S03	52.0(10)
Li19	Li8	S04	100.6(13)
Li19	Li8	S08	143.6(15)
Li19	Li8	Zn4	59.6(10)
Li19	S01	Li13	81.3(12)
Li19	S01	Li18	82.2(12)
Li19	S01	Li3	131.1(9)
Li19	S01	Li4	72.2(9)
Li19	S01	Li5	130.4(12)
Li19	S01	Li8	72.1(12)
Li19	S01	Li9	149.3(12)
Li19	S01	Zn3	131.1(9)
Li19	S01	Zn4	72.2(9)
Li19	S03	Li20	146(6)
Li19	S03	Li8	76.5(12)
Li19	S03	Si2	109.2(9)
Li19	S09	Li1	146.1(10)
Li19	S09	Li10	117.3(15)
Li19	S09	Li20	52(7)
Li19	S09	Li21	84.3(12)
Li19	S09	Li3	97.3(9)
Li19	S09	Li4	98.3(10)
Li19	S09	Li7	91.5(12)
Li19	S09	Zn1	146.1(10)
Li19	S09	Zn3	97.3(9)
Li19	S09	Zn4	98.3(10)
Li19	S09	Zn5	117.3(15)
Li19	S12	Li18	77.5(12)
Li19	S12	Li20	52(6)
Li19	S12	Li4	73.1(10)
Li19	S12	Li6	150.0(13)
Li19	S12	Si2	102.4(10)
Li19	S12	Zn4	73.1(10)
Li19	Zn4	Li10	123.8(18)
Li19	Zn4	Li3	116.8(9)
Li19	Zn4	Li8	61.1(10)
Li19	Zn4	S01	55.9(8)
Li19	Zn4	S04	103.4(9)
Li19	Zn4	S09	142.7(10)
Li19	Zn4	S12	55.1(8)
Li19	Zn4	Zn3	116.8(9)
S08	Li5	S10	119.4(13)
S08	Li5	S13	119.2(13)
S08	Li6	Li16	59.2(9)
S08	Li6	S12	108.7(12)
S08	Li6	Zn6	59.2(9)
S08	Li8	Li17	63(10)
S08	Zn6	Li17	78(19)
S08	Zn6	S07	113.0(8)
S08	Zn6	S11	107.7(7)
S09	Li1	S05	115.2(3)
S09	Li10	Li11	121(2)
S09	Li10	Li4	62.8(14)
S09	Li10	Li7	64.2(16)
S09	Li10	S14	121.5(19)
S09	Li10	Zn4	62.8(14)
S09	Li19	Li20	59(6)
S09	Li19	Li22	66(10)
S09	Li19	S03	106.7(15)
S09	Li19	S12	113.8(15)
S09	Li20	Li19	69(6)
S09	Li20	Li21	65(5)
S09	Li20	Li3	61(6)
S09	Li20	Si2	173(15)
S09	Li20	Zn3	61(6)
S09	Li21	Li20	61(6)
S09	Li21	S03	120.4(14)
S09	Li21	S14	110.4(14)
S09	Li22	Li13	123(14)
S09	Li22	Li18	127(15)
S09	Li22	Li19	64(9)
S09	Li22	S01	128(14)
S09	Li22	S06	103(12)
S09	Li22	S10	105(13)
S09	Li3	Li20	60(5)
S09	Li3	S07	117.3(6)
S09	Li3	S10	114.3(5)
S09	Li4	Li10	70.3(16)
S09	Li4	Zn5	70.3(16)
S09	Li7	Li10	64.2(16)
S09	Li7	S10	112.6(16)
S09	Li7	S11	114.3(14)
S09	Li7	Zn5	64.2(16)
S09	Zn1	S05	115.2(3)
S09	Zn3	Li20	60(5)
S09	Zn3	S07	117.3(6)
S09	Zn3	S10	114.3(5)

Li19	Zn4	Zn5	123.8(18)
Li2	Li11	Li10	114.6(17)
Li2	Li11	Li2	83.4(9)
Li2	Li11	S02	53.8(6)
Li2	Li11	S04	131.7(12)
Li2	Li11	S05	138.4(13)
Li2	Li11	S14	60.4(7)
Li2	Li11	Zn2	83.4(9)
Li2	Li11	Zn5	114.6(17)
Li2	Li21	Li1	86.6(10)
Li2	Li21	Li17	83(10)
Li2	Li21	Li20	159(6)
Li2	Li21	Li22	112(8)
Li2	Li21	S03	98.9(13)
Li2	Li21	S06	52.2(7)
Li2	Li21	S09	139.2(14)
Li2	Li21	S14	54.0(7)
Li2	Li21	Zn1	86.6(10)
Li2	S02	Li2	151.2(6)
Li2	S04	Si1	108.3(5)
Li2	S06	Li18	129.0(8)
Li2	S06	Li21	76.1(9)
Li2	S06	Si1	104.6(4)
Li2	S14	Li1	100.3(4)
Li2	S14	Li10	111.8(13)
Li2	S14	Li11	63.0(7)
Li2	S14	Li16	120.8(6)
Li2	S14	Li17	96(10)
Li2	S14	Li21	75.2(9)
Li2	S14	Zn1	100.3(4)
Li2	S14	Zn5	111.8(13)
Li2	S14	Zn6	120.8(6)
Li20	Li17	Li16	110(20)
Li20	Li17	Li21	46(8)
Li20	Li17	Li6	155(18)
Li20	Li17	Li8	111(16)
Li20	Li17	S03	52(10)
Li20	Li17	S07	51(9)
Li20	Li17	S08	148(17)
Li20	Li17	S14	96(14)
Li20	Li17	Si2	41(7)
Li20	Li17	Zn6	110(20)
Li20	Li22	Li13	137(15)
Li20	Li22	Li18	83(11)
Li20	Li22	Li19	44(8)
Li20	Li22	Li21	43(7)
S09	Zn4	Li10	70.3(16)
S09	Zn4	Zn5	70.3(16)
S09	Zn5	Li11	121(2)
S09	Zn5	Li4	62.8(14)
S09	Zn5	Li7	64.2(16)
S09	Zn5	S14	121.5(19)
S09	Zn5	Zn4	62.8(14)
S10	Li13	Li22	67(9)
S10	Li13	S01	128.1(13)
S10	Li22	Li13	59(8)
S10	Li22	Li18	119(14)
S10	Li22	Li19	139(16)
S10	Li3	Li20	144(6)
S10	Li5	S13	93.4(11)
S10	Li7	Li10	166(2)
S10	Li7	Zn5	166(2)
S10	Si1	S04	110.2(4)
S10	Si1	S05	109.4(4)
S10	Si1	S06	108.4(4)
S10	Zn3	Li20	144(6)
S11	Li12	S15	117.6(13)
S11	Li13	Li22	103(9)
S11	Li13	S01	124.3(14)
S11	Li13	S10	89.6(11)
S11	Li14	Li15	142(2)
S11	Li14	S13	93.7(13)
S11	Li16	Li17	173(19)
S11	Li7	Li10	106(2)
S11	Li7	S10	88.1(12)
S11	Li7	Zn5	106(2)
S11	Si2	Li20	176(6)
S11	Si2	S07	109.2(4)
S11	Si2	S12	112.4(4)
S11	Zn6	Li17	173(19)
S12	Li10	Li11	128(2)
S12	Li10	Li4	63.8(14)
S12	Li10	Li7	103(2)
S12	Li10	S04	90.7(16)
S12	Li10	S09	107.3(18)
S12	Li10	S14	110.7(19)
S12	Li10	Zn4	63.8(14)
S12	Li18	Li22	157(9)
S12	Li18	S01	127.0(14)
S12	Li18	S06	91.7(11)
S12	Li19	Li20	60(5)
S12	Li19	Li22	138(9)

Li20	Li22	Li7	103(12)
Li20	Li22	S01	93(11)
Li20	Li22	S06	96(12)
Li20	Li22	S09	48(7)
Li20	Li22	S10	152(15)
Li20	Li22	Si1	122(13)
Li20	S03	Si2	53(4)
Li20	S07	Si2	54(5)
Li20	S12	Si2	55(6)
Li20	Si2	S12	71(7)
Li21	Li1	S05	99.9(8)
Li21	Li1	S09	59.7(8)
Li21	Li1	S14	58.6(8)
Li21	Li17	Li16	120(30)
Li21	Li17	Li8	102(16)
Li21	Li17	S03	59(10)
Li21	Li17	S14	59(10)
Li21	Li17	Zn6	120(30)
Li21	Li18	Li22	57(9)
Li21	Li18	Li6	103.1(13)
Li21	Li18	Li9	149.4(15)
Li21	Li18	S01	114.2(13)
Li21	Li18	S03	50.3(9)
Li21	Li18	S06	51.2(9)
Li21	Li18	S12	117.4(13)
Li21	Li2	Li11	140.0(10)
Li21	Li2	S02	147.9(8)
Li21	Li2	S04	99.0(8)
Li21	Li2	S06	51.7(7)
Li21	Li2	S14	50.7(7)
Li21	Li20	Si2	115(11)
Li21	Li22	Li13	180(20)
Li21	Li22	Li18	78(11)
Li21	Li22	Li19	87(12)
Li21	Li22	S01	123(13)
Li21	Li22	S06	56(7)
Li21	Li22	S09	55(7)
Li21	Li22	S10	120(14)
Li21	S03	Li19	149.4(13)
Li21	S03	Li20	53(5)
Li21	S03	Li8	103.0(12)
Li21	S03	Si2	100.4(9)
Li21	S06	Li18	78.8(11)
Li21	S06	Si1	108.5(9)
Li21	S09	Li1	63.0(8)
Li21	S09	Li20	53(5)
S12	Li19	S03	90.6(13)
S12	Li20	Li19	68(8)
S12	Li20	Li21	151(11)
S12	Li20	Li3	113(8)
S12	Li20	S09	129(11)
S12	Li20	Si2	54(5)
S12	Li20	Zn3	113(8)
S12	Li4	Li10	69.9(16)
S12	Li4	S04	96.9(5)
S12	Li4	S09	115.9(5)
S12	Li4	Zn5	69.9(16)
S12	Zn4	Li10	69.9(16)
S12	Zn4	S04	96.9(5)
S12	Zn4	S09	115.9(5)
S12	Zn4	Zn5	69.9(16)
S12	Zn5	Li11	128(2)
S12	Zn5	Li4	63.8(14)
S12	Zn5	Li7	103(2)
S12	Zn5	S04	90.7(16)
S12	Zn5	S09	107.3(18)
S12	Zn5	S14	110.7(19)
S12	Zn5	Zn4	63.8(14)
S13	Li13	Li22	152(9)
S13	Li13	S01	120.9(12)
S13	Li13	S10	90.8(11)
S13	Li13	S11	93.3(11)
S13	Li14	Li15	64(6)
S13	Li15	Li14	72(3)
S13	Li15	S13	99(9)
S13	Li15	S15	131(5)
S13	Li9	S08	113.2(12)
S13	Si3	S13	108.25(17)
S14	Li1	S05	116.7(3)
S14	Li1	S09	112.2(3)
S14	Li10	Li11	60.0(13)
S14	Li10	Li4	174.4(12)
S14	Li10	Li7	65.3(16)
S14	Li10	Zn4	174.4(12)
S14	Li11	Li10	62.9(14)
S14	Li11	Zn5	62.9(14)
S14	Li16	Li17	72(19)
S14	Li16	Li6	62.0(9)
S14	Li16	S07	114.0(8)
S14	Li16	S08	115.6(9)
S14	Li16	S11	109.2(8)
S14	Li17	Li16	80(20)

Li21	S09	Li3	88.5(9)
Li21	S09	Li4	152.4(9)
Li21	S09	Zn1	63.0(8)
Li21	S09	Zn3	88.5(9)
Li21	S09	Zn4	152.4(9)
Li21	Zn1	S05	99.9(8)
Li21	Zn1	S09	59.7(8)
Li21	Zn1	S14	58.6(8)
Li21	Zn2	Li11	140.0(10)
Li21	Zn2	S02	147.9(8)
Li21	Zn2	S04	99.0(8)
Li21	Zn2	S06	51.7(7)
Li21	Zn2	S14	50.7(7)
Li22	Li19	Li20	91(11)
Li22	Li20	Li17	110(12)
Li22	Li20	Li19	45(8)
Li22	Li20	Li21	57(8)
Li22	Li20	Li3	116(10)
Li22	Li20	S03	81(9)
Li22	Li20	S07	162(12)
Li22	Li20	S09	55(8)
Li22	Li20	S12	107(12)
Li22	Li20	Si2	131(12)
Li22	Li20	Zn3	116(10)
Li22	Li21	Li1	97(8)
Li22	Li21	Li17	149(13)
Li22	Li21	Li20	80(10)
Li22	Li21	S03	91(8)
Li22	Li21	S06	60(8)
Li22	Li21	S09	61(8)
Li22	Li21	S14	151(8)
Li22	Li21	Zn1	97(8)
Li22	Li7	Li10	122(7)
Li22	Li7	S09	59(7)
Li22	Li7	S10	59(7)
Li22	Li7	S11	89(8)
Li22	Li7	S14	162(7)
Li22	Li7	Zn5	122(7)
Li22	S01	Li13	54(8)
Li22	S01	Li18	53(8)
Li22	S01	Li19	51(8)
Li22	S01	Li3	142(8)
Li22	S01	Li4	113(8)
Li22	S01	Li5	142(8)
Li22	S01	Li8	112(8)
Li22	S01	Li9	99(8)
S14	Li17	Li8	116(17)
S14	Li17	Zn6	80(20)
S14	Li2	S02	119.7(5)
S14	Li2	S04	110.9(5)
S14	Li2	S06	100.2(5)
S14	Li21	Li20	123(7)
S14	Li6	Li16	60.3(10)
S14	Li6	S06	117.8(12)
S14	Li6	S08	112.4(13)
S14	Li6	S12	112.0(14)
S14	Li6	Zn6	60.3(10)
S14	Li7	Li10	63.3(16)
S14	Li7	S09	119.8(15)
S14	Li7	S10	111.1(14)
S14	Li7	S11	106.3(15)
S14	Li7	Zn5	63.3(16)
S14	Zn1	S05	116.7(3)
S14	Zn1	S09	112.2(3)
S14	Zn2	S02	119.7(5)
S14	Zn2	S04	110.9(5)
S14	Zn2	S06	100.2(5)
S14	Zn5	Li11	60.0(13)
S14	Zn5	Li4	174.4(12)
S14	Zn5	Li7	65.3(16)
S14	Zn5	Zn4	174.4(12)
S14	Zn6	Li17	72(19)
S14	Zn6	Li6	62.0(9)
S14	Zn6	S07	114.0(8)
S14	Zn6	S08	115.6(9)
S14	Zn6	S11	109.2(8)
S15	Li14	Li15	61(7)
S15	Li14	S08	111.9(14)
S15	Li14	S11	108.7(15)
S15	Li14	S13	113.5(15)
S15	Li15	Li14	76(7)
Si1	Li11	Li10	86.3(15)
Si1	Li11	Li2	78.3(8)
Si1	Li11	S02	99.2(9)
Si1	Li11	S04	44.2(5)
Si1	Li11	S05	43.7(5)
Si1	Li11	S14	144.8(12)
Si1	Li11	Zn2	78.3(8)
Si1	Li11	Zn5	86.3(15)
Si1	Li22	Li13	100(12)
Si1	Li22	Li18	97(12)
Si1	Li22	Li19	161(17)

Li22	S01	Zn3	142(8)
Li22	S01	Zn4	113(8)
Li22	S06	Li18	53(8)
Li22	S06	Li2	139(8)
Li22	S06	Li21	64(8)
Li22	S06	Li6	112(8)
Li22	S06	Si1	81(8)
Li22	S06	Zn2	139(8)
Li22	S09	Li1	103(8)
Li22	S09	Li10	116(8)
Li22	S09	Li19	51(8)
Li22	S09	Li20	78(10)
Li22	S09	Li21	63(8)
Li22	S09	Li3	137(8)
Li22	S09	Li4	138(8)
Li22	S09	Li7	65(8)
Li22	S09	Zn1	103(8)
Li22	S09	Zn3	137(8)
Li22	S09	Zn4	138(8)
Li22	S09	Zn5	116(8)
Li22	S10	Li13	54(8)
Li22	S10	Li3	172(8)
Li22	S10	Li5	108(8)
Li22	S10	Li7	67(8)
Li22	S10	Si1	80(8)
Li22	S10	Zn3	172(8)
Li22	Si1	Li11	155(7)
Li22	Si1	S04	146(7)
Li22	Si1	S05	105(7)
Li22	Si1	S06	57(7)
Li22	Si1	S10	56(7)
Li3	Li20	Li19	111(9)
Li3	Li20	Li21	97(10)
Li3	Li20	Si2	113(10)
Li3	Li4	Li10	119.1(17)
Li3	Li4	S01	62.0(4)
Li3	Li4	S04	101.9(5)
Li3	Li4	S09	58.2(5)
Li3	Li4	S12	161.0(6)
Li3	Li4	Zn5	119.1(17)
Li3	Li5	Li13	90.1(11)
Li3	Li5	Li8	93.6(13)
Li3	Li5	S01	55.3(7)
Li3	Li5	S08	149.1(14)
Li3	Li5	S10	51.4(7)
Li3	Li5	S13	91.6(11)

Si1	Li22	Li21	80(9)
Si1	Li22	Li7	75(9)
Si1	Li22	S01	135(13)
Si1	Li22	S06	42(5)
Si1	Li22	S09	97(11)
Si1	Li22	S10	43(5)
Si2	Li12	Li14	85.2(12)
Si2	Li12	Li16	82.3(10)
Si2	Li12	S07	45.1(5)
Si2	Li12	S08	99.9(11)
Si2	Li12	S11	46.2(6)
Si2	Li12	S15	139.6(13)
Si2	Li12	Zn6	82.3(10)
Si2	Li17	Li16	110(20)
Si2	Li17	Li21	82(12)
Si2	Li17	Li6	164(16)
Si2	Li17	Li8	88(13)
Si2	Li17	S03	47(8)
Si2	Li17	S07	45(7)
Si2	Li17	S08	107(15)
Si2	Li17	S14	137(17)
Si2	Li17	Zn6	110(20)
Si3	Li15	Li14	104(7)
Si3	Li15	S13	49(5)
Si3	Li15	S15	180
Zn1	Li21	Li17	110(10)
Zn1	Li21	Li20	110(6)
Zn1	Li21	S03	167.7(17)
Zn1	Li21	S06	100.3(12)
Zn1	Li21	S09	57.2(8)
Zn1	Li21	S14	59.1(8)
Zn1	S05	Si1	103.2(3)
Zn1	S05	Zn1	113.1(2)
Zn1	S09	Li20	108(6)
Zn1	S09	Li4	114.7(4)
Zn1	S09	Zn4	114.7(4)
Zn1	S14	Li11	92.9(7)
Zn1	S14	Li21	62.3(9)
Zn2	Li11	Li10	114.6(17)
Zn2	Li11	S02	53.8(6)
Zn2	Li11	S04	131.7(12)
Zn2	Li11	S05	138.4(13)
Zn2	Li11	S14	60.4(7)
Zn2	Li11	Zn2	83.4(9)
Zn2	Li11	Zn5	114.6(17)
Zn2	Li21	Li1	86.6(10)

Li3	Li9	Li12	104.4(12)	Zn2	Li21	Li17	83(10)
Li3	Li9	S01	54.7(6)	Zn2	Li21	Li20	159(6)
Li3	Li9	S07	50.9(6)	Zn2	Li21	Li22	112(8)
Li3	Li9	S08	147.0(14)	Zn2	Li21	S03	98.9(13)
Li3	Li9	S13	89.9(10)	Zn2	Li21	S06	52.2(7)
Li3	S01	Li13	147.5(9)	Zn2	Li21	S09	139.2(14)
Li3	S01	Li18	88.3(8)	Zn2	Li21	S14	54.0(7)
Li3	S01	Li4	60.4(5)	Zn2	Li21	Zn1	86.6(10)
Li3	S01	Li5	71.3(8)	Zn2	S02	Zn2	151.2(6)
Li3	S01	Li8	102.4(8)	Zn2	S04	Si1	108.3(5)
Li3	S01	Zn4	60.4(5)	Zn2	S06	Li18	129.0(8)
Li3	S07	Li20	59(5)	Zn2	S06	Li21	76.1(9)
Li3	S07	Si2	111.0(4)	Zn2	S06	Si1	104.6(4)
Li3	S09	Li1	91.0(4)	Zn2	S14	Li1	100.3(4)
Li3	S09	Li20	59(6)	Zn2	S14	Li10	111.8(13)
Li3	S09	Li4	63.9(5)	Zn2	S14	Li11	63.0(7)
Li3	S09	Zn1	91.0(4)	Zn2	S14	Li16	120.8(6)
Li3	S09	Zn4	63.9(5)	Zn2	S14	Li17	96(10)
Li3	S10	Si1	106.1(4)	Zn2	S14	Li21	75.2(9)
Li3	Zn4	Li10	119.1(17)	Zn2	S14	Zn1	100.3(4)
Li3	Zn4	S01	62.0(4)	Zn2	S14	Zn5	111.8(13)
Li3	Zn4	S04	101.9(5)	Zn2	S14	Zn6	120.8(6)
Li3	Zn4	S09	58.2(5)	Zn3	Li20	Li19	111(9)
Li3	Zn4	S12	161.0(6)	Zn3	Li20	Li21	97(10)
Li3	Zn4	Zn5	119.1(17)	Zn3	Li20	Si2	113(10)
Li4	Li19	Li20	96(6)	Zn3	Li4	Li10	119.1(17)
Li4	Li19	Li22	108(9)	Zn3	Li4	S01	62.0(4)
Li4	Li19	S01	51.9(8)	Zn3	Li4	S04	101.9(5)
Li4	Li19	S03	96.6(13)	Zn3	Li4	S09	58.2(5)
Li4	Li19	S09	153.3(17)	Zn3	Li4	S12	161.0(6)
Li4	Li19	S12	51.7(8)	Zn3	Li4	Zn5	119.1(17)
Li4	Li3	Li20	103(6)	Zn3	Li5	Li13	90.1(11)
Li4	Li3	S07	154.9(6)	Zn3	Li5	Li8	93.6(13)
Li4	Li3	S09	57.8(4)	Zn3	Li5	S01	55.3(7)
Li4	Li3	S10	100.6(6)	Zn3	Li5	S08	149.1(14)
Li4	Li8	Li17	145(10)	Zn3	Li5	S10	51.4(7)
Li4	Li8	Li5	87.3(13)	Zn3	Li5	S13	91.6(11)
Li4	Li8	S01	52.4(7)	Zn3	Li9	Li12	104.4(12)
Li4	Li8	S03	97.2(11)	Zn3	Li9	S01	54.7(6)
Li4	Li8	S04	51.3(7)	Zn3	Li9	S07	50.9(6)
Li4	Li8	S08	144.8(14)	Zn3	Li9	S08	147.0(14)
Li4	S01	Li13	150.8(9)	Zn3	Li9	S13	89.9(10)
Li4	S01	Li18	92.2(8)	Zn3	S01	Li13	147.5(9)
Li4	S04	Li2	142.7(5)	Zn3	S01	Li18	88.3(8)
Li4	S04	Si1	105.9(4)	Zn3	S01	Li4	60.4(5)
Li4	S04	Zn2	142.7(5)	Zn3	S01	Li5	71.3(8)

Li4	S09	Li20	107(5)	Zn3	S01	Li8	102.4(8)
Li4	S12	Li20	106(6)	Zn3	S01	Zn4	60.4(5)
Li4	S12	Si2	107.8(5)	Zn3	S07	Li20	59(5)
Li4	Zn3	Li20	103(6)	Zn3	S07	Si2	111.0(4)
Li4	Zn3	S07	154.9(6)	Zn3	S09	Li1	91.0(4)
Li4	Zn3	S09	57.8(4)	Zn3	S09	Li20	59(6)
Li4	Zn3	S10	100.6(6)	Zn3	S09	Li4	63.9(5)
Li5	Li13	Li14	102.3(14)	Zn3	S09	Zn1	91.0(4)
Li5	Li13	Li22	101(9)	Zn3	S09	Zn4	63.9(5)
Li5	Li13	S01	115.0(14)	Zn3	S10	Si1	106.1(4)
Li5	Li13	S10	53.0(9)	Zn3	Zn4	Li10	119.1(17)
Li5	Li13	S11	120.7(14)	Zn3	Zn4	S01	62.0(4)
Li5	Li13	S13	50.9(9)	Zn3	Zn4	S04	101.9(5)
Li5	Li3	Li20	154(5)	Zn3	Zn4	S09	58.2(5)
Li5	Li3	Li4	86.4(8)	Zn3	Zn4	S12	161.0(6)
Li5	Li3	Li9	61.6(10)	Zn3	Zn4	Zn5	119.1(17)
Li5	Li3	S01	53.4(6)	Zn4	Li19	Li20	96(6)
Li5	Li3	S07	101.8(8)	Zn4	Li19	Li22	108(9)
Li5	Li3	S09	140.9(8)	Zn4	Li19	S01	51.9(8)
Li5	Li3	S10	52.9(7)	Zn4	Li19	S03	96.6(13)
Li5	Li3	Zn4	86.4(8)	Zn4	Li19	S09	153.3(17)
Li5	Li8	Li17	123(10)	Zn4	Li19	S12	51.7(8)
Li5	Li8	S03	157.1(16)	Zn4	Li3	Li20	103(6)
Li5	Li8	S04	97.7(14)	Zn4	Li3	S07	154.9(6)
Li5	Li8	S08	60.3(10)	Zn4	Li3	S09	57.8(4)
Li5	Li9	Li12	133.9(15)	Zn4	Li3	S10	100.6(6)
Li5	Li9	Li3	59.2(9)	Zn4	Li8	Li17	145(10)
Li5	Li9	S01	52.4(8)	Zn4	Li8	Li5	87.3(13)
Li5	Li9	S07	96.9(12)	Zn4	Li8	S01	52.4(7)
Li5	Li9	S08	153.7(15)	Zn4	Li8	S03	97.2(11)
Li5	Li9	S13	49.9(9)	Zn4	Li8	S04	51.3(7)
Li5	Li9	Zn3	59.2(9)	Zn4	Li8	S08	144.8(14)
Li5	S01	Li13	88.8(10)	Zn4	S01	Li13	150.8(9)
Li5	S01	Li18	147.4(11)	Zn4	S01	Li18	92.2(8)
Li5	S01	Li4	98.8(8)	Zn4	S04	Li2	142.7(5)
Li5	S01	Zn4	98.8(8)	Zn4	S04	Si1	105.9(4)
Li5	S08	Li8	61.3(11)	Zn4	S04	Zn2	142.7(5)
Li5	S08	Li9	83.7(11)	Zn4	S09	Li20	107(5)
Li5	S10	Li13	75.0(11)	Zn4	S12	Li20	106(6)
Li5	S10	Li3	75.8(8)	Zn4	S12	Si2	107.8(5)
Li5	S10	Li7	152.3(12)	Zn4	Zn3	Li20	103(6)
Li5	S10	Si1	103.4(8)	Zn4	Zn3	S07	154.9(6)
Li5	S10	Zn3	75.8(8)	Zn4	Zn3	S09	57.8(4)
Li5	S13	Li15	166.3(8)	Zn4	Zn3	S10	100.6(6)
Li5	S13	Si3	106.7(8)	Zn5	Li6	Li16	89.4(17)
Li5	Zn3	Li20	154(5)	Zn5	Li6	Li17	104(9)

Li5	Zn3	Li4	86.4(8)
Li5	Zn3	Li9	61.6(10)
Li5	Zn3	S01	53.4(6)
Li5	Zn3	S07	101.8(8)
Li5	Zn3	S09	140.9(8)
Li5	Zn3	S10	52.9(7)
Li5	Zn3	Zn4	86.4(8)
Li6	Li10	Li11	86.6(18)
Li6	Li10	Li4	116(2)
Li6	Li10	Li7	94(2)
Li6	Li10	S04	99.9(19)
Li6	Li10	S09	150(2)
Li6	Li10	S12	54.9(13)
Li6	Li10	S14	58.4(13)
Li6	Li10	Zn4	116(2)
Li6	Li16	Li17	87(19)
Li6	Li16	S07	165.3(12)
Li6	Li16	S08	61.3(10)
Li6	Li16	S11	99.7(11)
Li6	Li17	Li16	65(16)
Li6	Li17	Li21	113(16)
Li6	Li17	Li8	82(13)
Li6	Li17	S03	135(18)
Li6	Li17	S07	136(17)
Li6	Li17	S08	57(9)
Li6	Li17	S14	59(9)
Li6	Li17	Zn6	65(16)
Li6	Li18	Li22	106(9)
Li6	Li18	S01	125.7(14)
Li6	Li18	S03	120.3(13)
Li6	Li18	S06	53.8(9)
Li6	Li18	S12	51.4(9)
Li6	S06	Li18	75.9(11)
Li6	S06	Li2	106.7(8)
Li6	S06	Li21	149.0(12)
Li6	S06	Si1	100.7(8)
Li6	S06	Zn2	106.7(8)
Li6	S08	Li12	99.3(11)
Li6	S08	Li16	59.5(10)
Li6	S08	Li5	108.7(12)
Li6	S08	Li8	86.8(11)
Li6	S08	Li9	94.9(11)
Li6	S08	Zn6	59.5(10)
Li6	S12	Li20	142(6)
Li6	S12	Li4	110.7(9)
Li6	S12	Si2	104.4(9)
Zn5	Li6	S06	99.8(15)
Zn5	Li6	S08	143.8(19)
Zn5	Li6	S12	58.4(13)
Zn5	Li6	S14	56.2(12)
Zn5	Li6	Zn6	89.4(17)
Zn5	S04	Li11	56.6(13)
Zn5	S04	Li2	108.7(12)
Zn5	S04	Li4	46.9(11)
Zn5	S04	Li8	119.1(13)
Zn5	S04	Si1	105.0(12)
Zn5	S04	Zn2	108.7(12)
Zn5	S04	Zn4	46.9(11)
Zn5	S09	Li1	92.0(12)
Zn5	S09	Li20	154(5)
Zn5	S09	Li21	152.5(14)
Zn5	S09	Li3	104.2(11)
Zn5	S09	Li4	47.0(11)
Zn5	S09	Zn1	92.0(12)
Zn5	S09	Zn3	104.2(11)
Zn5	S09	Zn4	47.0(11)
Zn5	S12	Li18	125.6(14)
Zn5	S12	Li19	118.3(14)
Zn5	S12	Li20	144(6)
Zn5	S12	Li4	46.2(11)
Zn5	S12	Li6	66.8(13)
Zn5	S12	Si2	105.4(13)
Zn5	S12	Zn4	46.2(11)
Zn5	S14	Li1	111.7(12)
Zn5	S14	Li11	57.1(14)
Zn5	S14	Li17	120(10)
Zn5	S14	Li21	172.0(15)
Zn5	S14	Zn1	111.7(12)
Zn6	Li12	Li14	100.6(14)
Zn6	Li12	S07	122.7(14)
Zn6	Li12	S08	57.2(9)
Zn6	Li12	S11	56.8(9)
Zn6	Li12	S15	122.5(13)
Zn6	Li6	S12	103.4(14)
Zn6	Li7	Li10	91.2(19)
Zn6	Li7	Li22	136(8)
Zn6	Li7	S09	149.5(17)
Zn6	Li7	S10	95.5(13)
Zn6	Li7	S11	52.8(8)
Zn6	Li7	S14	54.9(9)
Zn6	Li7	Zn5	91.2(19)
Zn6	S07	Li20	100(6)

Li6	S12	Zn4	110.7(9)	Zn6	S07	Li3	100.9(6)
Li6	S14	Li1	174.7(9)	Zn6	S07	Si2	105.5(6)
Li6	S14	Li10	65.4(14)	Zn6	S07	Zn3	100.9(6)
Li6	S14	Li11	88.9(10)	Zn6	S08	Li5	148.5(9)
Li6	S14	Li16	57.7(9)	Zn6	S08	Li8	87.9(10)
Li6	S14	Li17	66(10)	Zn6	S08	Li9	124.3(10)
Li6	S14	Li2	84.9(9)	Zn6	S11	Li12	66.7(9)
Li6	S14	Li21	120.2(12)	Zn6	S11	Si2	106.3(6)
Li6	S14	Li7	90.4(12)	Zn6	S14	Li1	119.1(6)
Li6	S14	Zn1	174.7(9)	Zn6	S14	Li10	93.4(13)
Li6	S14	Zn2	84.9(9)	Zn6	S14	Li11	143.9(9)
Li6	S14	Zn5	65.4(14)	Zn6	S14	Li17	30(10)
Li6	S14	Zn6	57.7(9)	Zn6	S14	Li21	85.7(9)
Li6	Zn5	Li11	86.6(18)	Zn6	S14	Zn1	119.1(6)
Li6	Zn5	Li4	116(2)	Zn6	S14	Zn5	93.4(13)

Table S7. Comparison of distances between different Li sites in high-temperature $F\bar{4}3m$ $Li_7Zn_{0.5}SiS_6$, room-temperature $I\bar{4}$ $Li_7Zn_{0.5}SiS_6$, $F\bar{4}3m$ Li_6PS_5X (X = Cl, Br),[6] and $F\bar{4}3m$ $Li_{6.6}P_{0.4}Ge_{0.6}S_5I$.[7] Distances are not given where they do not exist (i.e. where sites are not occupied in those materials). For room-temperature $I\bar{4}$ $Li_7Zn_{0.5}SiS_6$ the shortest distances between different sites within the structure are given.

**Distance (Å)**	**$F\bar{4}3m$ $Li_7Zn_{0.5}SiS_6$ (T = 448 K)**	**$I\bar{4}$ $Li_7Zn_{0.5}SiS_6$ (T = 300 K)**	**Li_6PS_5Cl (T = 300 K)**	**Li_6PS_5Br (T = 300 K)**	**$Li_{6.6}P_{0.4}Ge_{0.6}S_5I$ (T = 150 K)**
T5-T5 intracage	1.896(7)	1.98(5)	2.003	1.540	1.26
T5-T2 intracage	1.281(14)	1.3(4)	1.45	1.33	1.51
T5-T4 intercage	1.643(5)	2.98(4)	-	-	1.93
T2-T2 intercage	2.11(9)	3.6(5)	1.32	2.03	1.93
T5-T1	-	2.2(2)	-	-	-
T5-T2a	-	1.82(7)	-	-	-

References

1. Ishii, M.; Onoda, M.; Shibata, K., Structure and vibrational spectra of argyrodite family compounds Cu_8SiX_6 (X=S, Se) and Cu_8GeS_6. *Solid State Ionics* **1999,** *121*, 11-18.
2. Deiseroth, H.-J.; Maier, J.; Weichert, K.; Nickel, V.; Kong, S.-T.; Reiner, C., Li_7PS_6 and Li_6PS_5X (*X*: Cl, Br, I): Possible Three-dimensional Diffusion Pathways for Lithium Ions and Temperature Dependence of the Ionic Conductivity by Impedance Measurements. *Z. Anorg. Allg. Chem.* **2011,** *637*, 1287-1294.
3. Strauss, F.; Zinkevich, T.; Indris, S.; Brezesinski, T., Li_7GeS_5Br—An Argyrodite Li-Ion Conductor Prepared by Mechanochemical Synthesis. *Inorg. Chem.* **2020,** *59*, 12954-12959.
4. Wang, S.; Zhang, Y.; Zhang, X.; Liu, T.; Lin, Y.-H.; Shen, Y.; Li, L.; Nan, C.-W., High-Conductivity Argyrodite Li_6PS_5Cl Solid Electrolytes Prepared via Optimized Sintering Processes for All-Solid-State Lithium–Sulfur Batteries. *ACS Appl. Mater. Interfaces* **2018,** *10*, 42279-42285.

5. Zhou, L.; Park, K.-H.; Sun, X.; Lalère, F.; Adermann, T.; Hartmann, P.; Nazar, L. F., Solvent-Engineered Design of Argyrodite Li_6PS_5X (X = Cl, Br, I) Solid Electrolytes with High Ionic Conductivity. *ACS Energy Lett.* **2019,** *4*, 265-270.
6. Minafra, N.; Kraft, M. A.; Bernges, T.; Li, C.; Schlem, R.; Morgan, B. J.; Zeier, W. G., Local Charge Inhomogeneity and Lithium Distribution in the Superionic Argyrodites Li_6PS_5X (X = Cl, Br, I). *Inorg. Chem.* **2020,** *59*, 11009-11019.
7. Hogrefe, K.; Minafra, N.; Hanghofer, I.; Banik, A.; Zeier, W. G.; Wilkening, H. M. R., Opening Diffusion Pathways through Site Disorder: The Interplay of Local Structure and Ion Dynamics in the Solid Electrolyte $Li_{6+x}P_{1-x}Ge_xS_5I$ as Probed by Neutron Diffraction and NMR. *J. Am. Chem. Soc.* **2022,** *144*, 1795-1812.
